# *De Novo*-Designed APC/C Inhibitors Provide a Rationale for Targeting RING-Type E3 Ubiquitin Ligases

**DOI:** 10.1021/acs.jmedchem.5c00416

**Published:** 2025-05-21

**Authors:** Gloria Ruiz-Gómez, Alena Uvizl, Gabor Bakos, Jacky K. Leung, M. Teresa Pisabarro, Jörg Mansfeld

**Affiliations:** † Structural Bioinformatics, Biotechnology Center (BIOTEC), TU Dresden, 01307 Dresden, Germany; ‡ Cell Cycle, Biotechnology Center (BIOTEC), TU Dresden, 01307 Dresden, Germany; § Division of Cell and Molecular Biology, Chester Beatty Laboratories, 5053 The Institute of Cancer Research, SW3 6JB London, U.K.

## Abstract

The ubiquitin system represents an attractive pharmacological target for numerous pathological processes, including cancer and neurodegeneration. RING domain-containing E3 ubiquitin ligases constitute the largest class of ubiquitin enzymes, providing a scaffold for substrate recognition and catalysis. Their shallow groove recognition interfaces involving discontinuous epitopes and a lack of defined binding pockets have largely rendered them undruggable. Inspired by natural RING inhibitors, we have developed a pharmacophore-based strategy for the rational design of peptidomimetics targeting RING domains, and we demonstrate its feasibility by using the macromolecular APC/C complex (anaphase-promoting complex/cyclosome). We designed scaffolds binding to the APC/C RING domain and efficiently inhibiting its activity *in vitro*. Iterative structure-based design and experimental studies to optimize their chemical stability, permeability, and specificity lead to new hydrocarbon-stapled-based molecules inhibiting APC/C *in vitro* and in cancer cells. Our results provide a robust rationale for targeting RING-containing enzymes of therapeutic value and promising leads for clinical APC/C inhibition.

## Graphical abstract

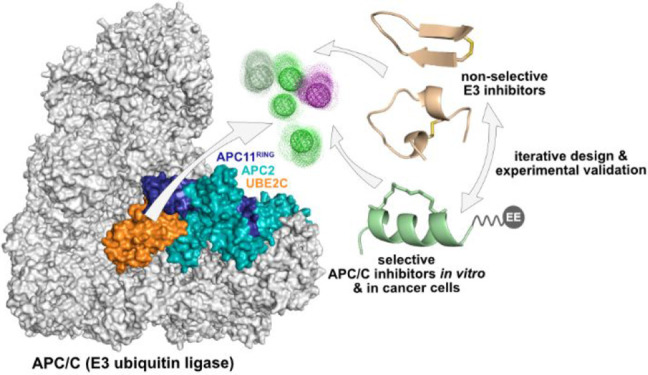

## Introduction

Protein post-translational modification with ubiquitin is vastly exploited in biology to expand the proteome complexity.^1^ In humans, more than 290000 experimentally identified ubiquitinylation sites^2^ are generated by the ordered interplay of three classes of enzymes. E1 ubiquitin-activating enzymes form a thioester bond between their active site cysteine and the carboxyl group of ubiquitin in an ATP-dependent manner. The activated ubiquitin is transferred to an E2 ubiquitin-conjugating enzyme that, in turn, interacts with an E3 ubiquitin ligase, which provides the scaffold for substrate recognition.^3^ The largest group of the more than 600 E3 ligases (E3s) encoded in the human genome contain a really interesting new gene (RING) domain^4^ and exhibits a crucial role in health and disease, reflecting the involvement of these enzymes in almost every cellular process. RING are zinc-finger type domains that catalyze the formation of an isopeptide bond between the lysine ε-amino of the substrate and the C-terminal carboxyl of ubiquitin delivered by E2 enzymes (E2s).^5^ Most RING-type E3s are cullin-RING ligases (CRLs) comprising a RING and a cullin domain, scaffold subunits, and substrate binding receptors.^3^

SKP1-CUL1-Fbox (SCF) and Anaphase Promoting Complex/Cyclosome (APC/C) are CRLs crucial for cell division, determining the proliferative potential of cells. SCF comprises 69 different human F-box proteins that ubiquitylate substrates (i.e., SCF-SKP2 targets cyclin-dependent kinase inhibitors CDKN1A (p21) and CDKN1B (p27)) for degradation to control G1/S transition.^6^ APC/C utilizes CDC20 and FZR1 (also named Cdh1) for activation and substrate recruitment.^7^ While both interact with APC/C through multiple domains, binding of their conserved C-terminal isoleucine-arginine tails (IR-tail) to the tetratricopeptide (TPR) subunit APC3 is crucial for substrate ubiquitinylation.^8^ SCF and APC/C employ two structurally highly related RING proteins (RBX1 and APC11, respectively) to recruit E2s (CDC34 and UBE2C/UBE2S, respectively).^9–11^ Because recruitment of the E2-ubiquitin thioester is crucial for RING-type E3, this interaction provides a unique attack point for pharmacological exploitation. In fact, two naturally occurring RING E3 inhibitors, Glomulin (GLMN) and early mitotic inhibitor 1 (EMI1), target E2 recruitment and inhibit SCF and APC/C, respectively.^12,13^ GLMN expressed in vascular smooth muscle cells^14^ masks the E2-binding surface on CRLs containing the RING protein RBX1, thereby preventing ubiquitin chain formation by the E2 conjugating enzyme CDC34.^12^ EMI1, in turn, targets APC/C binding to multiple surfaces to block both, substrate and E2 recruitment.^13,15^

As a key player in regulating cell cycle progression, APC/C is very often misregulated in cancer. Core subunits APC3, APC11, or its coactivator CDC20 are overexpressed in colorectal and lung adenocarcinoma, B-cell non-Hodgkin lymphomas, bladder cancer, non-small cell lung cancer, and pancreatic ductal adenocarcinoma.^16–23^ Downregulation of APC/C subunits is synthetically lethal in KRAS mutant (G13D) cell lines. Hence, reducing APC/C activity has been suggested to promote survival in Ras-driven lung cancers.^24^ Its associated E2s, UBE2C and UBE2S, are upregulated in glioblastoma, melanoma, ovary, breast, cervical, bladder, and lung cancer as well as hepatocellular carcinoma, and pancreatic and lung adenocarcinoma, and are therefore suggested as cancer prognostic markers.^25–29^ Hence, it is not surprising that APC/C is identified as a promising cancer target.^30^ Indeed, IR-tail mimetic tosyl-L-arginine methyl ester (TAME) and D-BOX receptor blocking apcin prevent CDC20 binding to APC/C and CDC20-dependent substrate recruitment, respectively.^31,32^ Despite proficiently inhibiting CDC20-dependent ubiquitinylation in cell lines, including primary patient-derived human myeloma,^31–33^ their suitability for clinical applications is unclear.

To provide a mechanistically distinct approach from TAME and apcin, we aimed to directly target the enzymatic core of the APC/C by a rationale that can be potentially extended to other RING-type E3s. To this end, we have exploited the molecular recognition aspects responsible for the binding of GLMN and EMI1 to SCF and APC/C, respectively, and we have performed an atom-detailed comparison to other RING-type E3/E2. We aimed to identify key recognition resemblances that could be exploited for structure-based *de novo* design of inhibitory RING ligands. Designing competitors of E2 recognition by RING domains and understanding the bases for their affinity and specificity could help establish a rationale to attempt inhibition of any particular RING E3.

Focusing such a pharmacophore-based strategy on APC/C has allowed the *de novo* design of inhibitory peptidomimetics more proficient than TAME or apcin in *in vitro* reconstituted activity assays. In particular, we provide a membrane-permeable hydrocarbon-stapled α-helix-based molecule able to inhibit cell division. This work provides a robust rationale extendable to future endeavors targeting RING domain-containing enzymes of therapeutic interest.

## Results

### Establishment of Principles of Inhibition on RING E3 Ligases and the Design Strategy

We conducted an atom-detailed comparative analysis of the recognition of RING-containing complex structures experimentally available at a high resolution. Inspection of the interaction of (i) five RING E3s bound to E2s and (ii) the natural inhibitors EMI1 and GLMN in complex with APC11 and RBX1, respectively,^12,13^ allowed identification of structural and physicochemical resemblances between the natural inhibitors and E2s. Furthermore, this detailed analysis was key to identifying RING residues involved in the recognition of natural inhibitors that are not participating in E2 binding and could therefore be potentially exploited in RING-ligand design strategies (i.e., incorporation of relevant chemical modifications).

Molecular dynamics (MD) simulations were used to explore RING/E2 interactions and obtain binding free energies (Methods, Table S1). This enabled the identification of interactive *hot spots* and the creation of dynamic pharmacophore models (Figure 1) for the selection of relevant physicochemical features. A common recognition motif in RING domains involves three hydrophobic residues (i.e., Trp63, Pro74, and Ile25 in APC11; Figures 1A and S1) disposed along two loops and an α-helix close to each other in three-dimensional (3D) space. Likewise, five conserved/semiconserved loop residues close in 3D constitute a common recognition motif in E2s (i.e., Arg34, Pro90, Tyr91, Ala124 and Leu125 in UBE2C; with 4–6 Å distances from side chain atoms of Ala124 to Pro90, to Tyr91, or to Leu125; Figures 1B and S1). These two structurally discontinuous motifs are the main contributors to RING/E2 recognition (Figure S2).

**Figure 1:**
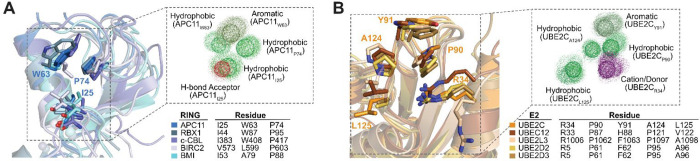
Structural and physicochemical features of RING domains and their common recognition site in E2 enzymes. (A) Structural superimposition of MD-refined structures of the RING proteins APC11 (PDB ID 5A31), RBX1 (PDB ID 1LDJ), c-CBL (PDB ID 1FBV), BIRC2 (PDB ID 6HPR), and RING1B (PDB ID 3RPG) from complexes with E2 enzymes UBE2C, UBC12 (PDB ID 4P5O), UBE2L3, UBE2D2, and UBE2D3, respectively. The respective E2-interacting residues of the RING structures are shown as sticks and colored by atom type (for the sake of clarity, only the APC11 residues are labeled). The overlay of the APC11 dynamic pharmacophore onto the structural superposition is delineated by a dotted box, and it has been displaced to the top for clarity and improved interpretability. (B) Structural superimposition of MD-refined structures of the corresponding E2 enzymes. Their respective RING-interacting residues are shown as sticks and colored by atom type (for clarity, only the UBE2C residues are labeled). The overlay of the UBE2C dynamic pharmacophore onto the structural superposition is delineated by a dotted box and it has been displaced to the top for clarity and improved interpretability. RING domains and E2 enzymes are colored in blue and orange/brown gradient, respectively. RING-coordinated zinc atoms are shown in CPK. For clarity, cartoons and zinc atoms are shown with transparency. Mesh spheres represent selected APC11 and UBE2C static pharmacophore features (aromatic, hydrophobic, H-bond acceptor, and cation/donor in smudge green, green, red, and purple, respectively) from an MD-average structure. Dots represent the dynamics of the pharmacophore features from three independent MD simulations. Figure created with PyMOL v.2.4.1.

In the analysis of APC11/EMI1 and RBX1/GLMN recognition (Figures S3–S5), their structural superposition to the E2 and analysis of their RING recognition site allowed us to establish relevant RING recognition atomic equivalences among E2s and the natural inhibitors (Scheme 1 and Figure S6). Furthermore, our analysis revealed additional contributors to RING recognition in the natural inhibitors (Scheme 1 and Figure S5). For EMI1, the most relevant interacting residues are located in an α-helix-turn (HT), whereas in GLMN they are along two α-helices.

**Scheme 1:**
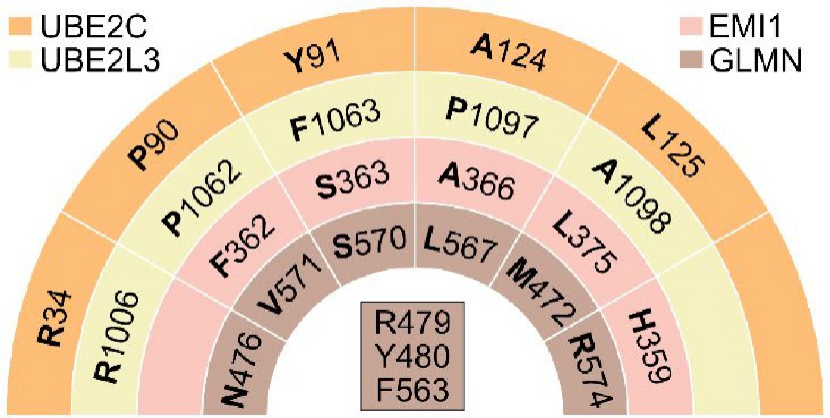
Relevant Residues of E2 Enzymes and Natural Inhibitors Recognized by RING Proteins, along with Their Spatial Equivalences upon Superposition^a^

a GLMN residues without equivalent counterparts in the other structures are boxed in black.

As a first step in our design strategy, and to achieve binding to RING proteins, the spatial distribution of common relevant functionalities was used as seeding consensus for their mapping into different nature-inspired scaffolds through a template-based rescaffolding approach^34^ accommodating the common hydrophobic functionalities from residues UBE2C_P90,Y91,A124,L125_ (correspondence with EMI1_F362,S363,A366,L375_ and GLMN_V571,S570,L567,M472_) and the hydrophilic from UBE2C_R34_ and GLMN_N476,R479,R574_ (Scheme 1). Given that the required spatial distribution of these relevant E2 functionalities is compatible with the 3D disposition of functionalities in HT or β-hairpin (BH) architectures,^35^ and that the relevant EMI1 and GLMN residues are distributed along a HT and two α-helices, we envisioned BH, HT or helix-turn-helix (HTH) (ideally 12–17 residues long) as suitable scaffolds to accommodate the required RING recognition properties. To account for structural stability, we focused particularly on disulfide-containing structures and selected three as initial cyclic scaffolds (i.e., BH (PDB ID 1KVF_SCHFGPLTWVCK(3–14)_),^36^ HT (1IMW_CRAGPLQWLCEKYFG(1–15)_),^37^ and HTH (1DVA_ALCDDPRVDRWYCQFV(1–16)_);^38^ see Methods^34^). Molecular docking of these molecules on the UBE2C recognition site of APC11 was carried out by taking into account our models and their fitting into the pharmacophores (Figure 1). Then, computer-aided structure-based *de novo* design approaches were undertaken in order to rationally enhance their binding affinity and stability (Figure 2). Optimized affinity was achieved by introducing shape and physicochemical complementarity to APC11 (i.e., chemical functionalities for improved van der Waals (vdW) and polar interactions) with the following scaffold equivalences: BH_1,12_/HT_13_/HTH_15_, BH_3_/HT_9_/HTH_2,12_, BH_6_/HT_5_/HTH_9_, BH_10_/HT_8_/HTH_11_ and HT_2_/HTH_7_ (Figure 2A,B). For stability, in addition to disulfides, residues promoting vdW in BH,^39^ as well as polar interactions in HT^37^ and HTH,^38^ were introduced at strategic positions. Besides, free N- and C-term variants were designed anticipating their experimental investigation (i.e., cellular expression). The resulting 92 molecules in complex with APC11 were MD-refined (Figure S7). The energetically best eight were selected for detailed analysis (i.e., binding free energy (Figure 2B), per-residue/pairwise contributions (Table S2 and Figure S8), and contacts (Figure S9; see Methods). BH1, BH2, BH3, HTH1, and HTH2 showed the most favorable binding energies (Figure 2B) and were predicted to interact with APC11 residues Ile25, Arg27, Met28, Pro35, Asp36, Lys62, Trp63, Gln67, Pro74, and Arg77. These residues are relevant for UBE2C recognition. Therefore, our designs could potentially compete with UBE2C for binding to APC11 (Figures 2C, S8, S9 and Table S2). In BH and HT, per-residue energy analysis indicated Ile25, Arg27, and Trp63 as the most contributing. For HTH, Pro35 and Asp36 showed relevant contributions (Table S2). Given that our designs incorporated recognition features from GLMN (inhibitor of APC11 closest homologue RBX1 (37% and 59% sequence identity and similarity, respectively)), we investigated their recognition by RBX1 at the E2 site. We also explored the molecular basis accounting for selectivity by focusing on the conserved and semiconserved residue correspondences in the superimposed APC11 (PDB ID 4UI9) and RBX1 (PDB ID 1LDJ) structures at the tackled interface: APC11_I25_/RBX1_I44_, APC11_R27_/RBX1_R46_, APC11_M28_/RBX1_N47_, APC11_P35_/RBX1_I54_, APC11_D36_/RBX1_E55_, APC11_K62_/RBX1_R86_, APC11_W63_/RBX1_W87_, APC11_Q67_/RBX1_R91_, APC11_P74_/RBX1_P95_ APC11_M75_/RBX1_L96_, and APC11_R77_/RBX1_N98_ (Figure S1A,B). The physicochemical variation observed in APC11_M28_/RBX1_N47_, APC11_Q67_/RBX1_R91_, and APC11_R77_/RBX1_N98_ (i.e., hydrophobic and charged versus neutral and length variation indicating different flexibility) could be ideally exploited for introducing selectivity. Indeed, these three APC11 residues were predicted to interact with our molecules (Figure S8). BH, HT, and HTH manually docked to RBX1 were MD-refined, and the obtained binding energies were in the same range as for APC11 (Table S3 and Figure 2B). Pairwise energetic contribution analysis indicated a similar recognition pattern to APC11 (i.e., RBX1 Ile44, Arg46, Glu55, Trp87, Pro95, Leu96, Asn98, Figure S10), predicting low selectivity between both RINGs. Based on these results (i.e., favorable interaction energies and mimicry of RING recognition by E2/natural inhibitors), these eight *de novo* designed E2 mimetics (Figure 2B) were proposed for synthesis and experimental evaluation.

**Figure 2:**
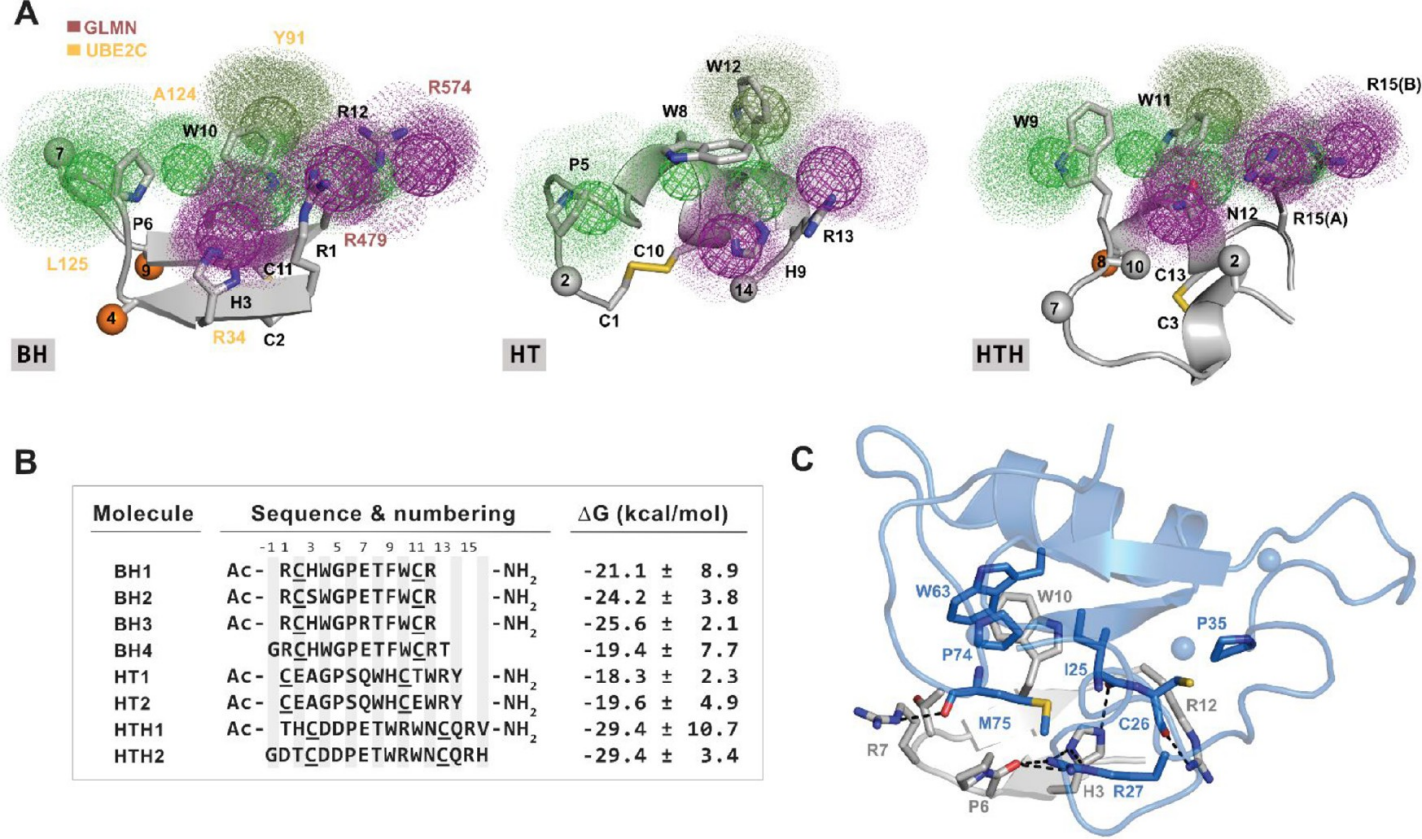
Scaffold design strategy of *de novo* E2 mimetics. (A) Three protein-like cyclic scaffolds (i.e., containing disulfide bonds; in yellow sticks) are shown in gray cartoons: β-hairpin (BH), helix-turn (HT) and helix-turn-helix (HTH) (coordinates taken from PDB ID 1KVF, 1IMW, and 1DVA, respectively). These structures were used to fit dynamic pharmacophore features from selected residues from UBE2C and GLMN. Mesh spheres represent aromatic (smudge green), hydrophobic (green) and cation/donor (purple) static pharmacophore features from an MD-average structure. Dots represent the dynamics of the pharmacophore features from three independent MD simulations. The side chains of scaffold residues fitting the pharmacophore features are shown in sticks, colored by atom type and labeled. Scaffold positions used to introduce additional stabilization and interactions with APC11 are numbered and indicated by orange and gray spheres, respectively. In the HTH scaffold, residue R15 could adopt two possible rotamers fitting the pharmacophore (labeled as R15­(A) and R15­(B)). (B) Best designed candidates based on their calculated binding free energies and their respective sequences. Underlined cysteine residues denote disulfide bridges. (C) MD-refined structure of BH3 (gray) in complex with APC11 (blue). Snapshot at 38.5 ns. Interacting residues are shown in sticks, colored by atom type and labeled. Zinc atoms are shown as spheres. H-bonds are depicted with black dashed lines. Figure created with PyMOL v.2.4.1.

### Experimental Validation of E2 Mimetics

To assess prevention of RING activation by our E2 mimetics, and thus validate our design strategy, we employed *in vitro* APC/C ubiquitylation assays containing immunoprecipitated APC/C from metaphase-synchronized HeLa cells, recombinant UBA1 (E1), UBE2C (E2), ubiquitin, and dye-labeled metaphase APC/C substrate securin (Figure 3A). Quantitative in-gel scanning of end-point assays revealed that 100 μM BH1–3 and HTH significantly reduced securin ubiquitylation compared to control reactions, whereas BH4 and both HT showed no such effect (Figure 3B,C). Comparable results were obtained in APC/C assays using the N-terminus of cyclin B1 (residues 1–86) as an alternative canonical APC/C substrate (Figure S11A,B). The best BH and HTH molecules also inhibited ubiquitylation of securin when using the E3-promiscuous E2 enzyme UBE2D1 (UbcH5), which interacts with APC11 in the same manner as UBE2C (Figures 1B and S11C). Together, this indicated that the inhibitory effect shown by our *de novo* designed molecules (named *^i^* APC11), which target the APC11 RING protein, is neither substrate- nor E2-specific. Time courses with the two best *^i^* APC11, BH3, and HTH1, showed that maximal inhibition was reached already after 15 min (Figure 3D). The interaction of UBE2C with APC/C is highly transient and only revealed in the presence of cross-linkers.^40^ Because we designed *^i^* APC11 molecules to competitively inhibit APC/C by binding to the RING domain, we tested the ability of bead-bound BH3 to precipitate APC11 from cell extracts. To immobilize BH3 to beads, we used our models to introduce propargylglycine (Pra) (Pra-BH3) at the N-term and thus enable side chain conjugation with azide agarose beads by click chemistry (Figure 3E). Indeed, agarose pulldowns with extracts prepared from asynchronously growing HeLa cells showed strong enrichment of APC11 on Pra-BH3 beads but not of the homologous RING protein RBX1 (Figure 3F). Pra-BH3 also precipitated other RING-type E3 including BRCA1, BMI1, MDM2, c-CBL and c-IAP1 (Figure 3F). However, except for c-CBL and MDM2, those interactions were weaker, as judged by the degree of enrichment over the input of the assay (Figure 3G).

**Figure 3:**
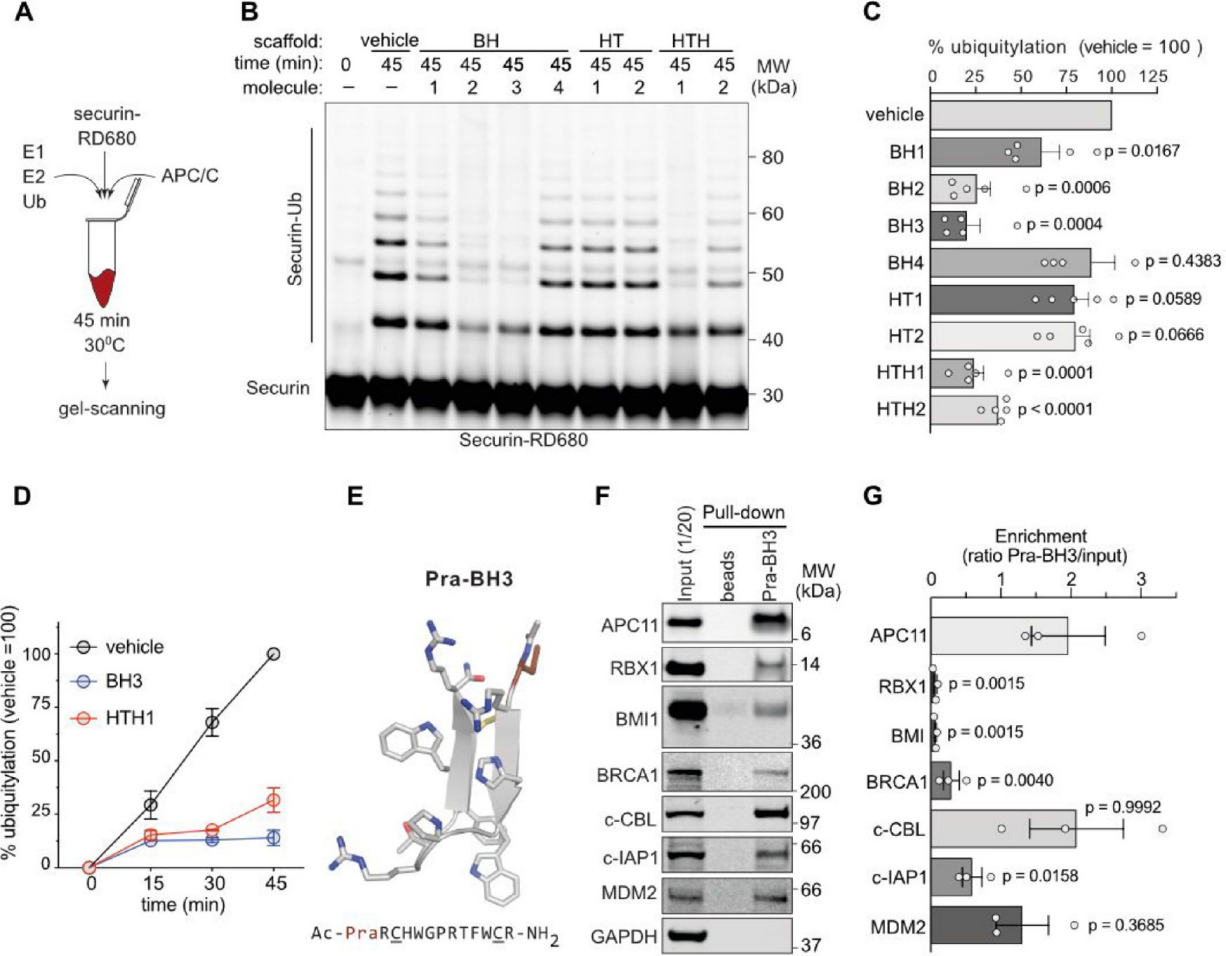
E2 mimetics bind and inhibit APC/C *in vitro* **.** (A) Illustration of *in vitro* APC/C activity assay based on immunopurified metaphase APC/C and recombinant E1, E2, methylated ubiquitin (Ub), ATP and IR-dye RD680 labeled substrate securin. (B) In-gel scan showing the covalent linkage of Ub to securin during *in vitro* APC/C activity assays as in (A) in the presence of 100 μM E2 mimetics. (C) Quantification of (B). Bars showing the mean ± SEM. Significance according to one-sided *t* test compared to vehicle = 100, *n* = 5. (D) Time course of securin ubiquitylation with the best BH and HTH molecule from (C,D). Circles indicate the mean ± SEM, *n* = 3. (E) Molecular model of Pra-BH3. The Pra group used for click chemistry is highlighted as a brown stick. Figure created with PyMOL v.2.4.1. (F) Western blot analysis detecting RING containing proteins in pull-down experiments with metaphase extracts performed with Pra-BH3 clicked to azide agarose beads. The highly abundant RING-less protein GAPDH serves as a negative control for nonspecific binding. (G) Quantifications of (F). Bars represent the mean ratio of enrichment ± SEM of binding to Pra-BH3 compared to the input sample. Note, only APC11, c-CBL and to a lesser degree MDM2 RINGs are enriched on beads. Significance according to 1-sided *t* test compared to APC11, *n* = 3.

In summary, our *de novo*-designed *^i^* APC11 molecules bind RING E3s and inhibit APC/C activity *in vitro*.

### Enhancement of APC/C Recognition and Stability Properties for Delivery

In order to improve affinity and selectivity toward APC11, we explored the use of α-helix mimetics as a helical scaffold (H), enabling a spatial distribution of functionalities similar to BH,^35^ our best *^i^* APC11 scaffold so far. This design strategy could potentially facilitate the introduction of other required modifications for different purposes (*vide infra*).

First, we further examined the structure of RBX1/GLMN (PDB ID 4F52)^12^ and compared it to EMI1/APC11 (PDB ID 4UI9)^13^ (Figures S3–S5), identifying two additional recognition spots (GLMN_F563_ and GLMN_N476_ (i.e., aromatic and H-bond acceptor, respectively); Figures S4 and S6) that could be exploited for our designs and thus expand our previous pharmacophore model (Figure 4A). Introducing aromatic features representing GLMN_F563_ could facilitate interactions with APC11_R77_ against RBX1_N98_, enhancing affinity and also selectivity toward APC11 (Figure S1A,B). Nevertheless, introducing H-bond acceptor features could also facilitate APC11_R77_ recognition as predicted for BH, HT, and HTH (Figure S8). Besides, GLMN_N476_ acts as an H-bond donor, like UBE2C_R34_, and also bears an H-bond acceptor that might stabilize the observed GLMN_R479_ rotamer when interacting with RBX1_E55_ (Figure S4A) and could interact with APC11_R27_, as particularly predicted for HTH1 (Figures S8 and S9), thereby strengthening APC11 recognition.

**Figure 4:**
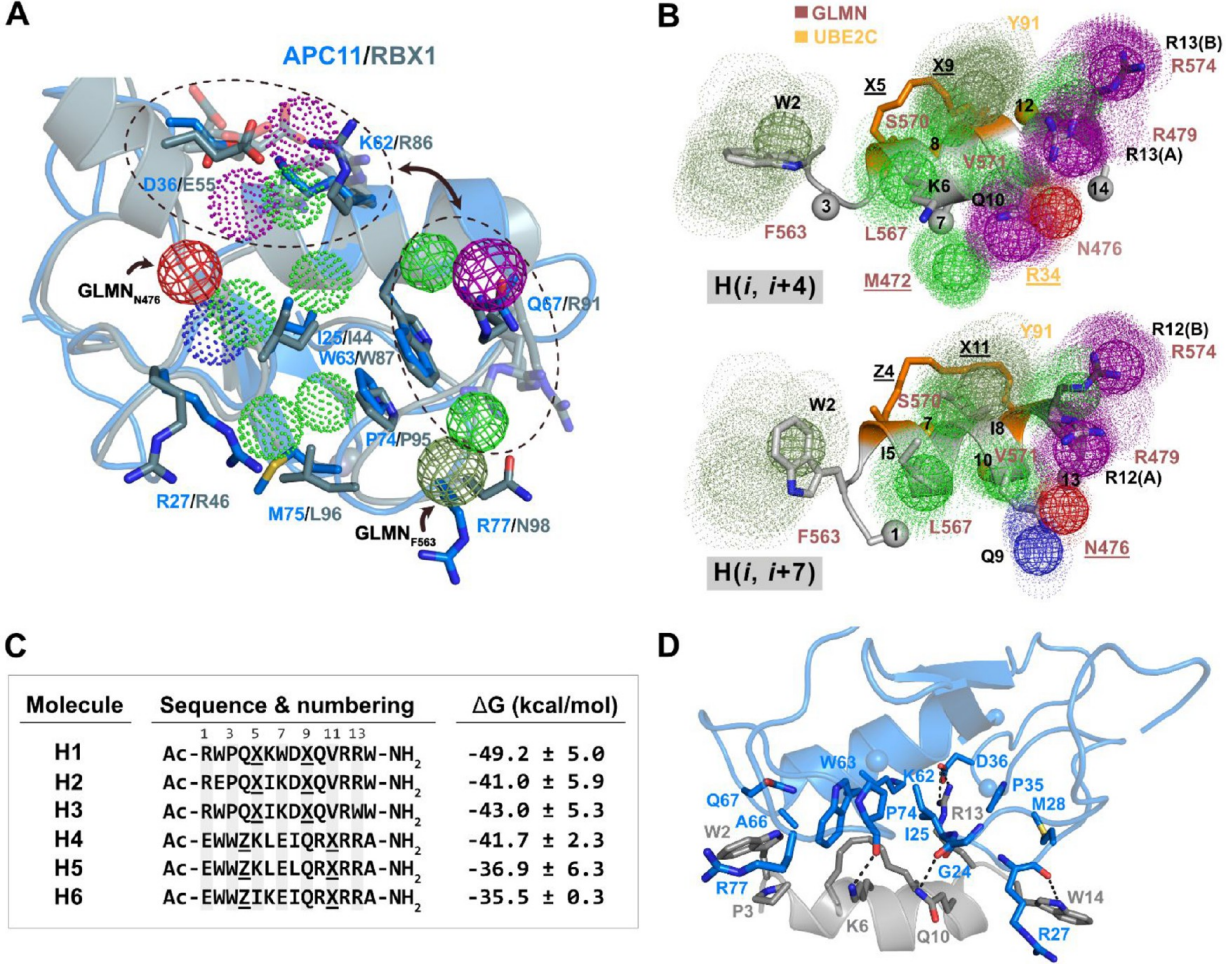
New scaffold design enhancing APC/C recognition. (A) Design strategy for APC11 selectivity. Superimposition of the crystallographic structures of APC11 (PDB ID 4UI9, 3.6 Å, blue) and RBX1 (PDB ID 4F52, 3.0 Å, gray blue). Different rotamers for residues RBX1_E55_ and RBX1_R91_, taken from three other available RBX1 structures (PDB IDs 1LDJ, 3.0 Å, 4P5O, 3.1 Å, and 2HYE, 3.1 Å) are shown as sticks and in transparency. Conserved and semiconserved residues at the E2 interface are shown in sticks, colored by atom type and labeled. Previously considered and expanded pharmacophore features are shown as dotted and mesh spheres, respectively (aromatic in smudge green, hydrophobic in green, H-bond acceptor in red, H-bond donor in blue, and cation/donor in purple). The two regions (marked by discontinuous lines) and two particular residues from GLMN used in our design strategy for enhancing selectivity and binding are indicated by arrows. (B) Two α-helical scaffolds, H­(*i*, *i*+4) and H­(*i*, *i*+7), containing a hydrocarbon staple (in orange sticks; labeled and underlined) are shown in gray cartoons with an overlay of the MD-based pharmacophore features from GLMN and UBE2C. Their respective residues used to derive the pharmacophore are labeled in brown and light orange. The underlined GLMN and UBE2C labels highlight the differing pharmacophore features between the two scaffolds. Mesh spheres represent selected pharmacophore features (aromatic in smudge green, hydrophobic in green, H-bond acceptor in red, H-bond donor in blue, and cation/donor in purple) from MD-average structures. Dots represent the dynamics of the pharmacophore features along three independent MD simulations. Residues overlapping with dynamic pharmacophore features are shown in sticks, colored by atom type, and labeled. Scaffold positions used to introduce additional stabilization and interactions with APC11 are numbered and indicated by orange and gray spheres, respectively. The arginine residue at position 13 of scaffold H­(*i*, *i*+4) and position 12 of H­(*i*, *i*+7) can adopt two possible rotamers (respectively labeled as (A) and (B)) fitting the cationic pharmacophore features. (C) Sequence and calculated binding free energies of the designed *^i^* APC11 molecules. X refers to (*S*)-2-(4′-pentenyl)­Ala, Z refers to (*R*)-2-(7′-octenyl)­Ala and underlined Z and X denote a hydrocarbon-staple. (D) MD-refined structure of H1 (gray) in complex with APC11 (blue) (snapshot at 39.9 ns). Interacting residues are shown in sticks, colored by atom type and labeled. Zinc atoms are shown as spheres. For clarity, cartoon representations and zinc atoms are shown with transparency. H-bonds are depicted with black dashed lines. Figure created with PyMOL v.2.4.1.

To enhance selectivity, we focused on (i) the distinct recognition environment observed for the superimposed structures of APC11 and RBX1 involving residues APC11_Q67_ and RBX1_R91_ (PDB ID 4UI9)^13^ and (ii) the subtle differences in the 3D arrangement of APC11_D36_ and APC11_K62_ with respect to the equivalent RBX1_E55_ and RBX1_R86_ (PDB ID 4F52, 4P5O, 1LDJ, 2HYE)^12,41–43^ (Figure 4A). In the helical scaffold docked at the E2 binding site, the introduction of a hydrophobic moiety or a positive charge near the guanidinium of RBX1_R91_ would promote repulsion toward RBX1, potentially enhancing selectivity for APC11. Conversely, including a negative charge could facilitate interactions with both proteins (Figure S12). This strategy can be leveraged as a tandem effect by using the different 3D dispositions of the interacting APC11_D36_ and APC11_K62_ versus their equivalent RBX1_E55_ and RBX1_R86_ (Figure 4A). Therefore, as anticipated by the previous designs, having a positive charge promoting interactions with APC11_D36_ (i.e., mimicking GLMN_R479,R574_ recognition by RBX1_E55_) could aid in displacing a helical scaffold from the E2 recognition site of RBX1, thereby avoiding repulsion with the positive charge of RBX1_R86_. Hydrocarbon staple helical mimetics have shown good serum stability and permeability properties,^44^ overcoming disulfide cellular reduction. Therefore, to ensure delivery and structural stability, we considered incorporating a hydrocarbon staple into the helical scaffold, which could also add interactions to APC11 at the E2 recognition site. Furthermore, the introduction of positively charged amino acids can enhance cellular uptake.^45^ Besides, through an α-helix mimetic, we could facilitate the implementation of additional delivery strategies if needed.

Based on the above-exposed, we considered two hydrocarbon stapled α-helix mimetics of 14–15 residues (H­(*i*, *i* + 4) (PDB ID 4DJS)^45^ and H­(*i*, *i* + 7) (PDB ID 4N5T)),^46^ see Methods, Figure S12). We incorporated the eight features from previous designs (Figure 2A), the two additionally selected from GLMN, and enlarged hydrophobicity features from UBE2C_Y91_ and GLMN_S570_ (Figure 4A,B). Furthermore, we explored various substitutions to obtain −1 to +4 net charges. Considering the spatial correspondences of selected functionalities from previous BH and HTH designs (Table 1, Figures 2A,B and 4B) and the differences at the APC11 and RBX1 recognition regions, 50 new α-helix mimetics were designed and MD-refined in complex with APC11. For H­(*i*, *i* + 4), properties for introducing affinity and selectivity were explored at positions 1–3, 5–7, 9, 10, and 12–14, while scaffold stability was introduced at 5, 8, 9, and 12. For H­(*i*, *i* + 7), positions 1, 2, 4, 5, 8, 9, 11–13 were exploited for introducing affinity and selectivity, and positions 4, 7, 10, and 11 were exploited for scaffold stability (Figures 4B and S12).

**Table 1:** Position Equivalences of Selected Functional Properties upon the Superimposition of Scaffold designs

	Scaffold spatial equivalences							
BH	1, 12	3	6	7		8	10	
HTH	15	2, 12	9		7		11	
H(*i*, *i* + 4)	13	10	6	2, 3		5	9	14
H(*i*, *i* + 7)	12	9	5	2	1	4	8	13

Based on the obtained interaction energies and contacts, six molecules (i.e., H1–3 (*i*, *i* + 4) and H4–6 (*i*, *i* + 7)) were selected for detailed interaction analysis with APC11 as was done for the E2 mimetics (Figure 4C, Table S4, Figures S13 and S14; see Methods). A remarkable binding improvement was observed with respect to BH, HT, and HTH (Figures 2B and 4C). The obtained pairwise and per-residue energy contributions confirmed the efficient recognition of H molecules by APC11_I25_, APC11_R27_, APC11_M28_, APC11_P35_, APC11_D36_, APC11_K62_, APC11_W63_, APC11_A66_, APC11_Q67_, APC11_P74_ and APC11_R77_ (Figures S13, S14 and 4D), which resemble the critical residues for UBE2C/APC11 interaction and include the crucial Met28, Gln67 and Arg77 for selectivity against RBX1 (Figures 4A and S1A,B). Furthermore, we observed contacts of H_2_ (i.e., Trp aromatic or Glu H-bond acceptor properties) with APC11_R77_ and H­(*i*, *i* + 4)_10_/H­(*i*, *i* + 7)_9_ (i.e., Gln *H*-bond donor and acceptor properties) with APC11_I25,R27_ (Figure S13), confirming our rationale to enhance APC11 recognition and selectivity. Notably, the calculated binding energies of H molecules toward RBX1 clearly showed a weaker recognition than to APC11 (Table S5). In particular, for H1, H3, H4, or H6, no interactions were observed with RBX1_R91_, RBX1_P95_, or RBX1_N98_, while only for H2 and H5 weak interactions were predicted (Figure S15). These observations clearly reflect a partial displacement of the H molecules from the E2 recognition site, which further supports our design strategy for APC11 selectivity. In addition, the H designs showed a reduced energetic strength and number of contacts toward RBX1_I44_, RBX1_R46_, and RBX1_W87_ (Figure S15) in comparison to APC11_I25_, APC11_R27_, and APC11_W63_ (Figure S13), which suggests a much lower efficiency to prevent RBX1/UBC12 recognition than APC11/UBE2C. Altogether, the *de novo* designed H1–6 were predicted to be selective APC11 inhibitors (*^i^* APC11) and were proposed for synthesis and experimental validation.

### Experimental Evaluation of *^i^* APC11 Molecules

To investigate the predicted improved binding and selectivity of H1-6 toward APC11, we first assessed their ability to precipitate APC11 and RBX1 from cell extracts, as previously done with BH3 (Figure 3). To this end, we used our molecular models to identify a plausible position (i.e., the solvent-exposed Gln4) in which a Pra functionality could be introduced for click chemistry to link our best H molecule to azide agarose beads (Pra-H1) for pull-down experiments, as previously done for Pra-BH3 (Figure 5A). Indeed, we observed a 2-fold increase in APC11 precipitated by Pra-H1 compared to Pra-BH3. Binding to RBX1 remained very weak and unchanged for both molecules. In contrast, Pra-H1 binding to c-CBL and MDM2 became ∼2- and 4-fold reduced, respectively (Figure 5B,C). While we cannot exclude that *^i^* APC11 might additionally bind to other protein regions, the reduction in c-CBL and MDM2 binding indicates that our strategy to enhance APC11 binding and selectivity, at least toward the tested RING proteins, was successful.

**Figure 5:**
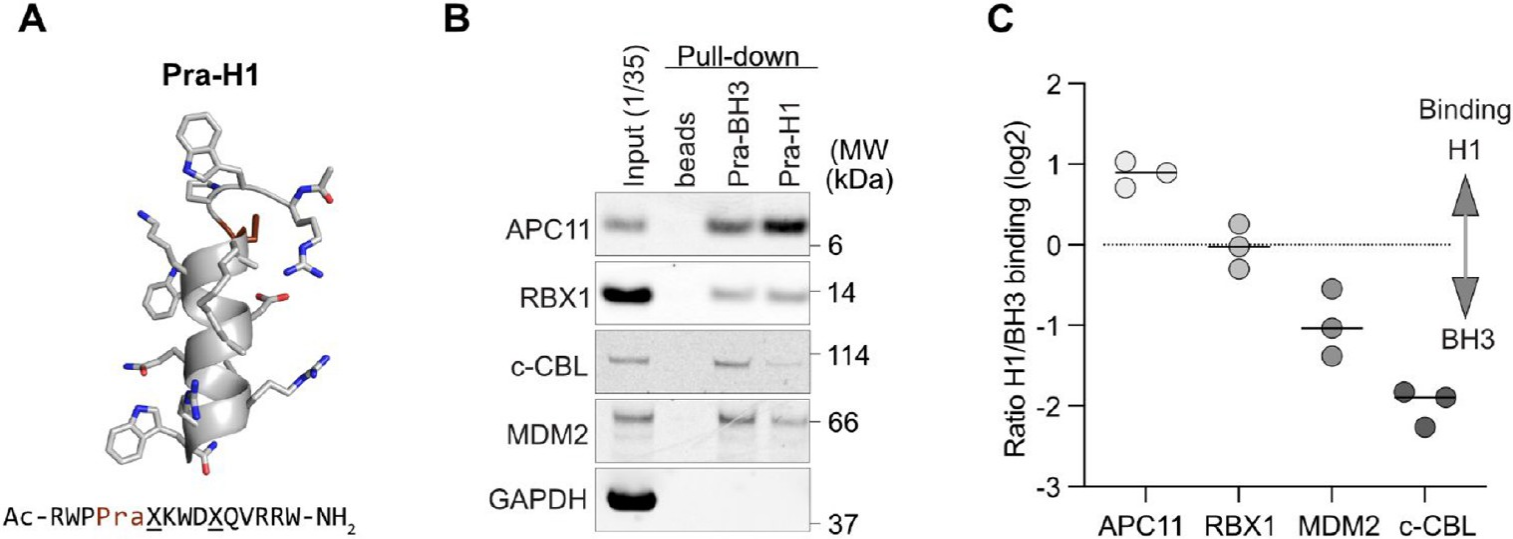
Improved binding and specificity of ^*i*^APC11. (A) Molecular model of Pra-H1. The Pra group used for click chemistry is highlighted as a brown stick. Figure created with PyMOL v.2.4.1. (B) Western blot analysis of pull-down experiments with metaphase extracts comparing the ability of Pra-BH3 and Pra-H1 clicked to azide agarose beads to bind the RING domain containing proteins. The highly abundant RING-less protein GAPDH serves as a negative control for nonspecific binding. (C) Quantification of (B). Lines show the mean log2 ratio of RING protein binding of H1 over BH3. Rational optimization of H1 resulted in an increased level of APC11 but decreased levels of c-CBL and MDM2 binding, *n* = 3.

### Enhanced APC/C Inhibition by *^i^* APC11 *In Vitro* Compared to Commercially Available Inhibitors

Next, we tested the APC/C inhibitory proficiency of H1-6 in fully reconstituted activity assays with recombinant APC/C in comparison to BH3 and the commercially available APC/C inhibitor TAME.^32^ To account for mechanistic differences in APC/C inhibition between TAME (inhibits CDC20 binding) and *^i^* APC11 (inhibits E2 binding), all inhibitors, E2s and CDC20 were added simultaneously to APC/C. Remarkably, *in vitro* ubiquitination assays with APC/C substrate securin showed that H1 and H3-6 exhibited a much better inhibitory effect on APC/C activity than TAME at the same concentration (100 μM). In contrast, a negative control (Ng, Ac-RRKZAQERARXVRY-NH_2_, Z refers to (*R*)-2-(7′-octenyl)­Ala, X refers to (*S*)-2-(4′-pentenyl)­Ala, and underlined Z and X denote a hydrocarbon-staple) designed to not interfere APC11 recognition by the E2 was not inhibitory (Figure 6A,B). *^i^* APC11 molecules also efficiently inhibited ubiquitylation of a ubiquitin-cyclin B1 fusion (Ub-cyclin B1) independently of whether UBE2C, UBE2D1 or UBE2S were used as the E2 enzyme (Figure S16A). The observation that H6 completely prevented UBE2S-driven chain elongation with and without the APC/C (Figure S16A) may indicate direct inhibition of UBE2S or allosteric inhibition of UBE2S recruitment because the UBE2S interaction surface with APC/C is distinct from UBE2C and UBE2D1.^13,47,48^ To exclude that H6 indirectly inhibits APC/C activity, i.e., by interfering with E2-ubiquitin thioester formation or ubiquitin-lysine linkage, we measured UBE2C and UBE2S autoubiquitination *in vitro* in the presence of 100 μM H6. We observed no difference for UBE2C and only a small (∼10%) decrease in UBE2S autoubiquitylation compared to control reactions (Figure S16B–D).

**Figure 6:**
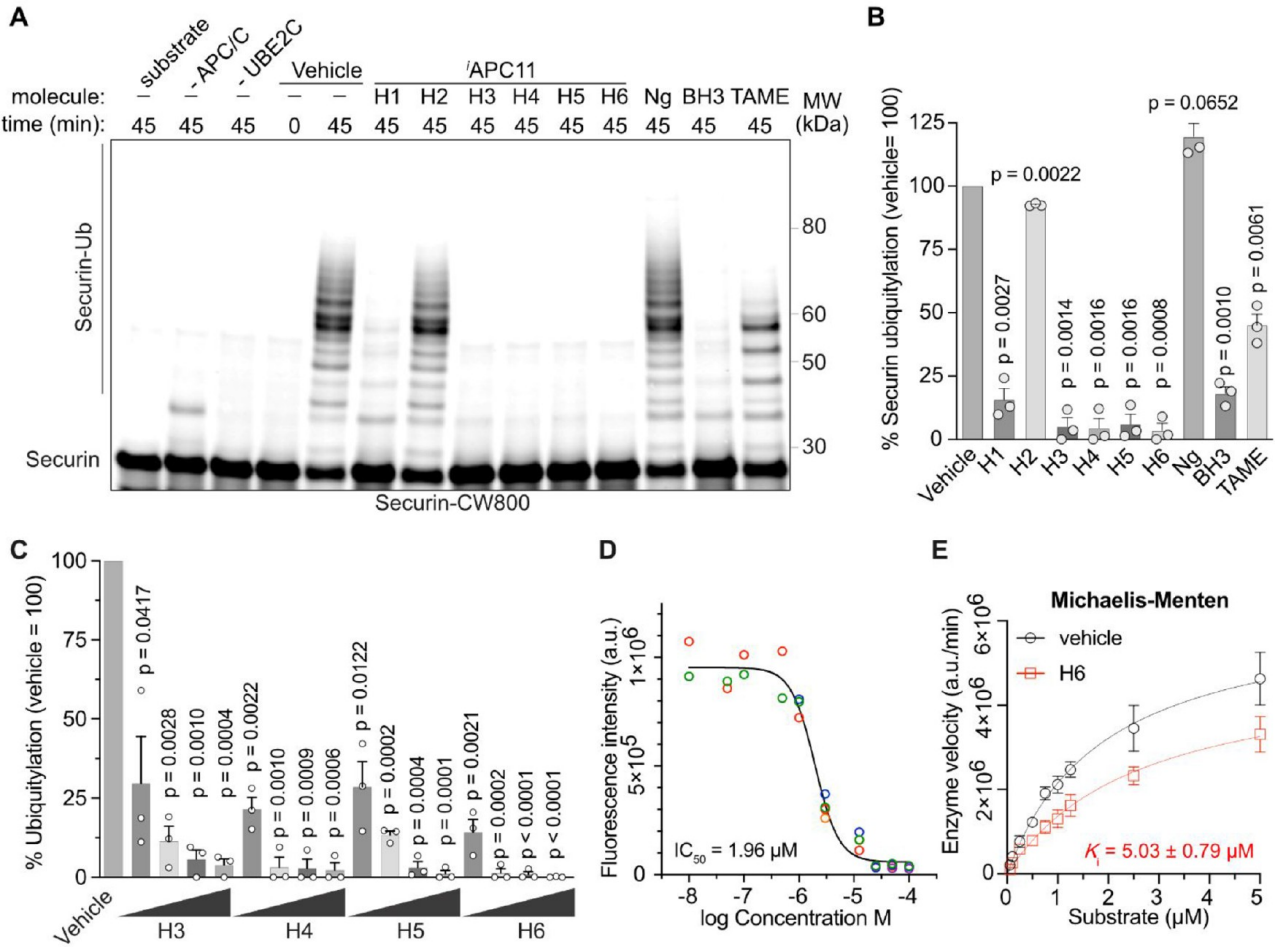
^*i*^APC11 efficiently inhibits APC/C. (A) In-gel scan showing the covalent linkage of Ub to IRdye-labeled securin during *in vitro* APC/C activity assays as the presence of 100 μM ^*i*^APC11 molecules compared to 100 μM BH3 and 100 μM commercial APC/C inhibitor TAME. (B) Quantification of (A). Bars showing the mean ± SEM. Significance according to 1-sided *t* test compared to vehicle = 100, *n* = 3. (C) Quantification of *in vitro* APC/C activity assays titrating ^*i*^APC11 molecules from 25, 50, 75, and 100 μM as shown in Figure S17A. Bars showing the mean ± SEM. Significance according to 1-sided *t* test compared to vehicle = 100, *n* = 3. (D) IC_50_ determination by nonlinear curve fitting derived from *in vitro* APC/C activity assays shown in Figure S17B. Data points from independent experiments are indicated by different colored circles, *n* = 4. (E) Michaelis–Menten graph and *K*_i_ determination from *in vitro* APC/C activity assays shown in Figure S17C, *n* = 3. Note, *V*_max_ values of vehicle and H6 reactions do not converge, indicative of noncompetitive inhibition.

Because H3-6 almost completely inhibited securin ubiquitylation, we titrated their concentration to identify the best molecule for further evaluation and refinement (Figure S17A). Here, H6 performed best and reduced APC/C activity toward securin at the lowest concentration of 25 μM by more than 80% (Figure 6C). Together, this demonstrates that H6 inhibits APC/C more strongly than TAME *in vitro* and acts independently of the substrate and E2, as predicted by our theoretical models.

To quantify the inhibitory effectiveness of H6, its IC_50_ was determined.^49^ To this end, we performed *in vitro* APC/C ubiquitination assays in the presence of H6 titrated from 0.01 to 100 μM (Figure S17B). Nonlinear curve fitting of fluorescence intensities of the ubiquitylated substrate for each concentration revealed an IC_50_ of 1.96 μM (Figure 6D). Based on our models, the *^i^* APC11 molecules should block E2 recruitment to the RING domain of APC/C and thus noncompetitively inhibit substrate ubiquitination (Figures 2 and 4). To distinguish between competitive and noncompetitive inhibition, we determined *K*_m_ and *V*_max_ using *in vitro* APC/C ubiquitination assays under initial velocity conditions (i.e., when less than 10% of the substrate is modified). We titrated the substrate securin from 50 nM to 5 μM and tested the APC/C activity in the presence and absence of H6 (Figure S17C). We found that *K*_m_ was not significantly affected by H6; in contrast, *V*_max_ was reduced (Figure S17D,E). This is consistent with H6 acting as a noncompetitive inhibitor, as predicted, and further revealed a *K*_i_ of 5.03 ± 0.79 μM (Figure 6E). Notably, the *K*_i_ of apcin determined by the same methodology was 23 μM and thus ∼4.5 higher.^31^ We conclude that RING-targeting *^i^* APC11 molecules inhibit APC/C stronger than the IR-tail mimetic TAME *in vitro* and have a lower *K*_i_ than the D-box inhibitor apcin.

### Structure-Based Rationale for a Membrane Permeant *^i^* APC11 Molecule Prolonging Mitosis in Human Cancer Cells

Efficient inhibition of APC/C activity should arrest cells in mitosis, specifically in metaphase, because ubiquitylation and subsequent degradation of cyclin B1 and securin are essential to initiate anaphase and thus chromosome segregation.^7^ We first assessed if unmodified *^i^* APC11 molecules that inhibited the APC/C *in vitro* (Figure 6) prolonged mitosis in nontransformed human retina pigment epithelial cells (RPE-1). To this end, we determined the length of mitosis by measuring the duration from nuclear envelope breakdown to anaphase by bright field microscopy between 10 and 24 h after treatment with 50 μM inhibitors. We did not observe significant differences in mitotic timing compared to that of vehicle-treated RPE-1 cells (Figure S18A). Clicking the fluorophore Cy5 to Pra-H1 suggested that the lack of inhibition was likely because the majority of *^i^* APC11 accumulated in a punctate-like pattern reminiscent of endosomal vesicles (Figure S18B), whereas the APC/C is predominantly localized to the cytoplasm and nucleus.^50^

We therefore used our theoretical models to check where to conjugate *^i^* APC11 to the endosomal escape peptide GFWFG (EE).^51^ The comparison of H6 docked to APC/C (PDB ID 4UI9)^13^ to the modeled APC11/H6 complex suggested an N-term versus C-term elongation due to clashes with the APC1 subunit. To further enhance endosomal escape and deliverability, we decided to link EE and H6 N-term with polyethylene glycol (PEG)^51^ to obtain the molecule Ac-GFWFG-(PEG)_6_-EWPraZIKEIQRXRRA-NH_2_ (Pra-_EE1_H6), which bears the unnatural amino acid Pra to facilitate the assessment of localization in cells (as stated above, Z refers to (*R*)-2-(7′-octenyl)­Ala, X refers to (*S*)-2-(4′-pentenyl)­Ala, and underlined Z and X denote a hydrocarbon-staple).

We first tested if adding EE and PEG-linker would improve localization of Pra-_EE1_H6 compared to that of Pra-H1 and Pra-H6 (Ac-EWPraZIKEIQRXRRA-NH_2_), this time by using human cervical cancer cells (HeLa) as a cellular model. As observed before in RPE-1 cells (Figure S18B), Pra-H1 and Pra-H6 localized in a punctate pattern that partially overlapped with the early endosomal marker Rab5 (Figure 7A,B). In contrast, adding EE and PEG_6_ resulted in increased cytoplasmic and nucleoplasmic localization of Pra-_EE1_H6 (Figure 7C). We noted that Pra-_EE1_H6 accumulated in the nucleus earlier than Pra-H6 (Figure S18C), indicative of superior uptake or EE kinetics. Next, we added vehicle or 50 μM Pra-_EE1_H6 to HeLa cells and monitored the length of mitosis by live cell imaging between 10 and 48 h after adding the inhibitors. Pra-_EE1_H6 treatment significantly prolonged mitosis by an average of ∼74 min, though the median change remained small, suggesting that not all cells responded equally (Figure S18D). One plausible explanation for the only mild mitotic delay despite the nuclear and cytoplasmic localization of the _EE1_H6 molecule is the PEG linker length, which could bring EE packing into the hydrophobic patch of H6 required for APC11 recognition. Supporting this idea, Pra-_EE1_H6 immobilized on azide agarose beads precipitated less APC2 and APC11 compared with Pra-H1 and Pra-H6 (Figure S18E). To overcome the potential hindrance of Pra-_EE1_H6 recognition by APC/C due to the linker length, the new derivative Ac-GFWFG-NH­(CH_2_)_2_OCH_2_CO-EWWZIKEIQRXRRA-NH_2_ (_EE2_H6) was designed with a shorter linker.

**Figure 7:**
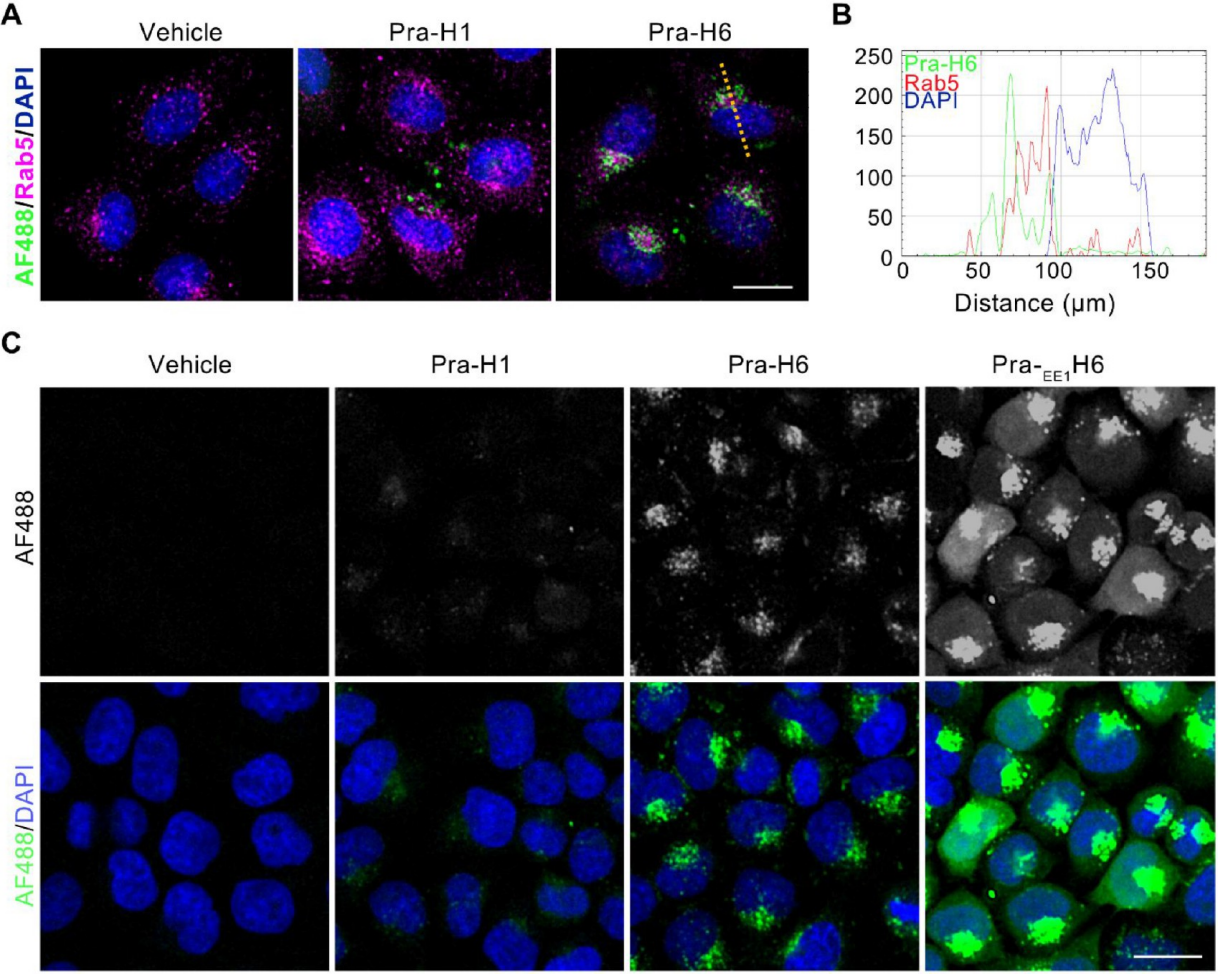
Improved cellular localization of ^*i*^APC11 through endosomal escape sequence. (A) Micrographs showing the localization of *^i^* APC11 compounds in HeLa cells. Cells were treated with 50 μM of Pra-H1, Pra-H6, or vehicle for 10 h before they were fixed and clicked to the dye AF488. (B) Line scan from a representative cell in panel A (yellow dotted line) indicating a partial overlap of Pra-H6 staining with the early endosome marker Rab5. (C) Localization of Pra-H1, Pra-H6, and Pra-_EE1_H6. Note, the addition of an endosomal escape sequence enables Pra-_EE1_H6 to be released into the cytoplasm and nucleus. Scale bars are 20 μm.

Next, we assessed whether _EE2_H6 *^i^* APC11 causes a mitotic arrest in metaphase, indicative of APC/C inhibition. Indeed, adding 50 μM of _EE2_H6 to HeLa cells strongly prolonged metaphase compared to vehicle-treated cells (Figure S19A,B), resulting in mitoses of an average length of almost 2 h (Figure 8A). Analyzing the mitotic fate of cells in response to _EE2_H6 showed that 75% of cells displayed prolonged mitosis, and in almost 20% of such cells, this led to mitotic cell death (Figure 8B). We confirmed the mitotic delay caused by _EE2_H6 treatment in lung carcinoma (A549), fibrosarcoma (HT-1080), and colon carcinoma (RKO) cell lines (Figure 8A), but noted that the triple-negative breast cancer (MDA-MB-231) and colorectal (SW480) cancer cells remained unaffected (Figure S19C). Like HeLa cells, 20% of HT-1080 cells died in mitosis in response to *^i^* APC11 treatment, whereas A549 and RKO cells displayed only a prolonged mitosis phenotype (Figure 8B). Therefore, as judged by the prolonged time cells spend in the metaphase, membrane-permeant _EE2_H6 targets APC/C activity and results in increased postmitotic cell death in a subset of cancer cell lines.

**Figure 8:**
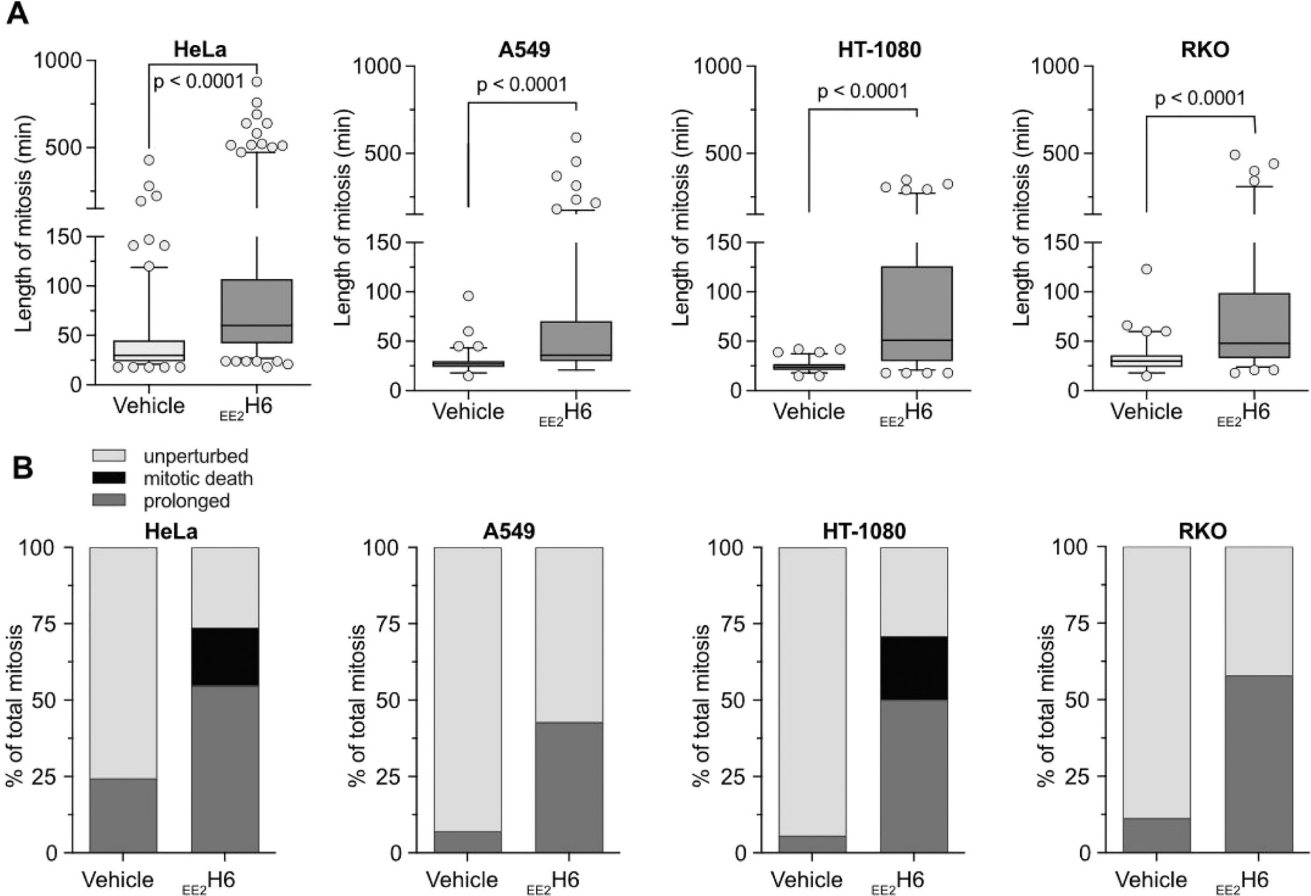
^*i*^APC11 causes mitotic arrest and cell death in cancer cells. (A) Boxplots indicating the length of mitosis from nuclear envelope breakdown to anaphase in different cancer cell lines determined by live cell microscopy from single cells between 10 and 24 h of treatment with 50 μM _EE2_H6 *^i^* APC11. Box shows the median, 25^th^, 75^th^ percentiles, and whiskers the 5–95 percentiles. Significance according to two-tailed Mann–Whitney test, *n* = 3 (HeLa), *n* = 2 (other cell lines). (B) Mitotic cell fate analyses of the cells analyzed in (A) classifying mitosis into categories “unperturbed”, “prolonged”, and “mitotic death” (see Methods). Note, two cancer cell lines that do not respond to _EE2_H6 treatment are presented in Figure S19C.

## Discussion and Conclusions

RING-type E3 ligases are involved in ubiquitination, regulating protein degradation, and controlling numerous cellular processes. Their dysregulation can lead to cancer and other pathologies, making them a promising therapeutic target. RING ligases facilitate ubiquitin transfer from E2 enzymes to specific substrates. Thus, preventing RING-E3 recognition by E2 represents an attractive functional regulation strategy. However, addressing this with small molecules, including peptidomimetics, is challenging because of the particularly transient character of the interactions involved and the shallow, discontinuous, and mainly hydrophobic nature of the recognition site, which imposes a clear obstacle, risking poor and promiscuous ligand binding. Our rationale to overcome this issue has been primarily focused on comprehensively exploiting available structural information related to naturally occurring RING inhibitors and investigating the dynamics of the molecular mechanisms behind RING recognition by E2 enzymes.

The use of MD simulations has allowed the identification and selection of key recognition features ruling RING-E3/E2 recognition, and the obtained molecular models have been used to enable the inclusion of such features into suitable stable small nature-like molecular architectures able to mimic the tackled interactions. Through such a dynamic pharmacophore-based engineering strategy, integrating the identified common and specific RING recognition features and applying structure-based de novo design principles in an integrated computational and experimental approach, the initially designed scaffolds have been further rationally optimized for affinity and selectivity for RING E3 ligases, stability, and delivery.

We demonstrate the feasibility of such an approach by targeting protein recognition of APC/C, a large macromolecular complex containing a RING domain (APC11 subunit). Our strategy differs mechanistically from available APC/C inhibitors such as TAME and apcin, which target APC/C coactivators (i.e., CDC20 and Cdh1) and substrate recruitment, respectively. In this regard, our strategy is advantageous as APC/C coactivators use different mechanisms to recognize substrates.^10,11,52^

Through our interdisciplinary approach, dissecting the dynamic molecular mechanisms ruling the functional regulation of this challenging macro-complex, we have successfully *de novo* designed APC/C selective and noncompetitive inhibitors that prevent recognition of the E2 UBE2C and UBE2D. Notably, our designs demonstrated greater efficacy in inhibiting APC/C ubiquitination *in vitro* compared to TAME and apcin.

Taking advantage of their strong binding properties, the *de novo* designed molecules presented here are ideally suited to detect and/or purify RING-type E3 ligases (E2 mimetics), in particular, APC/C. Furthermore, these molecules could facilitate the identification of new RING-type E3 ligases and help to accelerate the development of future CRL inhibitors.

Our best APC/C inhibitor has successfully demonstrated the ability to prolong mitosis and induce cell death after a metaphase arrest in a subset of cancer cells. The observed heterogeneity in cell fate recapitulates profound intra- and interline variation in response to mitotic drugs in cancer cell lines^53^ and their differences in the expression of antiapoptotic proteins. For example, we found that HeLa cells with lower expression of Bcl-x­(L) are more prone to mitotic cell death than higher-expressing A549 cells (Figure 8B).^54^ Cell line-specific differences in the degree of endosomal escape could further contribute to heterogeneity in the treatment response.

Further optimization of its delivery into living cells (e.g., introducing specific cleavage site sequences of tumor-associated enzymes^55^ or combining with a mechanistically distinct APC/C inhibitor)^31^ holds great promise for effective therapeutic applications.

Overall, this work provides a strong foundation and rationale for selectively targeting ubiquitin E3 ligases and advancing the development of effective inhibitors.

## Experimental Section

### Peptide Synthesis

The designed E2 mimetics and *^i^* APC11 molecules were synthesized and purchased from Genscript Biotech (Leiden, The Netherlands) B.V. The designed BH, HT, HTH, and H compounds were dissolved according to the manufacturer’s instructions in water, while _EE1_H6 was dissolved in 10% DMSO. The concentration was measured by NanoDrop ND-1000 (Thermo Fisher Scientific) using the Protein A280 measurement setting. All compounds presented in this study are >95% pure by HPLC analysis, and the corresponding HPLC traces are included in the Supporting Information (Table S6).

### Computational Methods

#### Analysis of the RING Recognition Interfaces for E2 Enzymes and Natural Inhibitors

To target the APC/C subunit APC11, an atom-detailed comparative analysis of the following interfaces of RING proteins with E3 ligase activity in complex with E2 enzymes was carried out: APC11/UBE2C (PDB ID 5A31, 4.3 Å),^13^ RBX1/UBC12 (PDB ID 4P5O, 3.1 Å),^41^ c-CBL/UBE2L3 (PDB ID 1FBV, 2.9 Å),^56^ BIRC2/UBE2D2 (PDB ID 6HPR, 1.7 Å),^57^ and BMI1-RING1B/UBE2D3 (PDB ID 3RPG, 2.6 Å).^58^ The same analysis was also performed at the recognition interface of the known naturally occurring inhibitors EMI1 and Glomulin: APC11/EMI1 (PDB ID 4UI9, 3.6 Å)^13^ and RBX1/GLMN (PDB ID 4F52, 3.0 Å).^12^

#### Molecular Dynamics (MD) Simulations

MD simulations were used to identify recognition *hot spots* and select the most relevant recognition physicochemical features in the studied systems. MD simulations were also used to evaluate the binding of RING proteins to the *de novo* designed molecules (i.e., β-hairpin, α-helix-turn, α-helix-turn-α-helix, and α-helix (*i*, *i*+4 and *i*, *i*+7) molecules. For RBX1, coordinates were obtained from the most complete structure available (PDB ID 1LDJ, 3.0 Å)^42^ and used to investigate the interactions with UBEC12 and with the selected best *de novo* designed molecules.

MD simulations in AMBER15 ^59^ were used to refine the structures of the following complexes: APC11_L13‑E84_/UBE2C_G29‑Q173_, RBX1_L32‑Y106_/UBC12_A29‑H184_, c-CBL_C381–F434_/UBE2L3_S4–Y147_, BIRC2_L556‑S618_/UBE2D2_A2‑M147_, BMI1_R3‑H101_-RING1B_T16‑S116_/UBE2D3_L‑2‑M147_, RBX1_L32‑Q104_/GLMN_N279‑L432/K439‑L498/P550–K582_, APC11_L13‑E84_/Emi1_Y356–C419_, APC11_L13‑E84_/E2 mimetics, APC11_L13‑E84_/*^i^* APC11, RBX1_L32‑Y106_/E2 mimetics and RBX1_L32‑Y106_/*^i^* APC11. Charges and parameters were taken from the ff14SB^59^ and ZAFF^60^ force fields. RESP atomic charges^61,62^ were derived at the HF/6-31G­(d) calculation level for non-natural amino acid fragments, and at the B3LYP/6-311G++(d,p) level for RBX1 Zn^2+^-coordinated residues Cys75, His77, Cys94 and Asp97 using Gaussian09 ^63^ and Gaussian16.^64^ Missing parameters for non-natural amino acids were taken from the General Amber Force Field (GAFF),^65^ and those for Zn^2+^-coordinated residues were generated through MCPB.py.^66^ Each complex was solvated in a truncated octahedral box of TIP3P water molecules and neutralized with Na^+^ or Cl^–^ counterions. MD simulations were preceded by two energy-minimization steps, first with position restraints for the solute (500 kcal/mol Å^2^) using 1000 steps of steepest descendent and 500 steps of conjugate gradient minimization, second without restraints by applying 3000 cycles of steepest descendent and 3000 steps of conjugate gradient. Then, the system was heated up from 200 to 300K in 50 ps with weak position restraints (10 kcal/mol Å^2^). Langevin temperature coupling with a collision frequency of γ = 1 ps^–1^ was used at this step. The system was equilibrated under constant pressure of 1 atm using periodic boundary conditions (NPT conditions) at 300K for 500 ps. A total of 40 ns MD simulations were conducted at 300K NPT conditions for each complex. MD simulations of RING/E2 and selected APC11_L13‑E84_/E2 mimetics, APC11_L13‑E84_/*^i^* APC11, RBX1_L32‑Y106_/E2 mimetics, and RBX1_L32‑Y106_/*^i^* APC11 systems were conducted in triplicate for detailed evaluation (i.e., statistical analysis and reproducibility). The SHAKE algorithm with a time step of 2 fs was used to constrain all bonds involving hydrogen atoms. A cutoff of 8 Å was applied to treat the nonbonded interactions, and the Particle Mesh Ewald (PME) method was used to treat long-range electrostatic interactions. MD trajectories were recorded every 10 ps and visualized with VMD.^67^ The CPPTRAJ module implemented in AMBER was used to evaluate intermolecular contacts in terms of contact maps and intermolecular H-bonds. At least 10% of H-bond occupancy with a distance acceptor–donor cutoff of 3.5 Å and a 120° angle was taken as the criterion for dynamic H-bond formation in the last 20 ns of each MD simulation. Energy decomposition per residue, pairwise as well as binding free energy postprocessing analysis of 200 frames from the last 20 ns MD simulations were performed in implicit solvent using MM-GBSA^68,69^ as implemented in AMBER15. Data analysis was performed with Origin 2023.^70^ Figures were created with PyMOL.^71^

#### MD-Based Dynamic Pharmacophore Modeling

From each of the MD triplicates obtained for the systems APC11/UBE2C, APC11/Emi1, and RBX1/GLMN, ten structures were extracted from the last 20 ns. For comparison, the resulting 30 MD-refined structures were superimposed on the corresponding X-ray structure (*vide supra*). H-bond and per-residue MM-GBSA energetic analysis allowed the identification of key physicochemical features involved in protein recognition (i.e., aromatic, hydrophobic, H-bond acceptor, and cation/donor). The selection criteria for *hot spots* were based on the most favorable per-residue binding energy contribution and the detected interactions in the intermolecular contacts analysis. The static pharmacophore was constructed by identifying features defined by *hot spot* atoms disposed in close proximity in 3D space using the coordinates from an MD-average structure overlaid onto the corresponding crystallographic structure. The dynamic pharmacophore was built by using atomic coordinates extracted from the selected *hot spot* atoms within the superimposed MD-refined structures. For analysis, static and dynamic pharmacophores were represented by mesh spheres and dots, respectively.

#### Dynamic Pharmacophore-Based Rational Design and Rescaffolding Approach

The obtained dynamic pharmacophores were used to analyze geometric relationships and similarities among the studied systems (i.e., APC11/UBE2C, APC11/EMI1 and RBX1/GLMN) and set up the bases for our design rationale.

The spatial distributions of common RING recognition functionalities of UBE2C, EMI1, and GLMN were used for searching suitable molecular architectures at PDB,^34^ which were used as backbone templates for molecular modeling. Those residues already fitting the required pharmacophore features were kept, and the rest were customized as required. The 3D structures with PDB IDs 1KVF,^36^ 1IMW,^37^ 1DVA (3.0 Å),^38^ 4DJS (3.0 Å),^45^ and 4N5T (1.7 Å)^46^ were used as templates to build atomic models of the β-hairpin, α-helix-turn, helix-turn-helix, and (*i*, *i* + 4) and (*i*, *i* + 7) α-helix scaffolds, respectively.

Next, the scaffolds were manually docked to the APC11 structure (PDB ID 4UI9),^13^ using as reference the crystallographic coordinates of the complexes APC11/UBE2C, APC11/EMI1, and RBX1/GLMN, as well as the obtained MD-based pharmacophores. The scaffolds complexed to APC11 were modified in MOE^72^ to achieve binding and compel fold stability. The principle of maximum overlap in shape and physicochemical properties was used as a premise for the designs. The resulting molecules in complex with APC11 were refined by MD, and their recognition was analyzed in detail (see the MD section above).

#### RING Selectivity Design and Evaluation

The superimposed structures of APC11 (PDB ID 4UI9)^13^ and RBX1 (PDB IDs 4F52,^12^ 4P50,^41^ 1LDJ,^42^ and 2HYE (3.1 Å)^43^) were used to visualize their residue correspondences and spatial distribution of properties. The H α-helix scaffolds were manually docked to the E2 recognition site, and functionalities were rationally introduced to maximize structural complementarity toward APC11 while minimizing interactions toward RBX1_E55_, RBX1_R86_, RBX1_W87_, RBX1_R91_, and RBX1_N98_. The selected molecules with the best recognition properties toward APC11 were manually docked to the E2 recognition site of RBX1 (PDB ID 1LDJ)^42^ to obtain optimal shape complementarity. The resulting complexes were refined by MD and analyzed (see the MD section above).

### Cells and Proteins

Antibodies, cell lines, and methods for protein expression and purification are specified in the Supporting Information. No biological samples obtained from animals or human subjects beyond the use of well-established cell lines obtainable from ATCC were used in this study.

#### SDS-PAGE

Proteins were separated by SDS-PAGE electrophoresis using precast Bolt 4–12% Bis-Tris Plus protein gels (Thermo Fisher Scientific) and Criterion XT Bis-Tris Midi Protein Gels (Bio-Rad). In different experiments various conditions were used: *in vitro* APC/C ubiquitination assays–165 V, 40 min, SDS-MES running buffer (50 mM MES, 50 mM Tris base, 0.1% SDS, 1 mM EDTA, pH 7.3; Thermo Fisher Scientific); *in vitro* APC/C ubiquitination assays determining *K*_m_ – 165 V, 1 h, SDS-MOPS running buffer (50 mM MOPS, 50 mM Tris base, 0.1% SDS, 1 mM EDTA, pH 7.7; Thermo Fisher Scientific); pull-down binding assay −165 V, 35 min, SDS-MES running buffer.

#### Western Blot Analysis

For protein transfer, a 0.45 μm Immobilon-FL PVDF membrane (Merck Millipore) was used. The membrane was activated in ethanol and then transferred to Blotting buffer (50 mM SDS-MOPS, 50 mM Tris base, 0.1% SDS, 1 mM EDTA, and 20% EtOH). The proteins were transferred using a wet transfer for 1.5 h at 400 mA. Subsequently, the membrane was incubated with the blocking solution, 10% milk in PBS-T (0.02% Tween-20 in PBS), for 1 h at room temperature. To detect proteins of interest, the membrane was incubated with primary antibodies overnight at 4° C, followed by 3 washes for 10 min with PBS-T and incubation with IRDye-labeled secondary antibodies for 1 h. Prior to detection, the membrane was washed twice with PBS-T for 10 min and once with PBS for 10 min. The detection was done using the quantitative near-infrared scanning system Odyssey (LI-COR Biosciences).

#### Blue Silver Staining of SDS-PAGE Gels

The SDS-PAGE gels were washed with distilled water and incubated with Blue silver staining solution (10% phosphoric acid, 20% methanol, 10% ammonium sulfate, and 0.12% Coomassie Blue G-250, solution prepared in Milli-Q water) overnight. Afterward, the gel was destained with distilled water and imaged using the quantitative near-infrared scanning system Odyssey.

#### *In Vitro* APC/C Ubiquitination Assay

APC/C-dependent ubiquitination reactions were performed at 30 °C in 30 mM HEPES pH 7.4, 175 mM NaCl, 8 mM MgCl_2_, 0.05% Tween-20, 1 mM DTT and 5% glycerol and contained 20 nM recombinant APC/C, 340 nM CDC20, 46 nM GST-UBA1, 340 nM UBE2C (alternatively 400 nM UBE2D, 280 nM UBE2S), 21 μM His_6_-ubiquitin or if it is indicated 21 μM methylated-ubiquitin (BostonBiochem), 2.6 mM ATP, 10 mM phosphocreatine, and 11 μM creatine kinase. Note, in case of testing BH, HT, and HTH *^i^* APC11, APC/C^Cdc20^ immunoprecipitated from metaphase HeLa cells^73^ was used. As substrate, 50 nM fluorophore-labeled securin was standardly used (fluorophore IRDye 680RD and 800CW (LI-COR) was used for labeling). In the case of *K*_m_ and *k*_cat_ determination, 50, 100, 250, 500, 750,1000, 1250, 2500, and 5000 nM fluorophore-labeled securin was used. Note, to reduce autoubiquitination that can potentially inhibit UBE2C activity, the UBE2C^K119R^ mutant was used^73^ in all *in vitro* APC/C ubiquitination assays. The reaction was done in a volume of 15 μL, and it was quenched after the indicated time with LDS sample buffer (Thermo Fisher Scientific) supplemented with 100 mM DTT and subjected to SDS-PAGE. Detection of fluorescently labeled substrates was done by a quantitative near-infrared scanning system, Odyssey.

#### *In Vitro* E2 Activity Assay

UBE2C and UBE2S activity assays were performed at 30 °C in 50 mM HEPES pH 7.4, 100 mM KCl, 9 mM MgCl2, and contained 0.6 μM UBA1 (E1), 8 μM UBE2C or UBE2S, 60 μM His6-ubiquitin, and 5.6 mM ATP. It should be noted that wild-type UBE2C and UBE2S were used. The reaction was done in a volume of 15 μL, and it was quenched after 45 min with the LDS sample buffer supplemented with 100 mM DTT and subjected to SDS-PAGE followed by Blue Silver staining. Imaging of Blue Silver-stained gels was done by a quantitative near-infrared scanning system, Odyssey. It should be noted that reactions without ATP were first preincubated for 5 min at room temperature.

#### Pull-Down Binding Assay

HeLa cell pellet was resuspended in the extraction buffer (30 mM HEPES pH 7.5, 175 mM NaCl, 2.5 mM MgCl_2_, 0.25% NP40, 10% glycerol, 1 mM DTT supplied with complete protease inhibitor cocktail (Roche), PhosSTOP phosphatase inhibitors (Roche), 1 mM PMSF, 10 μM MG132) and incubated for 20 min on ice, followed by centrifugation to clear the lysate for 15 min at 4 °C 16,100 *g*. In the meantime, inhibitor molecules containing the propargyl-Gly-OH (Pra) chemical group were covalently attached to the azide agarose resin (Jena Bioscience) by Cu­(I)-catalyzed azide–alkyne cycloaddition reaction. Specifically, the inhibitor molecules were mixed with azide agarose resin in a ratio of 0.125 μmol inhibitor/20 μL agarose resin. The reaction was catalyzed by 0.1 mM CuSO_4_, 0.5 mM THPTA (Sigma-Aldrich), and 5 mM Sodium ascorbate and was performed on the rotating wheel for 30 min at room temperature in a volume of 1 mL. Subsequently, the resin was washed five times with extraction buffer. The inhibitor-agarose resin was added to 2 mg of HeLa cell extract and incubated at 4 °C for 2 h. Afterward, the resin was washed four times with extraction buffer. Pull-down proteins were eluted by incubation with prewarmed 1x LDS sample buffer for 10 min at room temperature, followed by taking supernatant supplemented with 100 mM DTT and boiling for 10 min. Afterward, SDS PAGE and Western blot analyses were performed to detect the indicated proteins.

#### Click-iT Detection of *^i^* APC11

Cells grown in a 96-well plate were incubated for 24 or 48 h with APC11 inhibitors containing propargyl-Gly-OH (Pra) employed for click chemistry reactions. Cells were fixed with 4% paraformaldehyde (VWR) and permeabilized with the buffer composed of 0.1% Triton (Sigma-Aldrich) and 0.02% SDS (VWR) in two consecutive steps, each for 15 min. Afterward, the Click-iT reactions were performed using Click-iT Plus Alexa Fluor 647 Picolyl Azide Toolkit (Thermo Fisher Scientific) according to the manufacturer′s instructions. The Click-iT Plus reaction cocktail per a single reaction (a single well) was 30 μL, keeping the ratio of all components as it was described in the manufacturer′s instructions. The CuSO_4_-copper protectant premix was prepared in the ratio 1:1. The Click-iT Plus reaction cocktail was incubated with cells for 30 min and then washed three times for 10 min with PBS. Finally, DNA was counterstained with 4′,6-diamidino-2-phenylindole dihydrochloride (DAPI; Sigma-Aldrich) for 10 min. All of the steps were performed at room temperature. Fluorescent images were acquired using an ImageXpress Micro XLS wide-field screening microscope. For the localization experiments, HeLa cells were seeded in 96-well plates and treated on the following day with vehicle or 50 μM of Pra-H1, Pra-H6, and Pra-_EE1_H6 for 4 or 10 h. After incubation, the cells were fixed and permeabilized before blocking with 3% BSA, 0.02% Tween-20 in PBS. Click chemistry was performed in 1 mM sodium ascorbate, 0.1 mM TBTA, 1 mM CuSO4, and 2 μM AZDye488-picolyl-azide (Jena Bioscience) for 1 h, and the reaction was quenched by washing with 20 mM EDTA, 0.01% Triton X-100 in PBS for 10 min. Immunofluorescent staining was performed with an anti-Rab5 primary antibody (C8B1, Cell Signaling) diluted in 2% BSA, 0.02% Tween-20 in PBS, Alexa Fluor 568 secondary antibody (Invitrogen), and counterstaining with DAPI. Images were acquired using ImageXpress at 20× magnification, and the line scan was generated by Fiji using the RGB Profiler. Nuclear-localized inhibitors were quantified by segmenting nuclei based on DAPI and quantifying 488 intensities within the nuclear mask.

### Statistical Analysis

Prism 10 (GraphPad) was used for statistical analysis. All applied statistical analyses and respective statistical information are indicated in the respective figure legends. The inhibition constant (*K*_i_) was calculated using a model for noncompetitive inhibition in Prism 6.0:

$$V_{\max\;\mathrm{inh}}=\frac{V_{\max}}{\left(1+\frac{I}{K_{i}}\right)}\;v=\frac{V_{\max\;\mathrm{inh}}\left[S\right]}{\left(K_{m}+\left[S\right]\right)}$$

*V*_max inh_ is a maximum rate of reaction (also termed maximum enzyme velocity) in the presence of an inhibitor, *V*_max_ is a maximum rate of reaction without an inhibitor, *I* is the concentration of an inhibitor, *K*_i_ is the inhibition constant, *v* is a rate of reaction, *S* is the substrate concentration, and *K*_m_ is the Michaelis–Menten constant.

## Supplementary Material



## Notes

Details of antibodies and cell lines; methods for live cell imaging of cells treated with *^i^* APC11; methods for protein expression and purification; additional information related to RING proteins recognition by E2 enzymes and natural inhibitors; additional information of interaction analysis for *^i^* APC11 molecules in complex with APC11 and RBX1 proteins; additional ubiquitination assays using Cyclin B1 as the substrate and different E2 enzymes; experimental details of IC_50_ and kinetics evaluation for compound H6; experimental determination of length of mitosis and mitotic arrest in metaphase for selected *^i^* APC11; and HPLC chromatograms of *^i^* APC11 (PDF)

## ∥

DIANA Biotechnologies a.s., 25250 Vestec, Prague, Czechia

## Abbreviations

APC/C: anaphase promoting complex/cyclosome
APC11: anaphase promoting complex subunit 11
CRLs: cullin RING ligases
EMI1: early mitotic inhibitor 1
GLMN: glomuline
MD simulations: molecular dynamics simulations
Pra: l-propargylglycine
RING: really interesting new gene
TAME: tosyl-l-arginine methyl ester
3D: three-dimensional
vdW: van der Waals

## References

[1] Aebersold R., Agar J. N., Amster I. J., Baker M. S., Bertozzi C. R., Boja E. S., Costello C. E., Cravatt B. F., Fenselau C., Garcia B. A., Ge Y., Gunawardena J., Hendrickson R. C., Hergenrother P. J., Huber C. G., Ivanov A. R., Jensen O. N., Jewett M. C., Kelleher N. L., Kiessling L. L., Krogan N. J., Larsen M. R., Loo J. A., Ogorzalek
Loo R. R., Lundberg E., MacCoss M. J., Mallick P., Mootha V. K., Mrksich M., Muir T. W., Patrie S. M., Pesavento J. J., Pitteri S. J., Rodriguez H., Saghatelian A., Sandoval W., Schluter H., Sechi S., Slavoff S. A., Smith L. M., Snyder M. P., Thomas P. M., Uhlen M., Van Eyk J. E., Vidal M., Walt D. R., White F. M., Williams E. R., Wohlschlager T., Wysocki V. H., Yates N. A., Young N. L., Zhang B. (2018). How many human
proteoforms are there?. Nat. Chem. Biol..

[2] Li Z., Li S., Luo M., Jhong J. H., Li W., Yao L., Pang Y., Wang Z., Wang R., Ma R., Yu J., Huang Y., Zhu X., Cheng Q., Feng H., Zhang J., Wang C., Hsu J. B., Chang W. C., Wei F. X., Huang H. D., Lee T. Y. (2022). dbPTM in 2022: an
updated database for exploring regulatory networks and functional
associations of protein post-translational modifications. Nucleic Acids Res..

[3] Deshaies R. J., Joazeiro C. A. (2009). RING domain E3 ubiquitin
ligases. Annu. Rev. Biochem..

[4] Liu L., Damerell D. R., Koukouflis L., Tong Y., Marsden B. D., Schapira M. (2019). UbiHub: a data hub
for the explorers of ubiquitination
pathways. Bioinformatics.

[5] Plechanovova A., Jaffray E. G., Tatham M. H., Naismith J. H., Hay R. T. (2012). Structure
of a RING E3 ligase and ubiquitin-loaded E2 primed for catalysis. Nature.

[6] Jin J., Cardozo T., Lovering R. C., Elledge S. J., Pagano M., Harper J. W. (2004). Systematic analysis
and nomenclature of mammalian F-box
proteins. Genes Dev..

[7] Pines J. (2011). Cubism and
the cell cycle: the many faces of the APC/C. Nat. Rev. Mol. Cell Biol..

[8] Zhang S., Chang L., Alfieri C., Zhang Z., Yang J., Maslen S., Skehel M., Barford D. (2016). Molecular mechanism
of APC/C activation by mitotic phosphorylation. Nature.

[9] Spratt D. E., Wu K., Kovacev J., Pan Z. Q., Shaw G. S. (2012). Selective recruitment
of an E2 ∼ ubiquitin complex by an E3 ubiquitin ligase. J. Biol. Chem..

[10] Alfieri C., Zhang S., Barford D. (2017). Visualizing
the complex functions
and mechanisms of the anaphase promoting complex/cyclosome (APC/C). Open Biol..

[11] Barford D. (2020). Structural
interconversions of the anaphase-promoting complex/cyclosome (APC/C)
regulate cell cycle transitions. Curr. Opin.
Struct. Biol..

[12] Duda D. M., Olszewski J. L., Tron A. E., Hammel M., Lambert L. J., Waddell M. B., Mittag T., DeCaprio J. A., Schulman B. A. (2012). Structure
of a glomulin-RBX1-CUL1 complex: inhibition of a RING E3 ligase through
masking of its E2-binding surface. Mol. Cell.

[13] Chang L., Zhang Z., Yang J., McLaughlin S. H., Barford D. (2015). Atomic structure of the APC/C and its mechanism of
protein ubiquitination. Nature.

[14] McIntyre B. A., Brouillard P., Aerts V., Gutierrez-Roelens I., Vikkula M. (2004). Glomulin is predominantly expressed in vascular smooth
muscle cells in the embryonic and adult mouse. Gene Expr. Patterns.

[15] Frye J. J., Brown N. G., Petzold G., Watson E. R., Grace C. R., Nourse A., Jarvis M. A., Kriwacki R. W., Peters J. M., Stark H., Schulman B. A. (2013). Electron microscopy
structure of
human APC/C­(CDH1)-EMI1 reveals multimodal mechanism of E3 ligase shutdown. Nat. Struct. Mol. Biol..

[16] Qiu L., Wu J., Pan C., Tan X., Lin J., Liu R., Chen S., Geng R., Huang W. (2016). Downregulation of CDC27
inhibits the proliferation of colorectal cancer cells via the accumulation
of p21Cip1/Waf1. Cell Death Dis..

[17] Zhou J., Zhang S., Fu G., He Z., Xu Y., Ye W., Chen Z. (2018). Overexpression of APC11
predicts worse survival in
lung adenocarcinoma. Onco Targets Ther..

[18] Maes A., Maes K., De Raeve H., De Smedt E., Vlummens P., Szablewski V., Devin J., Faict S., De Veirman K., Menu E., Offner F., Spaargaren M., Moreaux J., Vanderkerken K., Van Valckenborgh E., De Bruyne E. (2019). The anaphase-promoting complex/cyclosome: a new promising
target in diffuse large B-cell lymphoma and mantle cell lymphoma. Br. J. Cancer.

[19] Kidokoro T., Tanikawa C., Furukawa Y., Katagiri T., Nakamura Y., Matsuda K. (2008). CDC20, a potential
cancer therapeutic target, is negatively
regulated by p53. Oncogene.

[20] Wu W. J., Hu K. S., Wang D. S., Zeng Z. L., Zhang D. S., Chen D. L., Bai L., Xu R. H. (2013). CDC20 overexpression
predicts a poor prognosis for patients with colorectal cancer. J. Transl. Med..

[21] Marucci G., Morandi L., Magrini E., Farnedi A., Franceschi E., Miglio R., Calo D., Pession A., Foschini M. P., Eusebi V. (2008). Gene expression profiling
in glioblastoma and immunohistochemical
evaluation of IGFBP-2 and CDC20. Virchows Arch..

[22] Kato T., Daigo Y., Aragaki M., Ishikawa K., Sato M., Kaji M. (2012). Overexpression of CDC20 predicts poor prognosis in primary non-small
cell lung cancer patients. J. Surg. Oncol..

[23] Chang D. Z., Ma Y., Ji B., Liu Y., Hwu P., Abbruzzese J. L., Logsdon C., Wang H. (2012). Increased
CDC20 expression is associated
with pancreatic ductal adenocarcinoma differentiation and progression. J. Hematol. Oncol..

[24] Luo J., Emanuele M. J., Li D., Creighton C. J., Schlabach M. R., Westbrook T. F., Wong K. K., Elledge S. J. (2009). A genome-wide
RNAi screen identifies multiple synthetic lethal interactions with
the Ras oncogene. Cell.

[25] Ayesha A. K., Hyodo T., Asano E., Sato N., Mansour M. A., Ito S., Hamaguchi M., Senga T. (2016). UBE2S is associated with malignant
characteristics of breast cancer cells. Tumour
Biol..

[26] Liang J., Nishi H., Bian M. L., Higuma C., Sasaki T., Ito H., Isaka K. (2012). The ubiquitin-conjugating
enzyme E2-EPF is overexpressed
in cervical cancer and associates with tumor growth. Oncol. Rep..

[27] Liu G., Zhao J., Pan B., Ma G., Liu L. (2019). UBE2C overexpression
in melanoma and its essential role in G2/M transition. J. Cancer.

[28] van
Ree J. H., Jeganathan K. B., Malureanu L., van Deursen J. M. (2010). Overexpression of the E2 ubiquitin-conjugating enzyme
UbcH10 causes chromosome missegregation and tumor formation. J. Cell Biol..

[29] Wang L., Liang Y., Li P., Liang Q., Sun H., Xu D., Hu W. (2019). Oncogenic
Activities Of UBE2S Mediated By VHL/HIF-1alpha/STAT3
Signal Via The Ubiquitin-Proteasome System In PDAC. Onco Targets Ther..

[30] Schrock M. S., Stromberg B. R., Scarberry L., Summers M. K. (2020). APC/C ubiquitin
ligase: Functions and mechanisms in tumorigenesis. Semin. Cancer Biol..

[31] Sackton K. L., Dimova N., Zeng X., Tian W., Zhang M., Sackton T. B., Meaders J., Pfaff K. L., Sigoillot F., Yu H., Luo X., King R. W. (2014). Synergistic blockade of mitotic exit
by two chemical inhibitors of the APC/C. Nature.

[32] Zeng X., Sigoillot F., Gaur S., Choi S., Pfaff K. L., Oh D. C., Hathaway N., Dimova N., Cuny G. D., King R. W. (2010). Pharmacologic inhibition of the anaphase-promoting
complex induces a spindle checkpoint-dependent mitotic arrest in the
absence of spindle damage. Cancer Cell.

[33] Lub S., Maes A., Maes K., De Veirman K., De Bruyne E., Menu E., Fostier K., Kassambara A., Moreaux J., Hose D., Leleu X., King R. W., Vanderkerken K., Van Valckenborgh E. (2016). Inhibiting the anaphase promoting
complex/cyclosome induces a metaphase arrest and cell death in multiple
myeloma cells. Oncotarget.

[34] Ruiz-Gómez G., Hawkins J. C., Philipp J., Künze G., Wodtke R., Löser R., Fahmy K., Pisabarro M. T. (2016). Rational
Structure-Based Rescaffolding Approach to De Novo Design of Interleukin
10 (IL-10) Receptor-1 Mimetics. PLoS One.

[35] Fasan R., Dias R. L., Moehle K., Zerbe O., Vrijbloed J. W., Obrecht D., Robinson J. A. (2004). Using a beta-hairpin
to mimic an
alpha-helix: cyclic peptidomimetic inhibitors of the p53-HDM2 protein-protein
interaction. Angew. Chem., Int. Ed. Engl..

[36] Skelton N. J., Russell S., de Sauvage F., Cochran A. G. (2002). Amino acid determinants
of beta-hairpin conformation in erythropoeitin receptor agonist peptides
derived from a phage display library. J. Mol.
Biol..

[37] Skelton N. J., Chen Y. M., Dubree N., Quan C., Jackson D. Y., Cochran A., Zobel K., Deshayes K., Baca M., Pisabarro M. T., Lowman H. B. (2001). Structure-function analysis of a
phage display-derived peptide that binds to insulin-like growth factor
binding protein 1. Biochemistry.

[38] Dennis M.
S., Eigenbrot C., Skelton N. J., Ultsch M. H., Santell L., Dwyer M. A., O’Connell M. P., Lazarus R. A. (2000). Peptide exosite
inhibitors of factor VIIa as anticoagulants. Nature.

[39] Russell S. J., Blandl T., Skelton N. J., Cochran A. G. (2003). Stability of cyclic
beta-hairpins: asymmetric contributions from side chains of a hydrogen-bonded
cross-strand residue pair. J. Am. Chem. Soc..

[40] Vodermaier H. C., Gieffers C., Maurer-Stroh S., Eisenhaber F., Peters J. M. (2003). TPR subunits of
the anaphase-promoting complex mediate
binding to the activator protein CDH1. Curr.
Biol..

[41] Scott D. C., Sviderskiy V. O., Monda J. K., Lydeard J. R., Cho S. E., Harper J. W., Schulman B. A. (2014). Structure of a RING
E3 trapped in
action reveals ligation mechanism for the ubiquitin-like protein NEDD8. Cell.

[42] Zheng N., Schulman B. A., Song L., Miller J. J., Jeffrey P. D., Wang P., Chu C., Koepp D. M., Elledge S. J., Pagano M., Conaway R. C., Conaway J. W., Harper J. W., Pavletich N. P. (2002). Structure of the Cul1-Rbx1-Skp1-F
boxSkp2 SCF ubiquitin
ligase complex. Nature.

[43] Angers S., Li T., Yi X., MacCoss M. J., Moon R. T., Zheng N. (2006). Molecular
architecture and assembly of the DDB1-CUL4A ubiquitin ligase machinery. Nature.

[44] Walensky L. D., Bird G. H. (2014). Hydrocarbon-stapled
peptides: principles, practice,
and progress. J. Med. Chem..

[45] Grossmann T. N., Yeh J. T., Bowman B. R., Chu Q., Moellering R. E., Verdine G. L. (2012). Inhibition of oncogenic
Wnt signaling through direct
targeting of beta-catenin. Proc. Natl. Acad.
Sci. U. S. A..

[46] Chang Y. S., Graves B., Guerlavais V., Tovar C., Packman K., To K. H., Olson K. A., Kesavan K., Gangurde P., Mukherjee A., Baker T., Darlak K., Elkin C., Filipovic Z., Qureshi F. Z., Cai H., Berry P., Feyfant E., Shi X. E., Horstick J., Annis D. A., Manning A. M., Fotouhi N., Nash H., Vassilev L. T., Sawyer T. K. (2013). Stapled
alpha-helical peptide drug development: a potent
dual inhibitor of MDM2 and MDMX for p53-dependent cancer therapy. Proc. Natl. Acad. Sci. U. S. A..

[47] Bodrug T., Welsh K. A., Bolhuis D. L., Paulsmall C. E., Martinez-Chacin R. C., Liu B., Pinkin N., Bonacci T., Cui L., Xu P., Roscow O., Amann S. J., Grishkovskaya I., Emanuele M. J., Harrison J. S., Steimel J. P., Hahn K. M., Zhang W., Zhong E. D., Haselbach D., Brown N. G. (2023). Time-resolved cryo-EM (TR-EM) analysis of substrate
polyubiquitination by the RING E3 anaphase-promoting complex/cyclosome
(APC/C). Nat. Struct. Mol. Biol..

[48] Brown N. G., VanderLinden R., Watson E. R., Weissmann F., Ordureau A., Wu K. P., Zhang W., Yu S., Mercredi P. Y., Harrison J. S., Davidson I. F., Qiao R., Lu Y., Dube P., Brunner M. R., Grace C. R. R., Miller D. J., Haselbach D., Jarvis M. A., Yamaguchi M., Yanishevski D., Petzold G., Sidhu S. S., Kuhlman B., Kirschner M. W., Harper J. W., Peters J. M., Stark H., Schulman B. A. (2016). Dual RING
E3 Architectures Regulate Multiubiquitination
and Ubiquitin Chain Elongation by APC/C. Cell.

[49] Brooks, H. B. ; Geeganage, S. ; Kahl, S. D. ; Montrose, C. ; Sittampalam, S. ; Smith, M. C. ; Weidner, J. R. Basics of Enzymatic Assays for HTS. In Assay Guidance Manual; Markossian, S. ; Grossman, A. ; Arkin, M. ; Auld, D. ; Austin, C. ; Baell, J. ; Brimacombe, K. ; Chung, T. D. Y. ; Coussens, N. P. ; Dahlin, J. L. ; Devanarayan, V. ; Foley, T. L. ; Glicksman, M. ; Gorshkov, K. ; Haas, J. V. ; Hall, M. D. ; Hoare, S. ; Inglese, J. ; Iversen, P. W. ; Lal-Nag, M. ; Li, Z. ; Manro, J. R. ; McGee, J. ; McManus, O. ; Pearson, M. ; Riss, T. ; Saradjian, P. ; Sittampalam, G. S. ; Tarselli, M. ; Trask, O. J., Jr. ; Weidner, J. R. ; Wildey, M. J. ; Wilson, K. ; Xia, M. ; Xu, X. , Eds.; Eli Lilly & Company and the National Center for Advancing Translational Sciences: Bethesda, MD, 2004. 22553875

[50] Daniel K., Icha J., Horenburg C., Muller D., Norden C., Mansfeld J. (2018). Conditional control
of fluorescent protein degradation
by an auxin-dependent nanobody. Nat. Commun..

[51] Lonn P., Kacsinta A. D., Cui X. S., Hamil A. S., Kaulich M., Gogoi K., Dowdy S. F. (2016). Enhancing
Endosomal Escape for Intracellular
Delivery of Macromolecular Biologic Therapeutics. Sci. Rep..

[52] Di
Fiore B., Davey N. E., Hagting A., Izawa D., Mansfeld J., Gibson T. J., Pines J. (2015). The ABBA motif binds
APC/C activators and is shared by APC/C substrates and regulators. Dev. Cell.

[53] Gascoigne K. E., Taylor S. S. (2008). Cancer cells display profound intra-
and interline
variation following prolonged exposure to antimitotic drugs. Cancer Cell.

[54] Galan-Malo P., Vela L., Gonzalo O., Calvo-Sanjuan R., Gracia-Fleta L., Naval J., Marzo I. (2012). Cell fate after mitotic
arrest in different tumor cells is determined by the balance between
slippage and apoptotic threshold. Toxicol. Appl.
Pharmacol..

[55] Singh Y., Palombo M., Sinko P. J. (2008). Recent trends in
targeted anticancer
prodrug and conjugate design. Curr. Med. Chem..

[56] Zheng N., Wang P., Jeffrey P. D., Pavletich N. P. (2000). Structure
of a c-Cbl-UbcH7 complex: RING domain function in ubiquitin-protein
ligases. Cell.

[57] Patel A., Sibbet G. J., Huang D. T. (2019). Structural
insights into non-covalent
ubiquitin activation of the cIAP1-UbcH5B approximately ubiquitin complex. J. Biol. Chem..

[58] Bentley M. L., Corn J. E., Dong K. C., Phung Q., Cheung T. K., Cochran A. G. (2011). Recognition of UbcH5c
and the nucleosome by the Bmi1/Ring1b
ubiquitin ligase complex. EMBO J..

[59] Case, D. A. ; Berryman, J. T. ; Betz, R. M. ; Cerutti, D. S. ; Cheatham, T. E., III ; Darden, T. A. ; Duke, R. E. ; Giese, T. J. ; Gohlke, H. ; Goetz, A. W. ; Homeyer, N. ; Izadi, S. ; Janowski, P. ; Kaus, J. ; Kovalenko, A. ; Lee, T. S. ; LeGrand, S. ; Li, P. ; Luchko, T. ; Luo, R. ; Madej, B. ; Merz, K. M. ; Monard, G. ; Needham, P. ; Nguyen, H. ; Nguyen, H. T. ; Omelyan, I. ; Onufriev, A. ; Roe, D. R. ; Roitberg, A. ; Salomon-Ferrer, R. ; Simmerling, C. L. ; Smith, W. ; Swails, J. ; Walker, R. C. ; Wang, J. ; Wolf, R. M. ; Wu, X. ; York, D. M. ; Kollman, P. A. AMBER15; University of California: San Francisco, 2015.

[60] Peters M.
B., Yang Y., Wang B., Fusti-Molnar L., Weaver M. N., Merz K. M. (2010). Structural Survey
of Zinc Containing Proteins and the Development of the Zinc AMBER
Force Field (ZAFF). J. Chem. Theory Comput..

[61] Bayly C. I., Cieplak P., Cornell W. D., Kollman P. A. (1993). A Well-Behaved Electrostatic
Potential Based Method Using Charge Restraints for Deriving Atomic
Charges - the Resp Model. J. Phys. Chem..

[62] Dupradeau F.-Y., Pigache A., Zaffran T., Savineau C., Lelong R., Grivel N., Lelong D., Rosanski W., Cieplak P. (2010). The R.E.D.
tools: advances in RESP and ESP charge derivation and force field
library building. Phys. Chem. Chem. Phys..

[63] Frisch, M. J. ; Trucks, G. W. ; Schlegel, H. B. ; Scuseria, G. E. ; Robb, M. A. ; Cheeseman, J. R. ; Scalmani, G. ; Barone, V. ; Mennucci, B. ; Petersson, G. A. ; Nakatsuji, H. ; Caricato, M. ; Li, X. ; Hratchian, H. P. ; Izmaylov, A. F. ; Bloino, J. ; Zheng, G. ; Sonnenberg, J. L. ; Hada, M. ; Ehara, M. ; Toyota, K. ; Fukuda, R. ; Hasegawa, J. ; Ishida, M. ; Nakajima, T. ; Honda, Y. ; Kitao, O. ; Nakai, H. ; Vreven, T. ; Montgomery, J. A., Jr. ; Peralta, J. E. ; Ogliaro, F. ; Bearpark, M. J. ; Heyd, J. ; Brothers, E. N. ; Kudin, K. N. ; Staroverov, V. N. ; Kobayashi, R. ; Normand, J. ; Raghavachari, K. ; Rendell, A. P. ; Burant, J. C. ; Iyengar, S. S. ; Tomasi, J. ; Cossi, M. ; Rega, N. ; Millam, N. J. ; Klene, M. ; Knox, J. E. ; Cross, J. B. ; Bakken, V. ; Adamo, C. ; Jaramillo, J. ; Gomperts, R. ; Stratmann, R. E. ; Yazyev, O. ; Austin, A. J. ; Cammi, R. ; Pomelli, C. ; Ochterski, J. W. ; Martin, R. L. ; Morokuma, K. ; Zakrzewski, V. G. ; Voth, G. A. ; Salvador, P. ; Dannenberg, J. J. ; Dapprich, S. ; Daniels, A. D. ; Farkas, Ö. ; Foresman, J. B. ; Ortiz, J. V. ; Cioslowski, J. ; Fox, D. J. Gaussian 09, Revision C.01; Gaussian, Inc.: Wallingford, CT, USA, 2009.

[64] Frisch, M. J. ; Trucks, G. W. ; Schlegel, H. B. ; Scuseria, G. E. ; Robb, M. A. ; Cheeseman, J. R. ; Scalmani, G. ; Barone, V. ; Petersson, G. A. ; Nakatsuji, H. ; Li, X. ; Caricato, M. ; Marenich, A. V. ; Bloino, J. ; Janesko, B. G. ; Gomperts, R. ; Mennucci, B. ; Hratchian, H. P. ; Ortiz, J. V. ; Izmaylov, A. F. ; Sonnenberg, J. L. ; Williams; Ding, F. ; Lipparini, F. ; Egidi, F. ; Goings, J. ; Peng, B. ; Petrone, A. ; Henderson, T. ; Ranasinghe, D. ; Zakrzewski, V. G. ; Gao, J. ; Rega, N. ; Zheng, G. ; Liang, W. ; Hada, M. ; Ehara, M. ; Toyota, K. ; Fukuda, R. ; Hasegawa, J. ; Ishida, M. ; Nakajima, T. ; Honda, Y. ; Kitao, O. ; Nakai, H. ; Vreven, T. ; Throssell, K. ; Montgomery, J. A., Jr ; Peralta, J. E. ; Ogliaro, F. ; Bearpark, M. J. ; Heyd, J. J. ; Brothers, E. N. ; Kudin, K. N. ; Staroverov, V. N. ; Keith, T. A. ; Kobayashi, R. ; Normand, J. ; Raghavachari, K. ; Rendell, A. P. ; Burant, J. C. ; Iyengar, S. S. ; Tomasi, J. ; Cossi, M. ; Millam, J. M. ; Klene, M. ; Adamo, C. ; Cammi, R. ; Ochterski, J. W. ; Martin, R. L. ; Morokuma, K. ; Farkas, O. ; Foresman, J. B. ; Fox, D. J. Gaussian 16 Rev. C.01; Gaussian, Inc.: Wallingford, CT, USA, 2016.

[65] Wang J., Wolf R. M., Caldwell J. W., Kollman P. A., Case D. A. (2004). Development
and testing of a general amber force field. J. Comput. Chem..

[66] Li P., Merz K. M. (2016). MCPB.py: A Python Based Metal Center
Parameter Builder. J. Chem. Inf. Model..

[67] Humphrey W., Dalke A., Schulten K. (1996). VMD: Visual molecular dynamics. J. Mol. Graph. Model..

[68] Miller B. R., McGee T. D., Swails J. M., Homeyer N., Gohlke H., Roitberg A. E. (2012). MMPBSA.py: an efficient
program for end-state free energy calculations. J. Chem. Theory Comput..

[69] Wang J. M., Morin P., Wang W., Kollman P. A. (2001). Use of MM-PBSA in
reproducing the binding free energies to HIV-1 RT of TIBO derivatives
and predicting the binding mode to HIV-1 RT of efavirenz by docking
and MM-PBSA. J. Am. Chem. Soc..

[70] OriginPro, Version 2023; OriginLab Corporation: Northampton, MA, USA, 2023.

[71] Schrödinger L.L.C . The PyMOL Molecular Graphics System, Version 2.4.1; Schrödinger L.L.C: 2020.

[72] Chemical Computing Group Inc . Molecular Operating Environment (MOE), version 2019; Chemical Computing Group Inc.: Montreal, QC, Canada, 2019.

[73] Bakos G., Yu L., Gak I. A., Roumeliotis T. I., Liakopoulos D., Choudhary J. S., Mansfeld J. (2018). An E2-ubiquitin thioester-driven
approach to identify substrates modified with ubiquitin and ubiquitin-like
molecules. Nat. Commun..

