# Supplementary material for: De Novo-Designed APC/C Inhibitors Provide a Rationale for Targeting RING-Type E3 Ubiquitin Ligases
Source: J Med Chem. 2025 May 21;68(11):11468–83. doi: 10.1021/acs.jmedchem.5c00416 (PMC12169662; doi:10.1021/acs.jmedchem.5c00416)
Supplement: Supplementary file 1 [file jm5c00416_si_001.pdf]

Supporting Information

De Novo-Designed APC/C Inhibitors Provide a Rationale for Targeting RING-Type E3 Ubiquitin Ligases

Gloria Ruiz-Gómez[a‡], Alena Uvizl[b‡†], Gabor Bakos[b], Jacky K. Leung[c], M. Teresa Pisabarro[a]*, and Jörg Mansfeld[bc]*

[a]Structural Bioinformatics, Biotechnology Center (BIOTEC), TU Dresden, 01307 Dresden, Germany. [b]Cell Cycle, Biotechnology Center (BIOTEC), TU Dresden, 01307 Dresden, Germany. [c]Division of Cell and Molecular Biololgy, Chester Beatty Laboratories, The Institute of Cancer Research, London, UK. [†] *Present address*: DIANA Biotechnologies a.s., 25250 Vestec, Prague, Czechia.

[‡]Equal contributing authors

E-mail: maria_teresa.pisabarro@tu-dresden.de and jorg.mansfeld@icr.ac.uk

Table of contents

Materials and Methods S4

Figure S1........S7

Figure S2........S8

Figure S3........S9

Figure S4........S10

Figure S5........S11

Figure S6........S11

Figure S7........S12

Figure S8........S13

Figure S9........S14

Figure S10........S15

Figure S11........S16

Figure S12........S17

Figure S13........S18

Figure S14........S19

Figure S15........S20

Figure S16........S21

Figure S17........S22

Figure S18........S24

Figure S19........S25

Table S1........S26

Table S2........S27

Table S3........S27

Table S4........S28

Table S5........S28

Table S6........S29

Purities and chromatograms of ^{i}APC11 compounds..S31

References for Supporting Information...S52

Materials and Methods

Antibodies

Primary antibodies

Antibody target	Product no.	Company	Species	Dilution
APC2	12301S	Cell Signalling	Rabbit monoclonal	WB 1:1000
APC11	14090	Cell Signalling	Rabbit monoclonal	WB 1:1000
BMI1	6964	Cell Signalling	Rabbit monoclonal	WB 1:1000
BRCA1	9010	Cell Signalling	Rabbit polyclonal	WB 1:1000
c-CBL	2747	Cell Signalling	Rabbit polyclonal	WB 1:1000
c-IAP1	7065	Cell Signalling	Rabbit monoclonal	WB 1:1000
GAPDH	2118	Cell Signalling	Rabbit polyclonal	WB 1:5000
MDM2	86934	Cell Signalling	Rabbit monoclonal	WB 1:1000
RAB5	3547	Cell Signalling	Rabbit monoclonal	IF: 1:200
RBX1	11922	Cell Signalling	Rabbit monoclonal	WB 1:1000

Secondary antibodies

Antibody target	Product no.	Company	Species	Dilution
anti-rabbit IgG, IRDye 800CW conjugated antibody	926-32213	LI-COR Biosciences	donkey	1:20000
anti-mouse IgG, IRDye 800CW conjugated antibody	926-32212	LI-COR Biosciences	donkey	1:20000
anti-rabbit IgG, IRDye 680RD conjugated antibody	926-68073	LI-COR Biosciences	donkey	1:20000
anti-mouse IgG, IRDye 680RD conjugated antibody	926-68072	LI-COR Biosciences	donkey	1:20000
anti-rabbit IgG, Alexa Fluor 568 conjugated antibody	A10037	Invitrogen	donkey	1:5000

Cell lines

Cells were cultured according to the standard mammalian tissue culture protocol and sterile technique at 37 °C in 5% CO_2 and tested in regular intervals for mycoplasma.

A549, HeLa cells (strain Kyoto) and SW480 cells were maintained in DMEM (Thermo Fisher Scientific) supplemented with 10% (v/v) fetal bovine serum (FBS) (Thermo Fisher Scientific), 1% (v/v) penicillin-streptomycin (Sigma-Aldrich), and 1% (v/v) Glutamax (Thermo Fisher Scientific).

HT-1080, MDA-MB-231 and RKO cells were maintained in RPMI 1640 (Thermo Fisher Scientific) supplemented with 10% (v/v) FBS and 1% (v/v) penicillin-streptomycin.

hTERT RPE-1 cells were maintained in DMEM/F12 (Sigma-Aldrich), supplemented with 10% (v/v) normal or tetracycline-free FBS (Gibco), 1% (v/v) penicillin-streptomycin, 1% (v/v) Glutamax, 0.26% (v/v) sodium bicarbonate (Gibco) and 0.5 µg/mL amphotericin B.

Live cell imaging

Automated time-lapse microscopy was performed using ImageXpress Micro XLS wide-field screening microscope (Molecular Devices) equipped with a 10x, 0.5 NA., 20x, 0.7 NA, and 40x, 0.95 NA Plan Apo air objectives (Nikon), a laser-based autofocus and a full environmental control (5% CO_2, 37 °C). Cells were grown in 96-well plastic bottom plates (µclear, Greiner Bio-One) and for live cell imaging media was changed to imaging media. Imaging media for A549, HeLa, hTERT RPE-1 and SW480 was DMEM without phenol red and riboflavin (Thermo Fisher Scientific), supplemented with 10% (v/v) FBS, 1% (v/v) Glutamax, and 1% (v/v) penicillin-streptomycin; imaging media for HT-1080, MDA-MB-231 and RKO was RPMI 1640 without phenol red (Thermo Fisher Scientific), supplemented with 10% (v/v) FBS, and 1% (v/v) penicillin-streptomycin.

Live cell imaging of cells treated with ^{i}APC11

Cells were seeded into 96-well plates one day prior to treatment and treated with 50 μM of H1, H3, H4, H6, Pra-H1, Pra-$_{EE1}$H6 and $_{EE2}$H6 ^{i}APC11. Cell division was monitored by live cell imaging using an ImageXpress Micro XLS wide-field screening microscope and images were acquired every 3 minutes for time courses of 24 hours. The length of mitosis was determined manually, based on the time between nuclear envelope breakdown and the onset of chromosome segregation. To stain DNA SiR-Hoechst (Spirochrome) was added in the final concentration of 50 nM one-hour prior imaging. Cells requiring more >1.5x the median mitotic duration were classified as prolonged, cells that died during mitotis according to morphological changes (extensive blebbing and implosion) were manually classified as mitotic death.

Protein Expression and Purification

Recombinant APC/C

The bacmids encoding the human APC/C were a kind gift from David Barford (MRC, Cambridge, UK).[1] APC/C expression and purification was done as it was described previously.[1] Briefly, SF9 cells (Expression Systems) were co-infected with two recombinant baculoviruses (ratio 2:5; the first corresponding to the virus containing Strep-tagged APC4) encoding the APC/C at a multiplicity of infection (MOI) of ~ 1 and a cell density of 1 million cells/ml. SF9 cells were incubated at 27 °C for 72 h. All steps of APC/C purification were performed at 4 °C. Cell pellets were re-suspended in APC/C lysis buffer (50 mM Tris-HCl pH 8.3, 250 mM NaCl, 5% Glycerol, 1 mM EDTA, 2 mM DTT, 0.1 mM PMSF, 2 mM Benzamidine, 5 units/ml benzonase, complete protease inhibitor cocktail (Roche)) and disrupted by nitrogen cavitation[2] in a 4639 Cell Disruption Vessel (Parr Instrument Company). Cell extract was cleared by centrifugation at 48 000 g at 4 °C for 1 h. Strep-tagged APC/C was captured on Strep-Tactin Superflow resin (IBA Life Sciences), washed with APC/C wash buffer (50 mM Tris-HCl pH 8, 250 mM NaCl, 5% Glycerol, 1 mM EDTA, 2 mM DTT, 2 mM Benzamidine) and eluted with Buffer E (IBA Life Sciences) supplied with additional NaCl (final concentration 250 mM), 2 mM DTT and 2 mM Benzamidine. Peak fractions were concentrated using Vivaspin 6 centrifugal concentrator (VivaProducts) and purified by size-exclusion chromatography (Superose 6 Increase 10/300 GL column; GE healthcare) in APC/C size-exclusion buffer (20 mM Hepes-NaOH pH 8, 200 mM NaCl, 2 mM DTT, 5% Glycerol). Finally, APC/C was flash-frozen in liquid nitrogen and stored at -80 °C.

Recombinant CDC20

The bacmid encoding SBP-tagged CDC20 was a kind gift from Jonathon Pines (ICR, London, UK).[3] SF9 cells (Expression Systems) were infected with the recombinant baculovirus encoding CDC20 at a multiplicity of infection (MOI) of ~1 and a cell density of 1 million cells/ml. SF9 cells were incubated at 27 °C for 72 h. All steps of CDC20 purification were performed at 4 °C. Cell pellets were re-suspended in CDC20 lysis buffer (250 mM NaCl, 50 mM Tris-HCl pH 8, 1 mM DTT, 5% Glycerol, complete protease inhibitor cocktail, 1 mM PMSF) and disrupted by nitrogen cavitation[2] in a 4639 Cell Disruption Vessel (Parr Instrument Company). Cell extract was cleared by centrifugation at 48 000 g at 4 °C for 1 h. SBP-tagged CDC20 was captured to Strep-Tactin Superflow resin, washed with 1X Buffer W (IBA Life Sciences) and eluted with Buffer E supplied with additional NaCl (final concentration 250 mM). Finally, glycerol was added to a final concentration of 10% and CDC20 was flash-frozen in liquid nitrogen and stored at -80 °C.

Other recombinant proteins

The expression and purification of recombinant securin-StrepII, N-terminal StrepII-cyclin B1 (1-86 residues), GST-UBA1, His-UBE2C^{WT}, His-UBE2C^{K119R} and His-ubiquitin was done according to established protocols as described.[4] Recombinant UBE2D1 was a kind gift of Jonathon Pines (ICR London, UK). Recombinant ubiquitin-cyclin B1 N-terminal domain (NTD) (residues 1 to 95 of cyclin B1 plus an engineered C-terminal cysteine) fusion[5] and UBE2S were a kind gift of Sonja Lorenz (MPI, Göttingen, Germany).

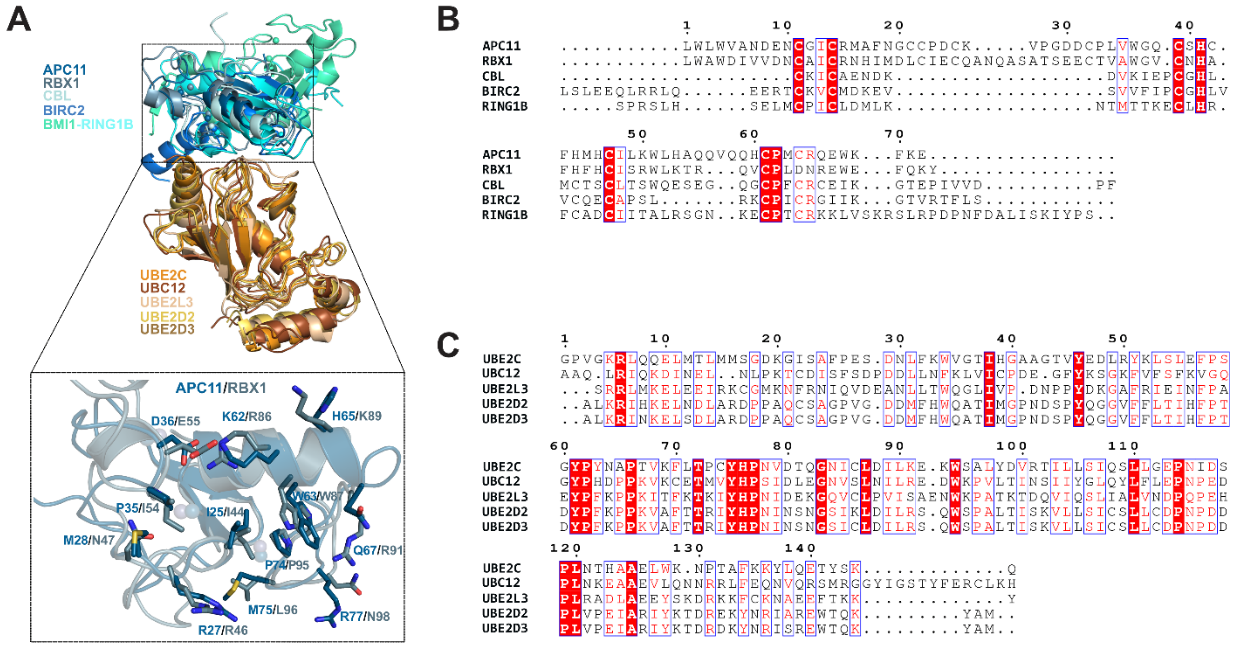

Figure S1. Superimposition of crystallographic RING/E2 complex structures and structure-based sequence alignments. (A) Cartoon representation of the superimposition of the structures of APC11/UBE2C (PDB ID 5A31, 4.3 Å), RBX1/UBC12 (PDB IDs 1LDJ, 3.0 Å and 4P5O, 3.1 Å), c-CBL/UBE2L3 (PDB ID 1FBV, 2.9 Å), BIRC2/UBE2D2 (PDB ID 6HPR, 1.7 Å) and BMI1-RING1B/UBE2D3 (PDB ID 3RPG, 2.6 Å). The conserved and semi-conserved residues of APC11 and RBX1 at their interface with E2 enzymes are shown in a zoom-in as sticks, colored by atom type and labeled. (B) Structure-based sequence alignment of selected RING protein domains. Conserved and semi-conserved residues are marked with a blue box and labeled in white with red background and red with white background, respectively. (C) Structure-based sequence alignment of E2 enzymes. Figures created with PyMOL v.2.4.1 and ESPript.

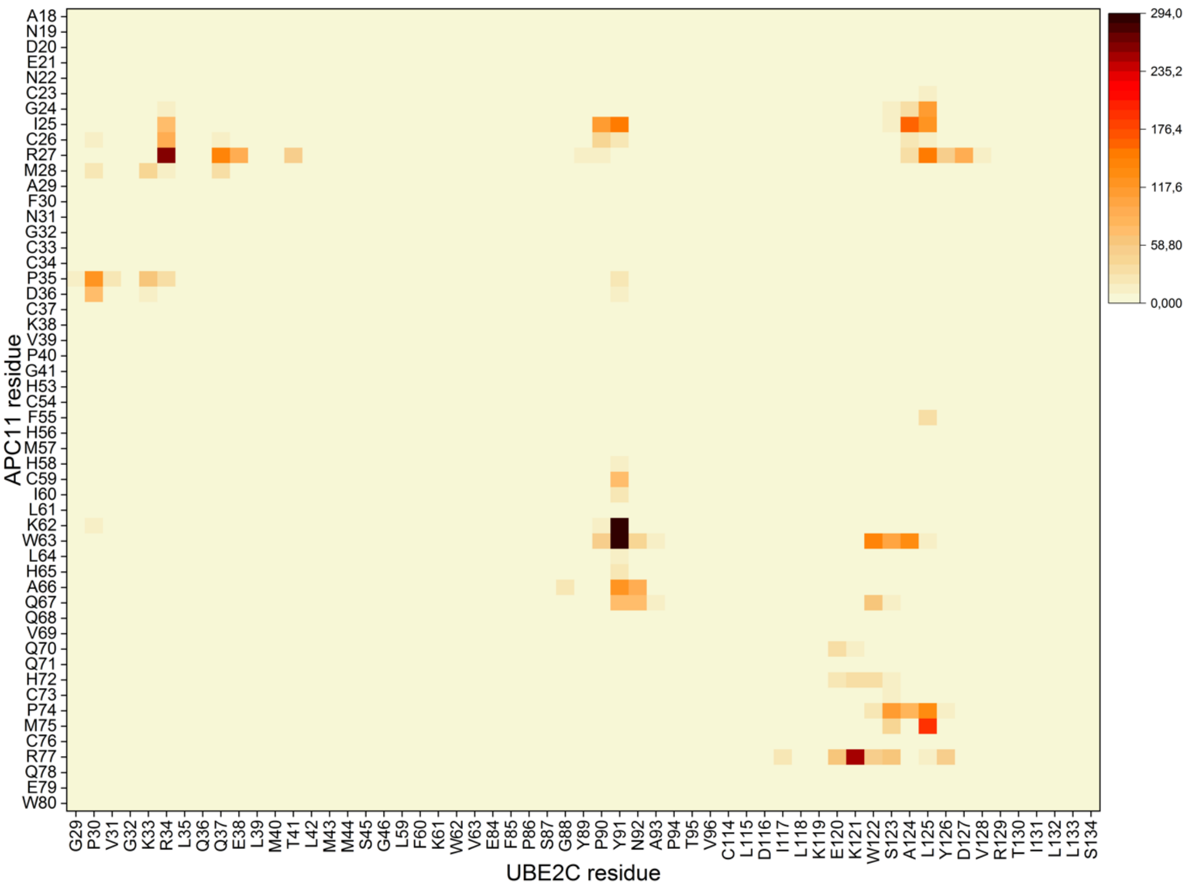

Figure S2. APC11 recognition by UBE2C. MD-based contact map of the APC11/UBE2C complex. Plot generated with data extracted from three independent MD simulations. The number of contacts corresponds to the sum of each individual atom pair within their respective residues.

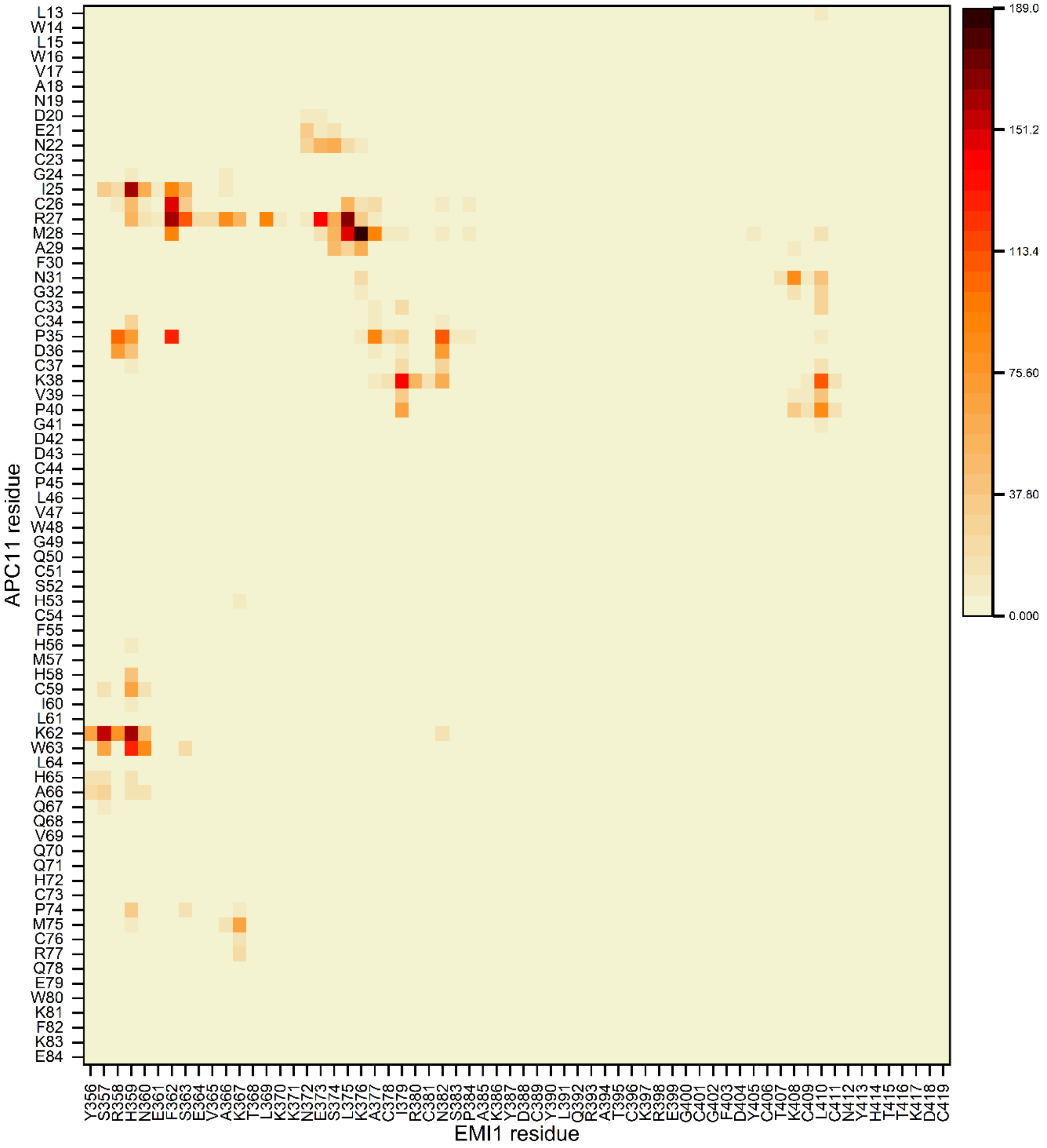

Figure S3. APC11 recognition by the naturally occurring inhibitor EMI1. MD-based contact map of the APC11/EMI1 complex. Plot generated with data extracted from three independent MD simulations. The number of contacts corresponds to the sum of each individual atom pair within their respective residues.

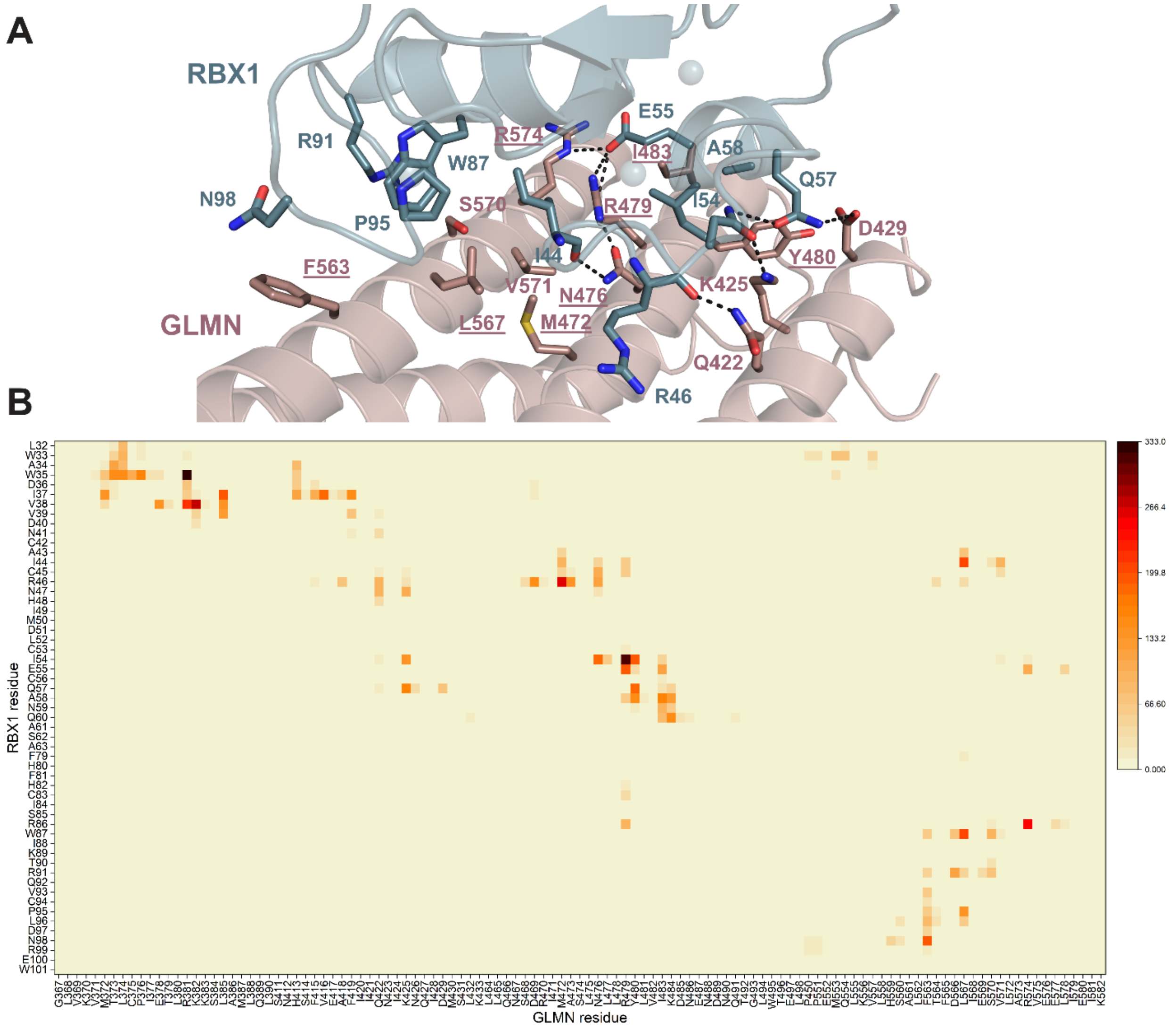

Figure S4. RBX1 recognition by the naturally occurring inhibitor GLMN. (A) Cartoon representation of the RBX1/GLMN complex structure (PDB ID 4F52, 3.0 Å) with relevant interacting residues shown in sticks, colored by atom type and labeled, with the strongest contributors in binding underlined. Figure created with PyMOL v.2.4.1. (B) MD-based contact map of the RBX1/GLMN complex. Plot generated with data extracted from three independent MD simulations. The number of contacts corresponds to the sum of each individual atom pair within their respective residues.

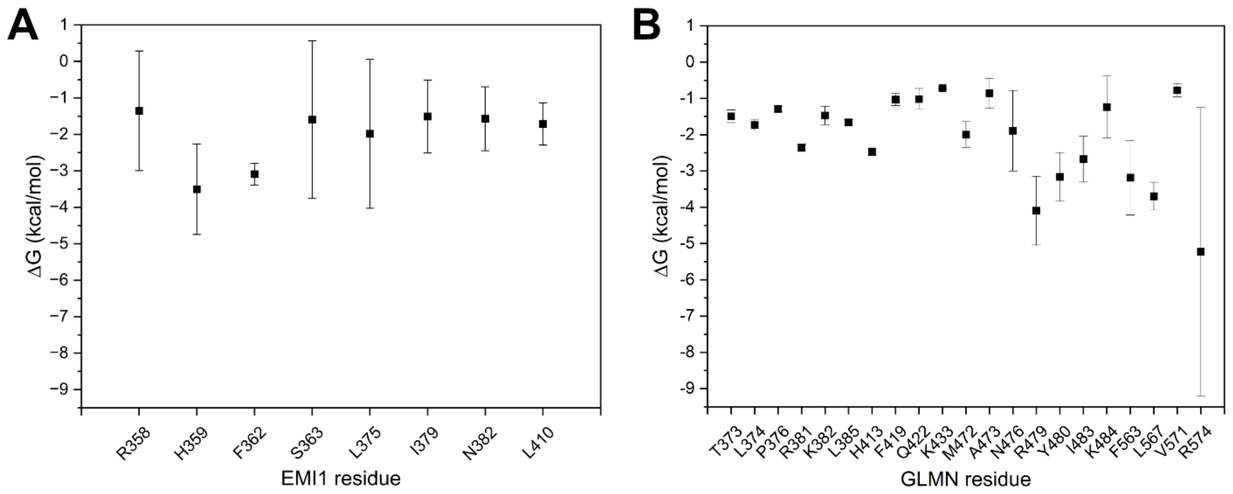

Figure S5. Energetic contribution of naturally occurring inhibitors recognized by RING proteins. Per-residue binding energy contribution calculated with MM-GBSA from three independent MD simulations of (A) EMI1 in complex with APC11 and (B) GLMN in complex with RBX1.

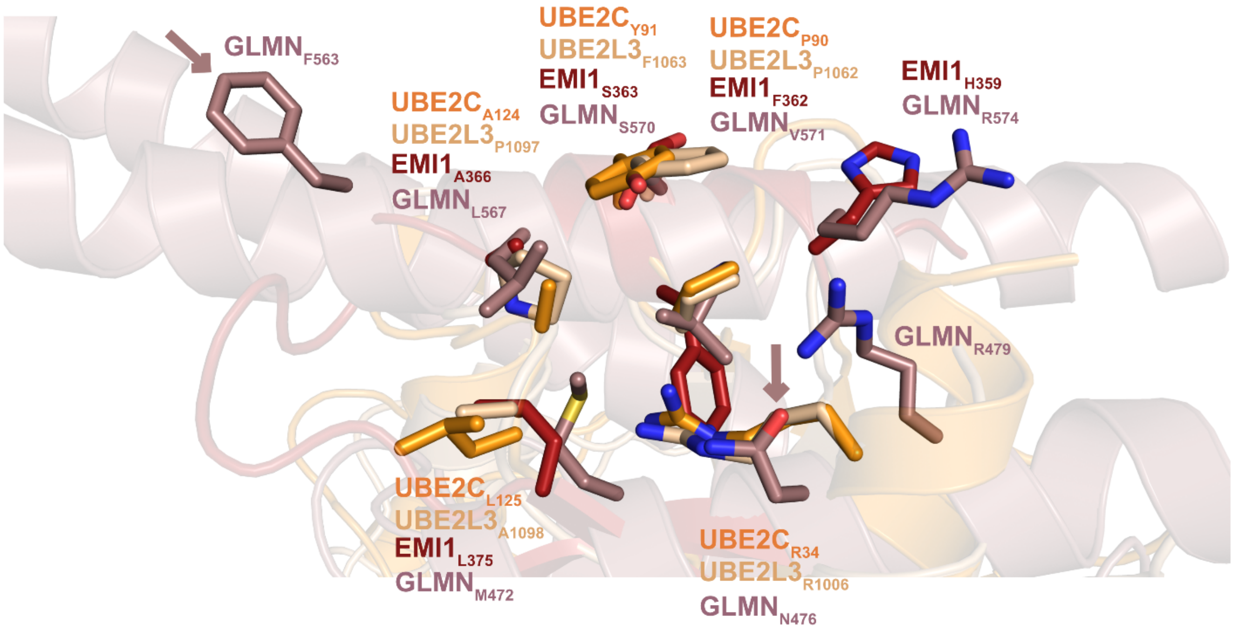

Figure S6. Atomic equivalences in RING recognition among E2 enzymes and naturally occurring inhibitors. Superimposition of the crystallographic structures of UBE2C (PDB ID 5A31, 4.3 Å, orange), UBE2L3 (PDB ID 1FBV, 2.9 Å, pale), EMI1 (PDB ID 4UI9, 3.6 Å, dark red) and GLMN (PDB ID 4F52, 3.0 Å, brown). Proteins are shown as cartoons with relevant residues in sticks, colored by atom type and labeled. Arrows indicate positions for incorporation of additional functionalities in the RING selectivity design strategy. For clarity, cartoon representations are shown with transparency. Figure created with PyMOL v.2.4.1.

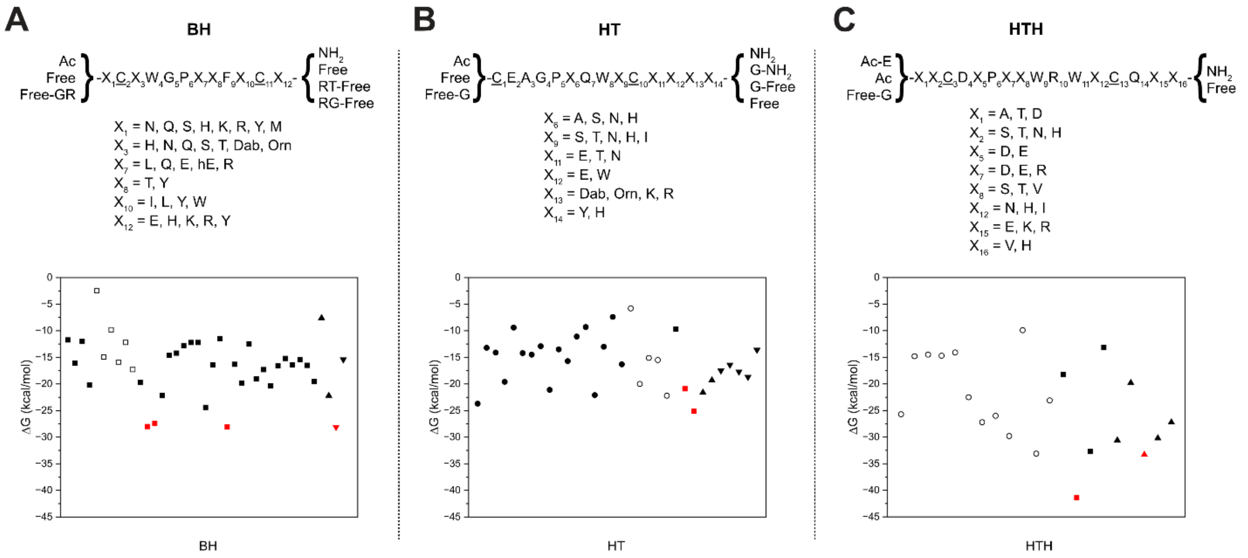

Figure S7. *De novo* designed E2 mimetics based on BH, HT and HTH scaffolds (TOP) and their binding free energies to APC11 (BOTTOM). *De novo* design of (A) 39 BH (B) 32 HT and (C) 21 HTH molecules. Proteogenic amino acids are indicated by their single-letter code and unnatural amino acids as: Dab (2,4-diaminobutyric acid), Orn (ornithine) and hE (homoglutamic acid). Underlined cysteine residues denote disulfide bridges. Selected *N*- and *C*-term functionalizations are listed. Binding free energy was calculated with MM-GBSA. (■) 12 residue-long BH with acetylated *N*-term and amidated *C*-term. (▲) 13 residue-long BH with free *N*-term and amidated *C*-term. (□ and ▼) 12 and 14 residue-long BH with free *N*-term and *C*-term, respectively. (● and ○) 15 and 14 residue-long HT with free *N*-term and amidated *C*-term, respectively. (■) 14 residue-long HT with acetylated *N*-term and amidated *C*-term. (▲ and ▼) 16 and 15 residue-long HT with free *N*-term and *C*-term, respectively. (○ and ■) 17 and 16 residue-long HTH with acetylated *N*-term and amidated *C*-term, respectively. (▲) 17 residue-long HTH with free *N*-term and *C*-term. Squares and triangles in red indicate the selected molecules for further investigation.

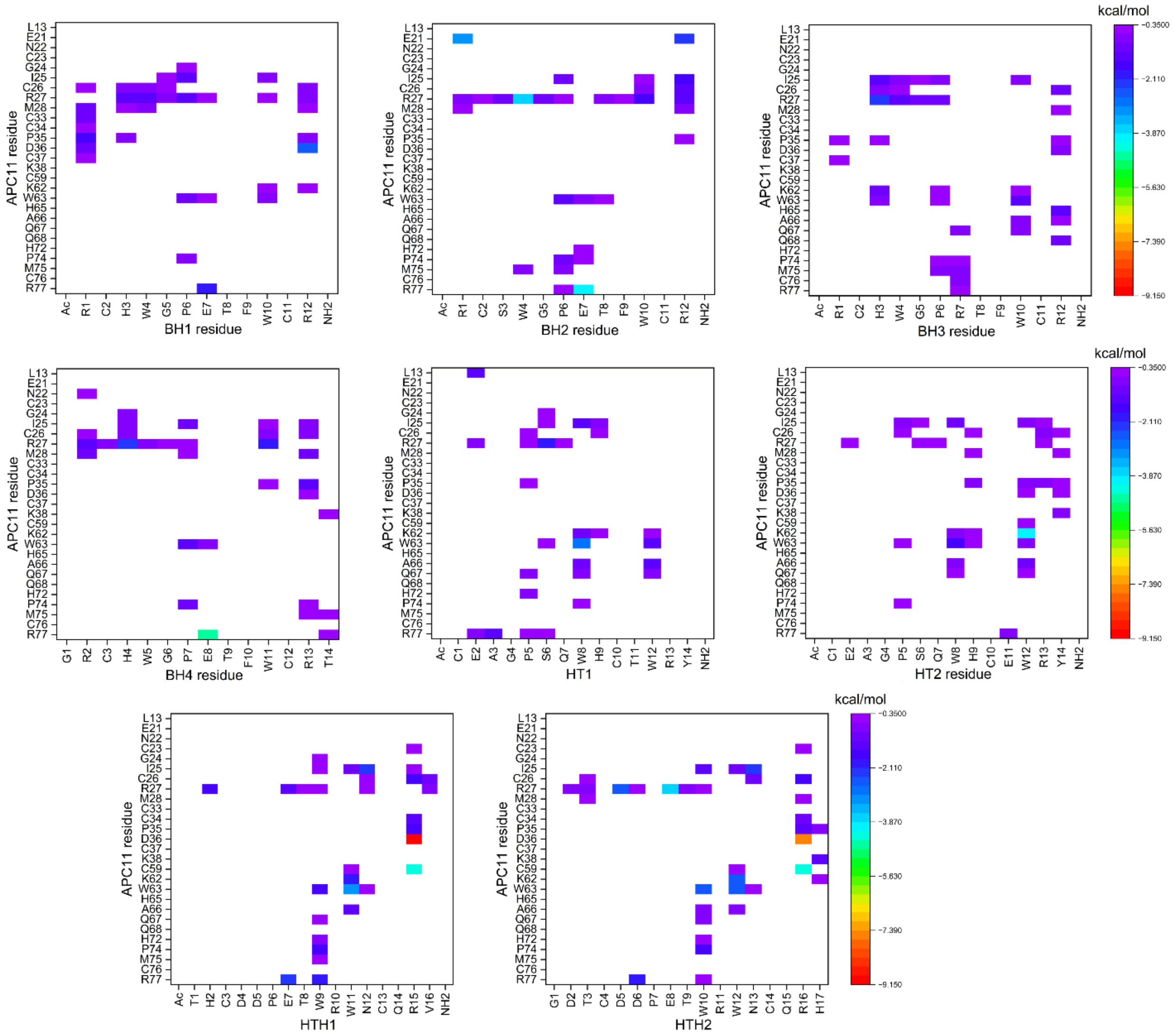

Figure S8. Pairwise binding energy contribution calculated with MM-GBSA from three independent MD simulations of APC11 in complex with the *de novo* designed BH, HT and HTH molecules. Mean values are indicated by the gradient-colored side bar.

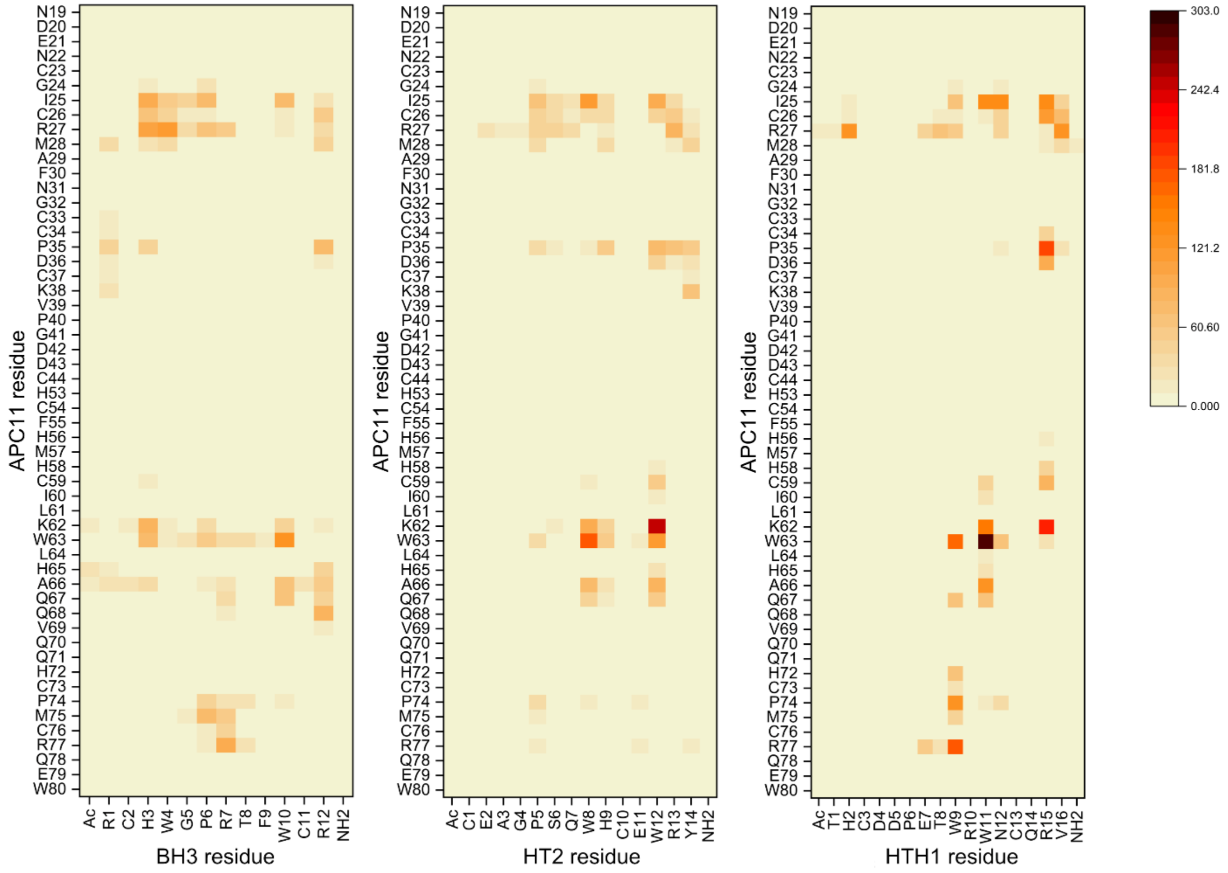

Figure S9. APC11 recognition by *de novo* designed BH, HT and HTH molecules. MD-based contact maps of the *de novo* designed molecules in complex with APC11. Plot generated with data extracted from three independent MD simulations. The number of contacts corresponds to the sum of each individual atom pair within their respective residues.

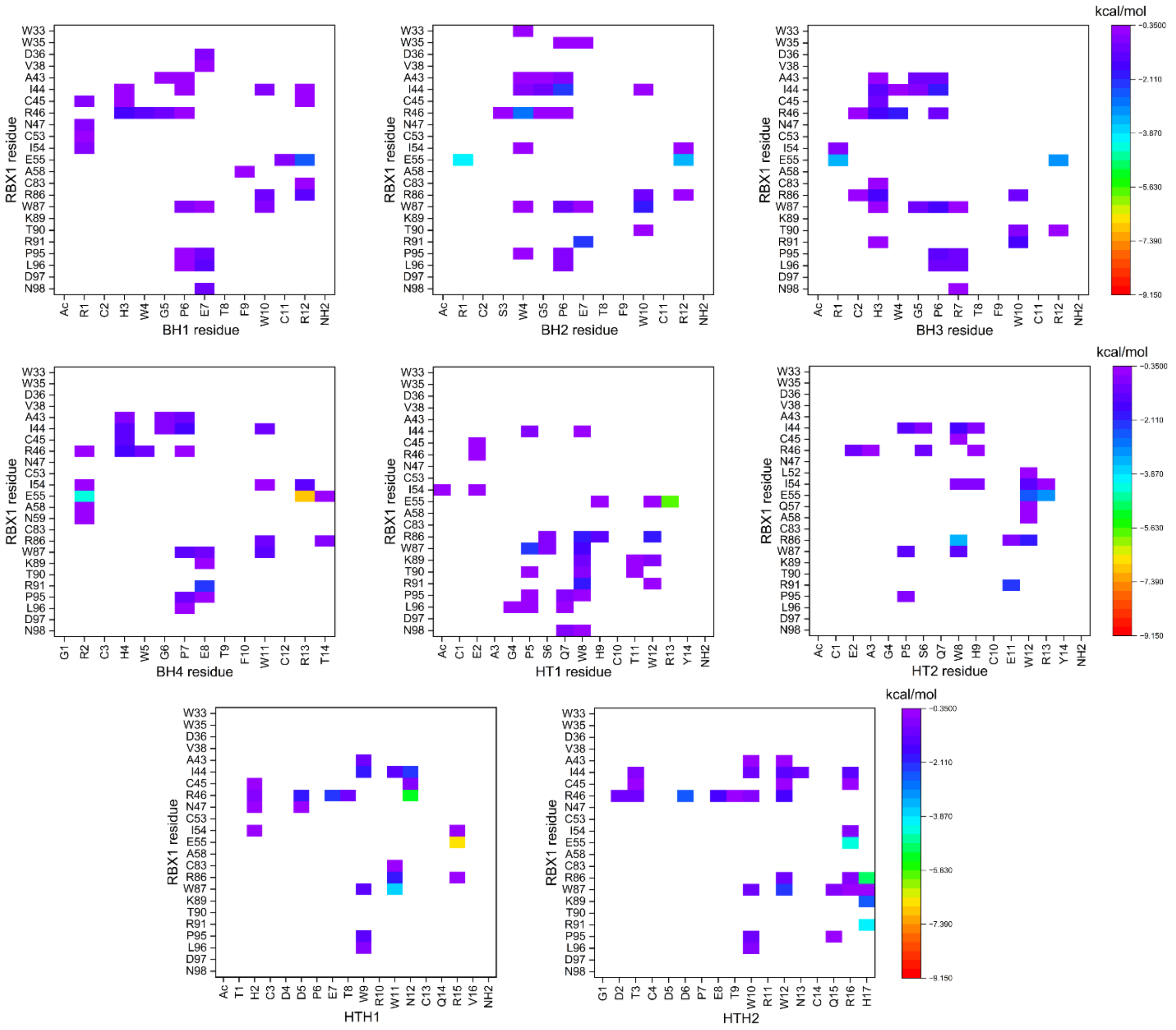

Figure S10. Pairwise binding energy contribution calculated with MM-GBSA from three independent MD simulations of RBX1 in complex with the *de novo* designed BH, HT and HTH molecules. Mean values are indicated by the gradient-colored side bar.

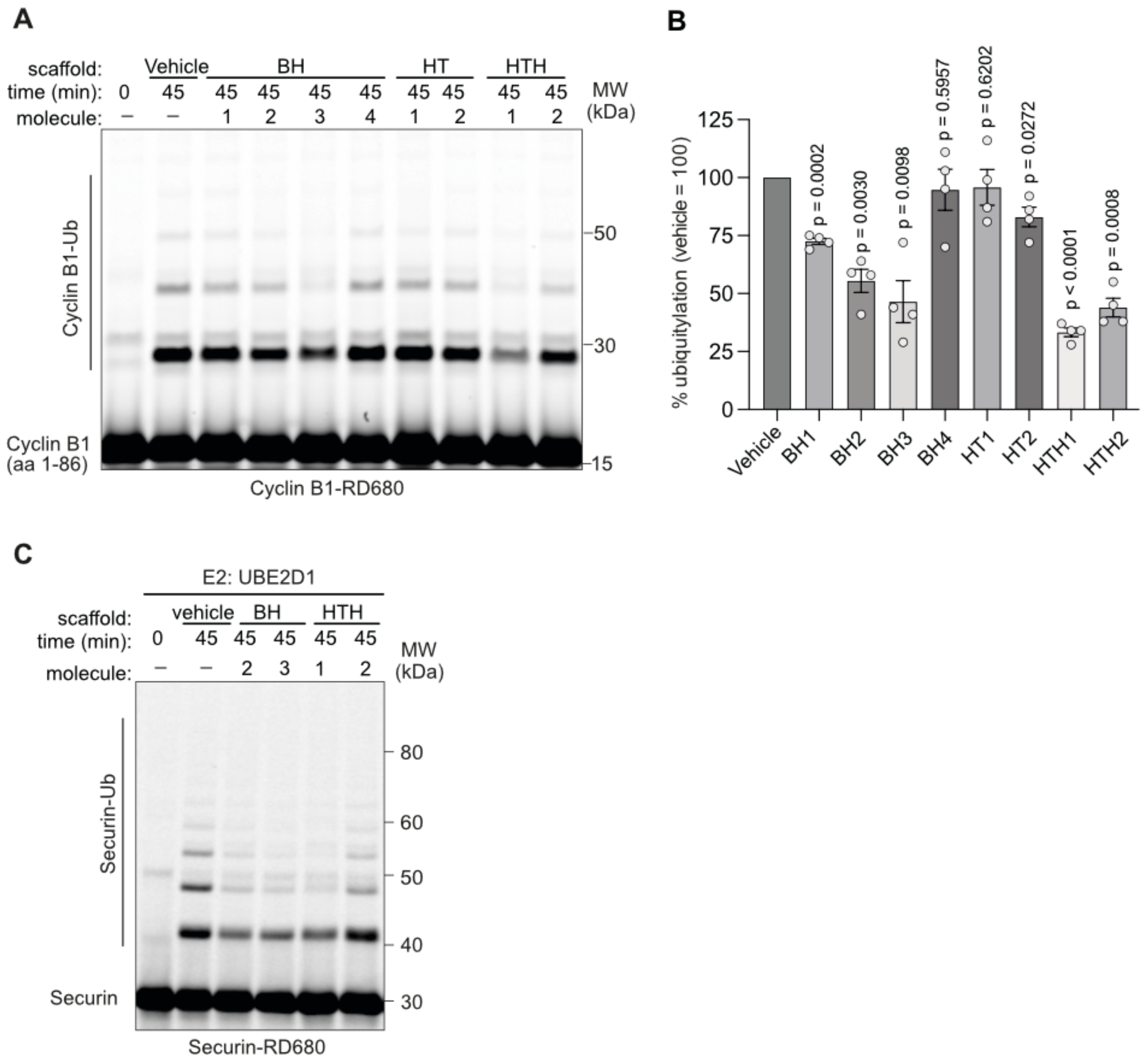

Figure S11. E2 mimetics inhibit the APC/C in vitro. (A) In-gel scan showing the covalent linkage of ubiquitin (Ub) to the IR-dye RD680 labeled N terminus of cyclin B1 during in vitro APC/C activity assays as in (Figure 3B,C) in the presence of 100 µM E2 mimetics or vehicle. (B) Quantification of (A). Bars showing the mean ± SEM. Significance according to 1-sided t-test compared to vehicle=100%, n=5. (C) Representative in-gel scan showing the covalent linkage of methylated Ub to securin with the best BH and HTH molecules.

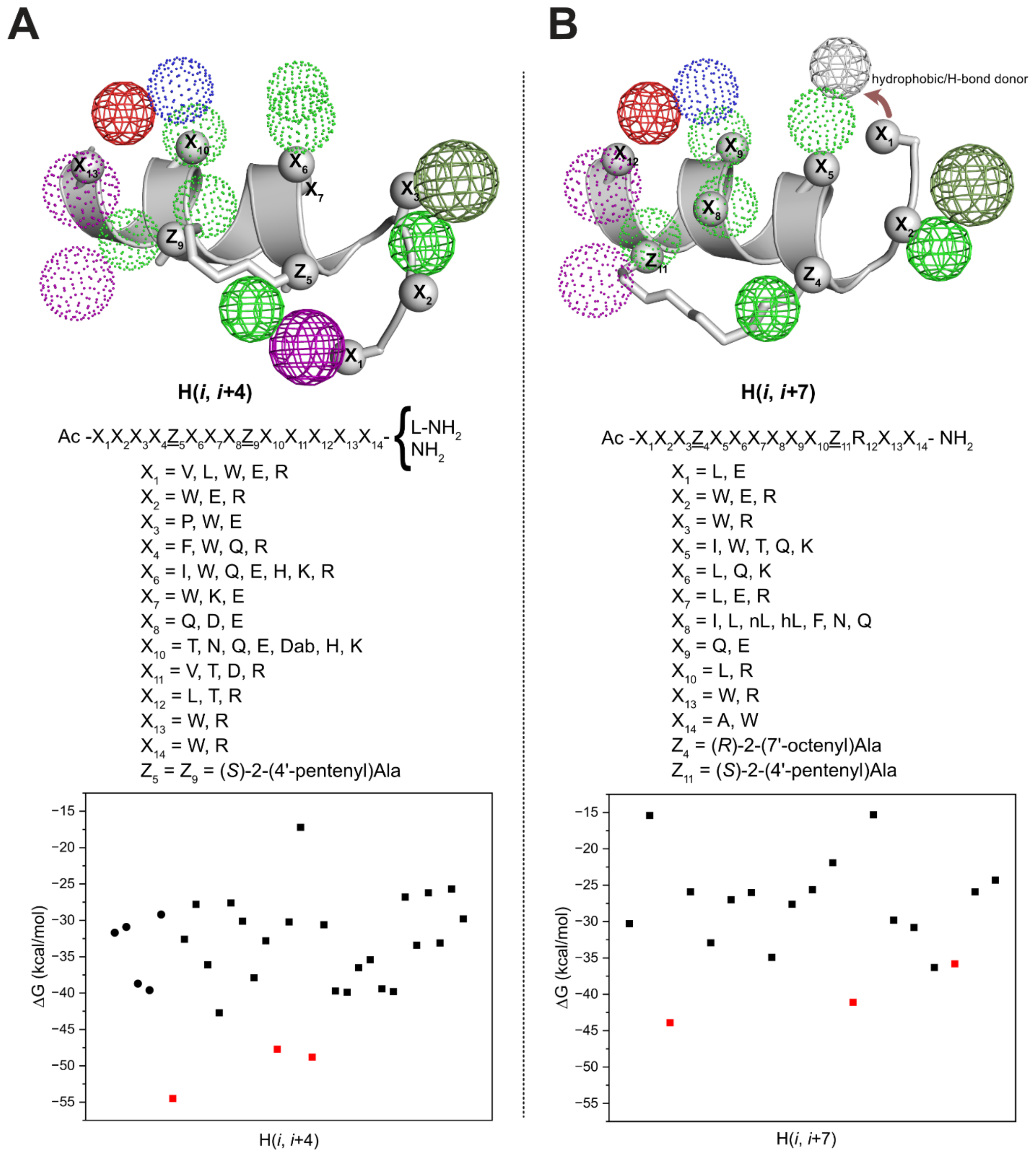

Figure S12. RING selectivity approach (TOP), *de novo* designed [i]APC11 based on H(*i*, *i*+4) and H(*i*, *i*+7) scaffolds (MIDDLE) and their binding free energies to APC11 (BOTTOM). Selectivity approach towards APC11 recognition through an expanded pharmacophore into helical (H) scaffolds. Previously incorporated and newly designed pharmacophore features are indicated by dots and mesh, respectively (aromatic, hydrophobic, H-bond acceptor, H-bond donor, cation/donor and exchanged properties in smudge green, green, red, blue, purple and grey, respectively). *De novo* design of (A) 31 H(*i*, *i*+4) and (B) 19 H(*i*, *i*+7) molecules. Figure created with PyMOL v.2.4.1. Proteogenic amino acids are indicated by their single-letter

code. Unnatural amino acids are denoted as follows: Dab (2,4-diaminobutyric acid), nL (norleucine) and hL (homoleucine). Underlined unnatural amino acids denote a hydrocarbon staple. Selected *C*-term functionalizations in H(*i*, *i*+4) designed molecules are listed. Binding free energy was calculated with MM-GBSA. H (*i*, *i*+4) molecules of 14- and 15-residues length with *N*-term acetylated and *C*-term amidated are represented by ■ and ●, respectively. H(*i*, *i*+7) of 14-residues length with *N*-term acetylated and *C*-term amidated are represented by ■. Squares in red indicate the selected molecules for further investigation.

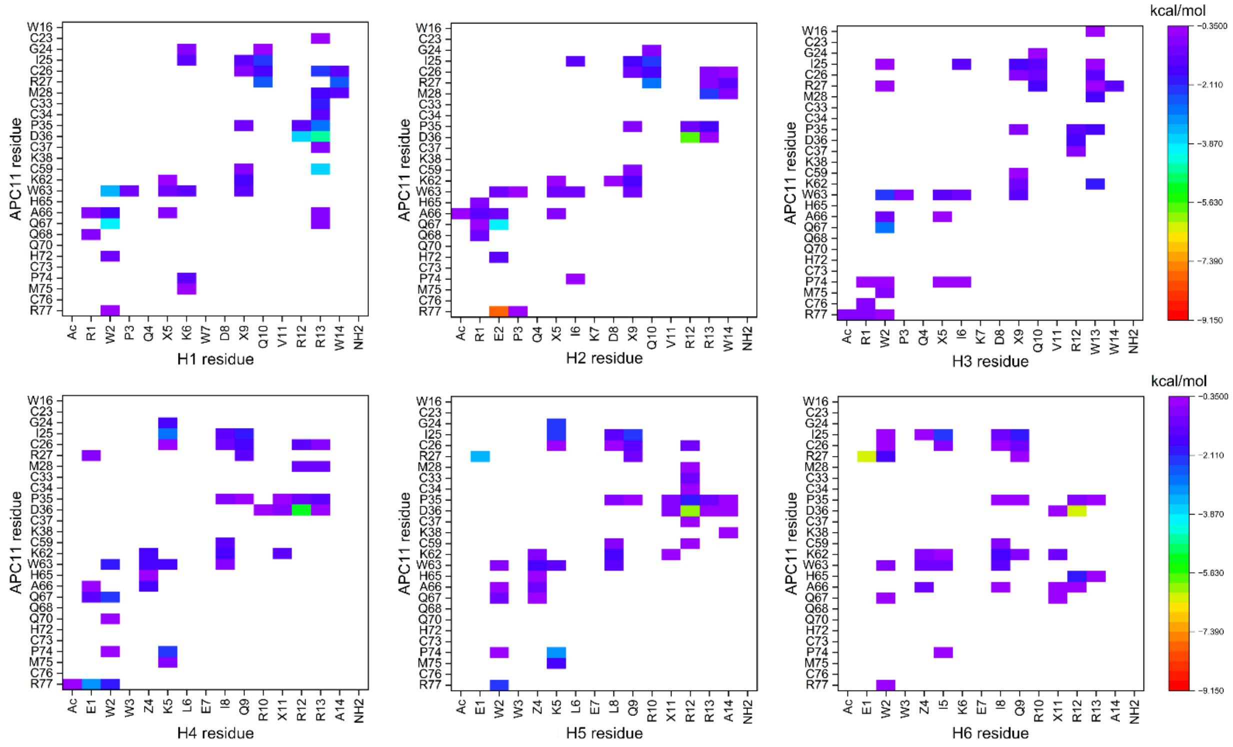

Figure S13. Pairwise binding energy contribution calculated with MM-GBSA from three independent MD simulations of APC11 in complex with the *de novo* designed H molecules. Mean values are indicated by the gradient-colored side bar.

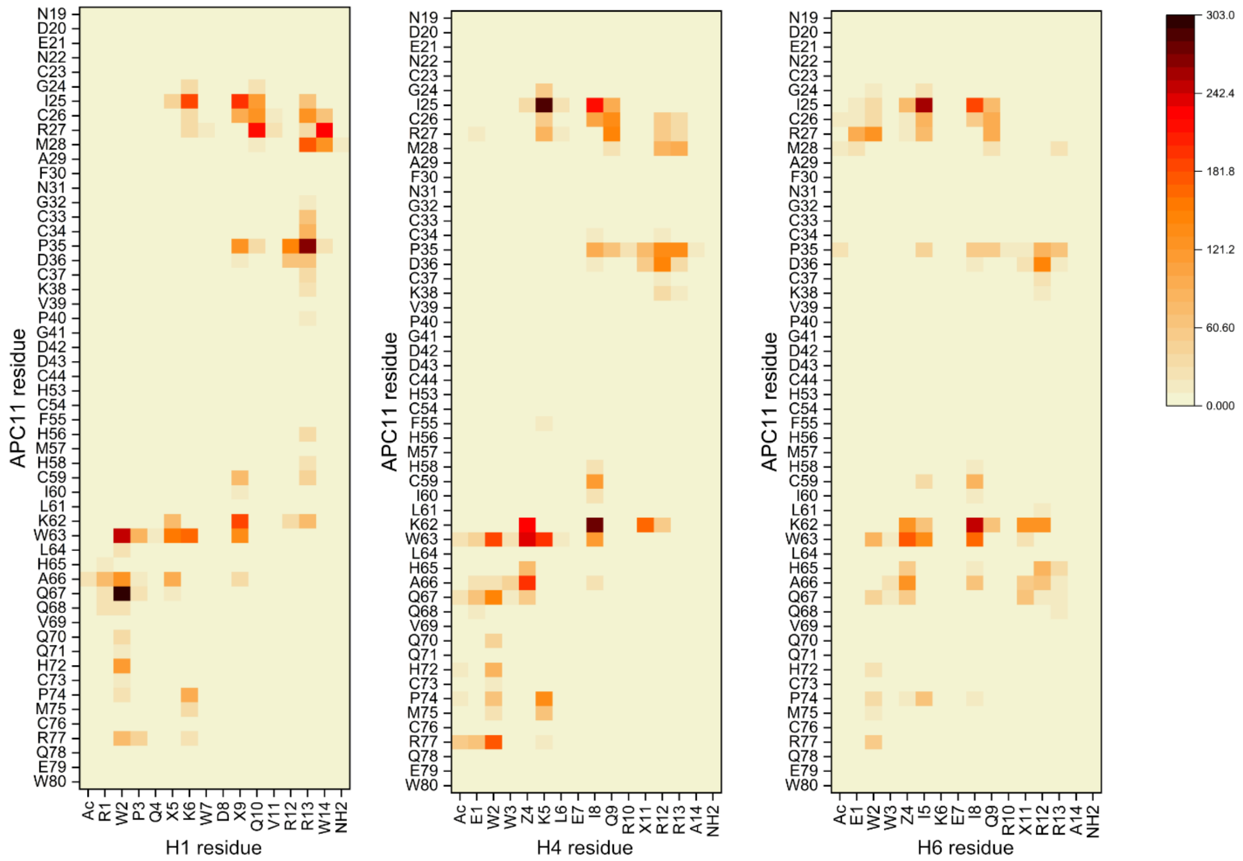

Figure S14. APC11 recognition by *de novo* designed H molecules. MD-based contact maps of the *de novo* designed H molecules in complex with APC11. Plot generated with data extracted from three independent MD simulations. The number of contacts corresponds to the sum of each individual atom pair within their respective residues.

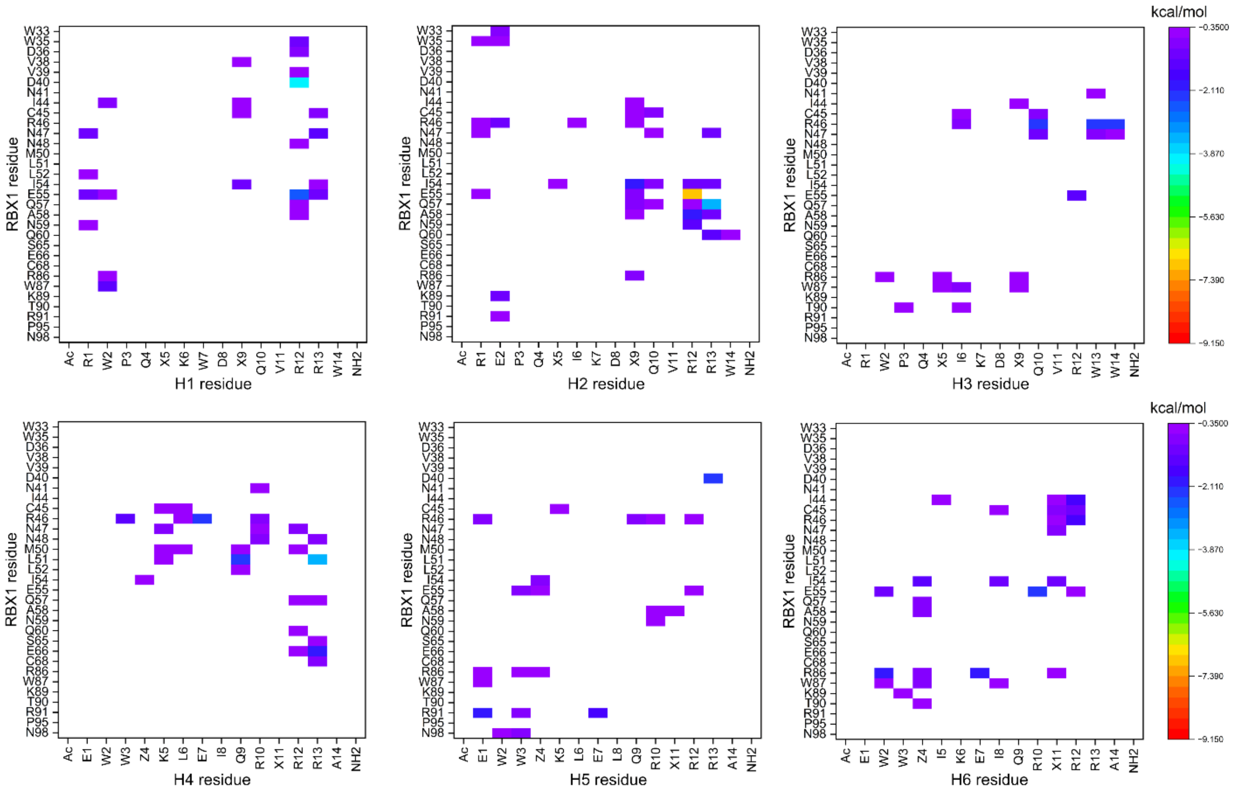

Figure S15. Pairwise binding energy contribution calculated with MM-GBSA from three independent MD simulations of RBX1 in complex with the *de novo* designed H molecules. Mean values are indicated by the gradient-colored side bar.

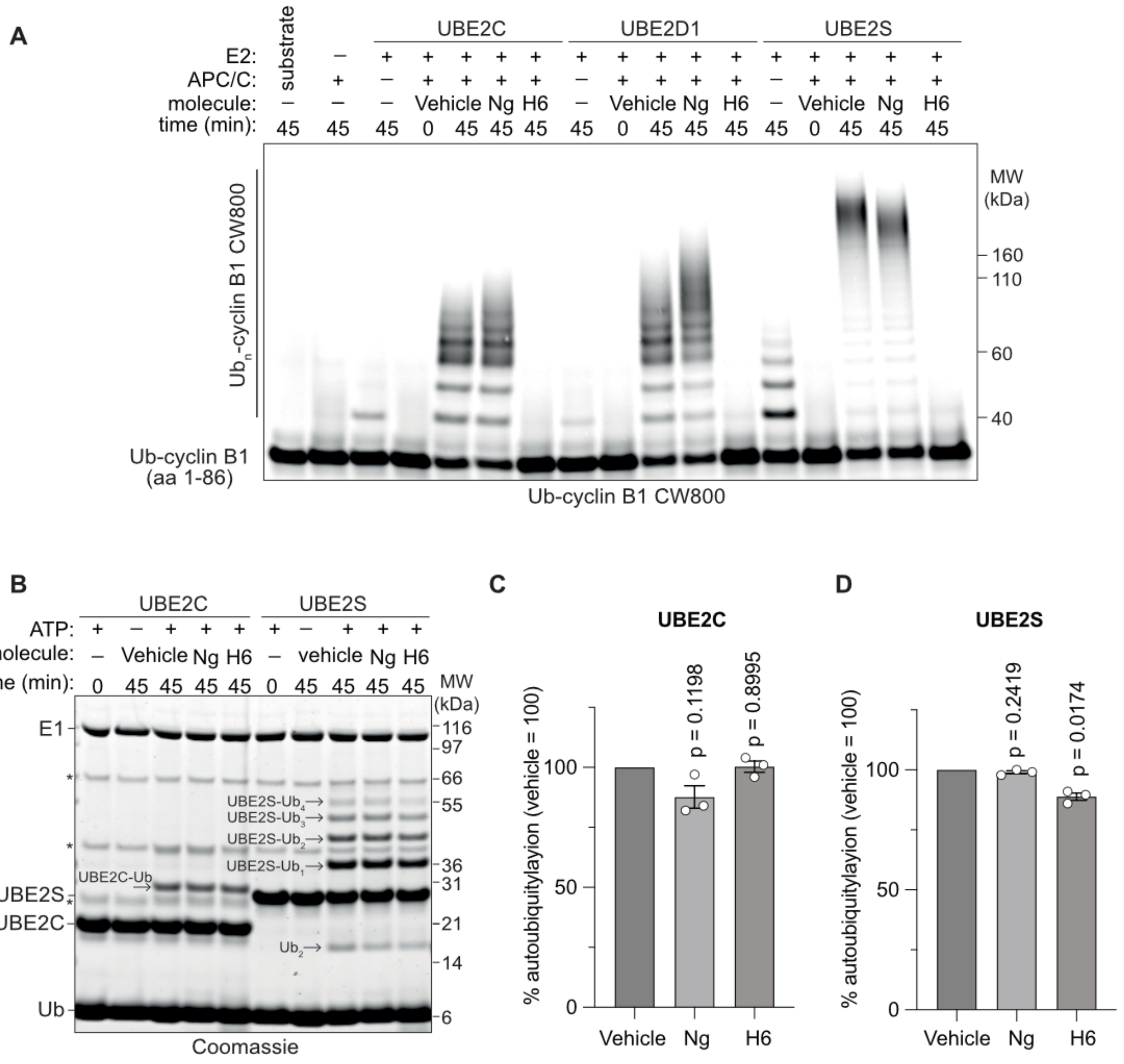

Figure S16. ^{i}APC11 inhibits the APC/C in an E2-independent manner *in vitro.* (A) In-gel scan showing the covalent linkage of ubiquitin (Ub) to the IR-dye CW800 labeled Ub-cyclin B1 during in vitro APC/C activity assays as in (Fig. 3A, B) in the presence of 100 µM H6 and Ng negative control molecules. Note, to enable UBE2S-driven substrate ubiquitylation without a priming E2, ubiquitin was fused to the N terminus of cyclin B1 (residues 1-86).[5] (B) Coomassie gel scan showing the autoubiquitylation of UBE2C and UBE2S to assess the impact of ^{i}APC11 molecules on E2 activity. Autoubiquitylated E2 as well as di-Ub molecules (UBE2S) are highlighted by arrows. Reaction supplements and impurities are labeled with asterisks. (C and D) Quantification of autoubiquitylated E2 species from (B). Bars showing the mean ± SEM. Significance according to 1-sided t-test compared to vehicle=100%, n=3.

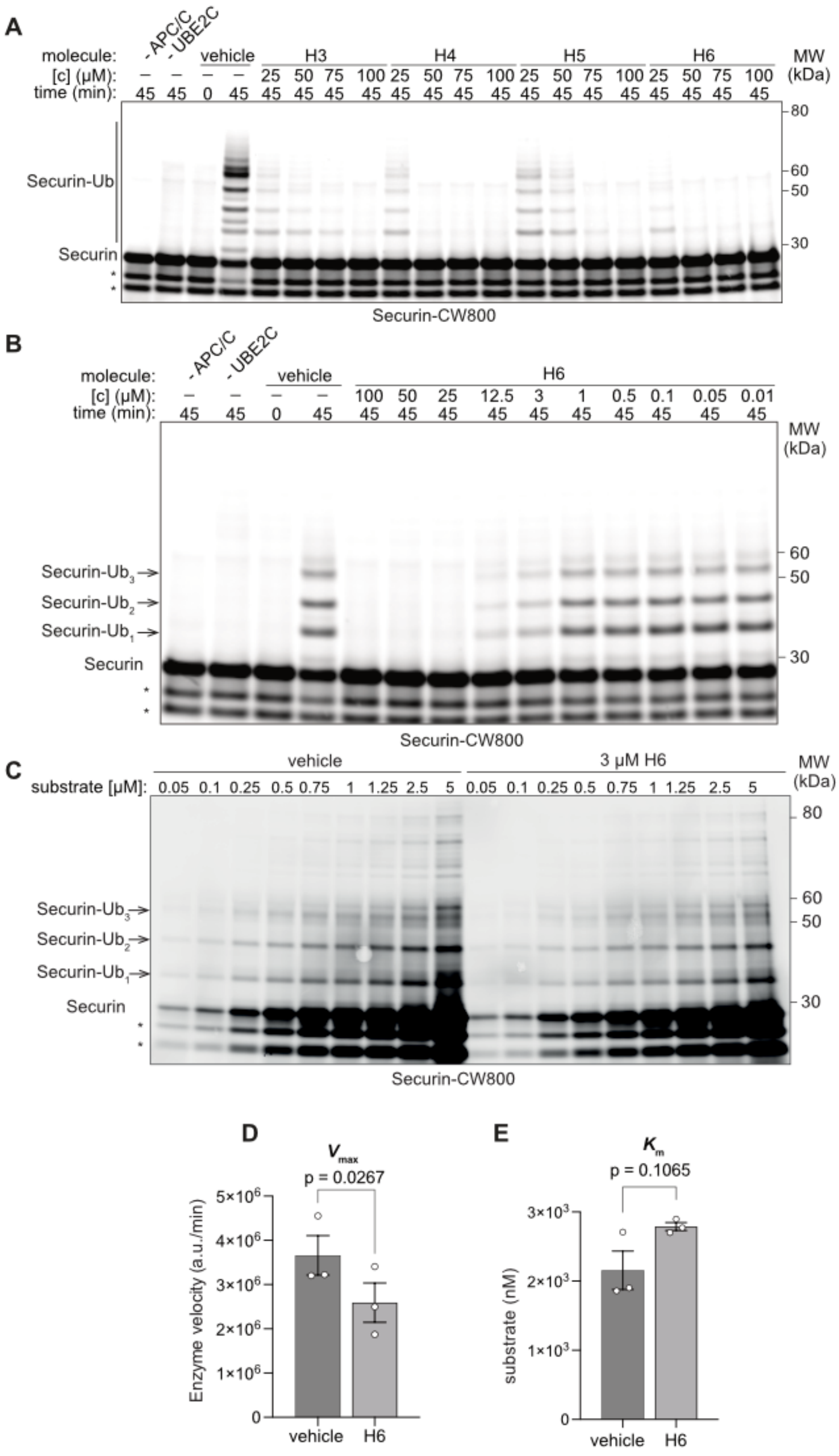
A
molecule:
- APC/C
- UBE2C
vehicle
H3
H4
H5
H6
MW (kDa)
[c] (µM):
25 50 75 100 25 50 75 100 25 50 75 100 25 50 75 100
time (min):
45 45 0 45 45 45 45 45 45 45 45 45 45 45 45 45 45 45 45 45
Securin-Ub
Securin
80
60
50
30
Securin-CW800
B
molecule:
- APC/C
- UBE2C
vehicle
H6
[c] (µM):
100 50 25 12.5 3 1 0.5 0.1 0.05 0.01
time (min):
45 45 0 45 45 45 45 45 45 45 45 45 45 45
MW (kDa)
Securin-Ub3
Securin-Ub2
Securin-Ub1
Securin
60
50
30
Securin-CW800
C
vehicle
3 µM H6
substrate [µM]:
0.05 0.1 0.25 0.5 0.75 1 1.25 2.5 5 0.05 0.1 0.25 0.5 0.75 1 1.25 2.5 5
MW (kDa)
Securin-Ub3
Securin-Ub2
Securin-Ub1
Securin
80
60
50
30
Securin-CW800
D
Vmax
p = 0.0267
Enzyme velocity (a.u./min)
5×10^6
4×10^6
3×10^6
2×10^6
1×10^6
0
vehicle
H6
E
Km
p = 0.1065
substrate (nM)
3×10^3
2×10^3
1×10^3
0
vehicle
H6

Figure S17. IC_{50} and Michaelis-Menten Kinetics of ^{i}APC11. (A) In-gel scan showing the covalent linkage of ubiquitin (Ub) to IR-dye CW800 labeled securin during *in vitro* APC/C activity assays with a titration of ^{i}APC11 molecules ranging from 25 - 100 µM, n=3. Quantifications are shown in Figure 6C. Asterisks indicate truncated forms of securin. (B) In-gel scan showing the covalent linkage of ubiquitin (Ub) to IR-dye CW800 labeled securin during. In vitro APC/C activity assays with increasing concentrations of H6 molecules. Ubiquitylated securin species quantified for IC_{50} determination in Figure 6D are highlighted by arrows. Asterisks indicate truncated forms of securin. (C) In-gel scan showing the covalent linkage of ubiquitin (Ub) to securin during 2 minutes (initial velocity conditions) *in vitro* APC/C activity assays with increasing concentrations of IR-dye CW800 labeled securin. Ubiquitylated securin species quantified for Michaelis-Menten and K_i calculations in Figure 6E are highlighted by arrows. Asterisks indicate truncated forms of securin. (D and E) Bars showing the mean ± SEM. of V_{max} and K_m determined from data shown in Figure 6E and panel (C). Significance according paired, two-tailed t-test, n=3.

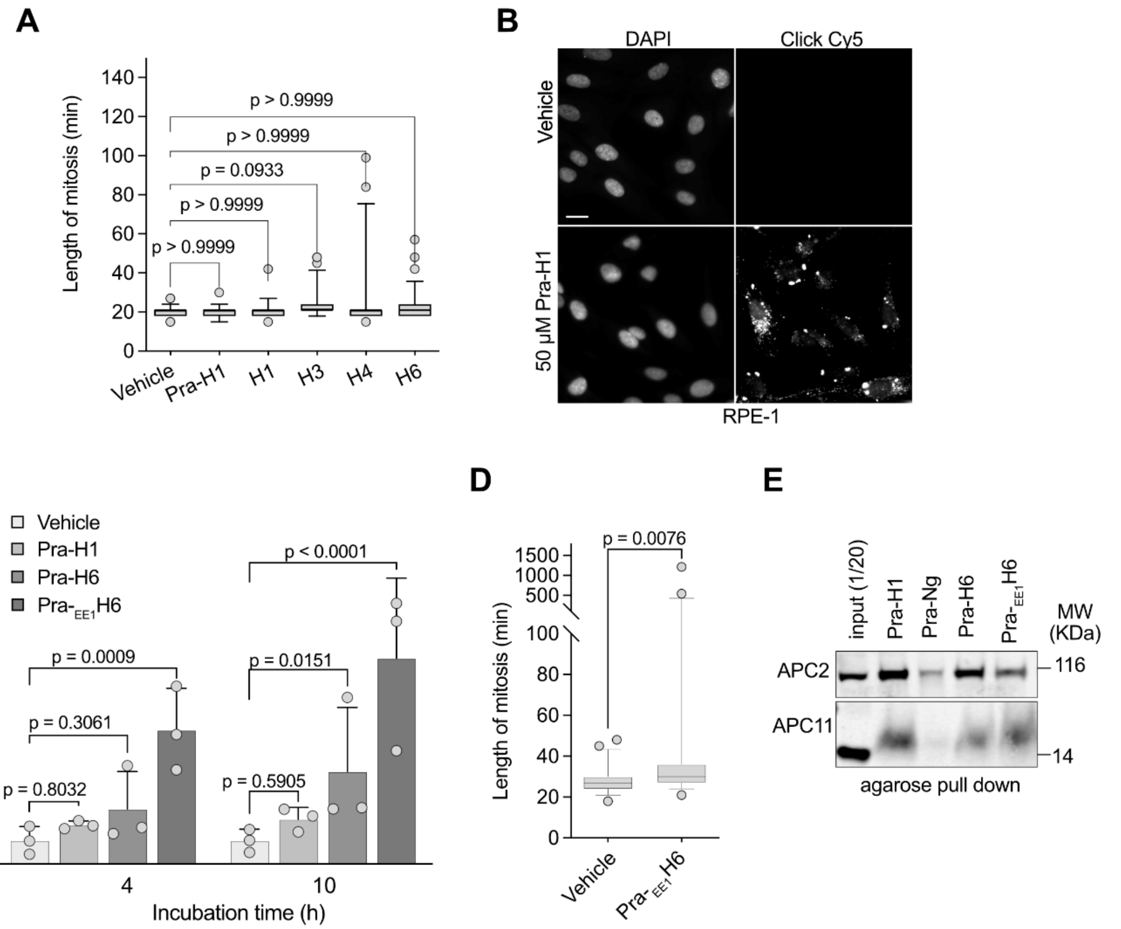

Figure S18. Localization and inhibition of ^{i}APC11. (A) Boxplots indicating the length of mitosis in RPE-1 cells from nuclear envelope breakdown to anaphase determined by live cell microscopy from single cells between 10 and 24 hours of treatment with 50 µM ^{i}APC11. Box show the median, 25th, 75th percentiles, whiskers the 5-95% percentiles. Significance according to Kruskal-Wallis multi-comparisons test with Dunn's correction, n=2. (B) Micrographs showing the localisation of Pra-H1 in RPE-1 cells after 10 hours treatment with 50 µM inhibitor or vehicle and subsequent fixation and clicking to the dye AF488. Note, Pra-H1 is largely localized in a punctate pattern outside of the nucleus. Scale bar is 20 µm. (C) Quantification nuclear localized ^{i}APC11 in HeLa cells after 4- or 10-hours treatment with 50 µM inhibitors or vehicle by measuring the nuclear intensity of Cy5 dye clicked to the Pra-group of the ^{i}APC11 from images as shown in Figure 7C. Bars showing the mean ± SD. Significance according to multi-comparison two-way ANOVA with Dunnet's correction, n=3. (D) Boxplots indicating the length of mitosis in HeLa cells from nuclear envelope breakdown to anaphase quantified from single cells between 10 and 24 hours of 50 µM ^{i}APC11 treatment determined by live cell microscopy. Box show the median, 25th, 75th percentiles, whiskers the 5-95% percentiles. Significance according to Kruskal-Wallis multi-comparisons test with Dunn's correction, n=2 (technical repeats). (E) Western blot detecting APC11 and APC2 in pull down experiments from asynchronous HeLa using the indicated inhibitors clicked to azide agarose beads. Pra-Ng (Pra-Ng Ac-RRPraZ̲AQERARX̲VRY-NH_2, Z refers to (*R*)-2-(7'-octenyl)Ala, X refers to (*S*)-2-(4'-pentenyl)Ala, and underlined Z̲ and X̲ denote a hydrocarbon-staple) is a negative control to monitor background binding.

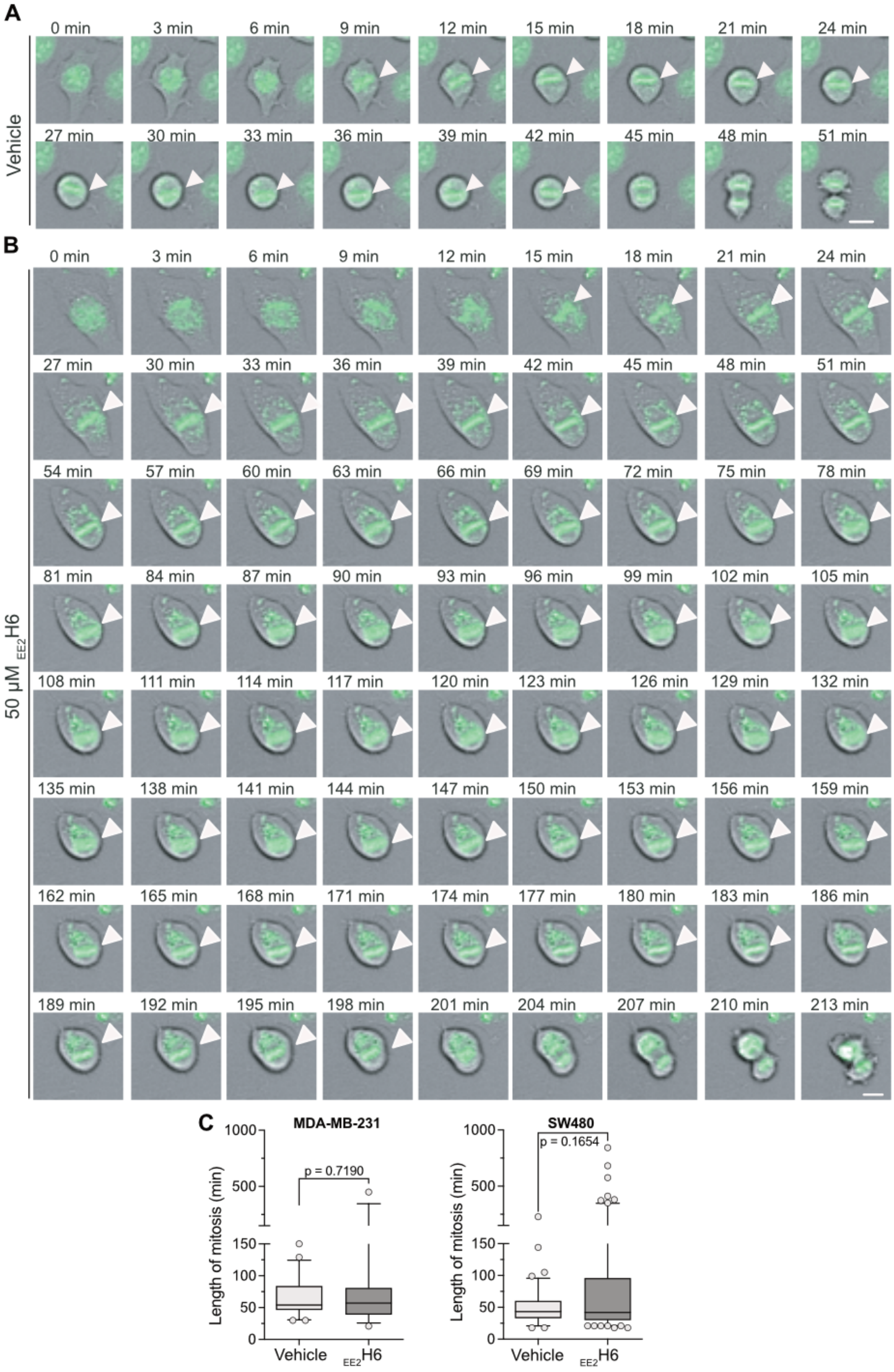
A
0 min 3 min 6 min 9 min 12 min 15 min 18 min 21 min 24 min
27 min 30 min 33 min 36 min 39 min 42 min 45 min 48 min 51 min
Vehicle
B
0 min 3 min 6 min 9 min 12 min 15 min 18 min 21 min 24 min
27 min 30 min 33 min 36 min 39 min 42 min 45 min 48 min 51 min
54 min 57 min 60 min 63 min 66 min 69 min 72 min 75 min 78 min
81 min 84 min 87 min 90 min 93 min 96 min 99 min 102 min 105 min
108 min 111 min 114 min 117 min 120 min 123 min 126 min 129 min 132 min
135 min 138 min 141 min 144 min 147 min 150 min 153 min 156 min 159 min
162 min 165 min 168 min 171 min 174 min 177 min 180 min 183 min 186 min
189 min 192 min 195 min 198 min 201 min 204 min 207 min 210 min 213 min
50 µM EE2H6
C
MDA-MB-231
SW480
p = 0.7190
p = 0.1654
Length of mitosis (min)
1000
500
150
100
50
0
Vehicle
EE2H6

Figure S19. Time lapse imaging of $_{EE2}$H6 ^{i}APC11 treatment and non-responsive cancer cells. (A and B) Representative micrographs showing the overlay of bright-field and DNA live cell dye Sir-Hoechst (green) of a vehicle (A) or 50 µM $_{EE2}$H6 treated (B) HeLa cell. Note, $_{EE2}$H6 treated cells arrest for a prolonged time with a metaphase plate (white triangle) indicative of APC/C inhibition. Scale bars are 20 µm. (C) Boxplots indicating the length of mitosis from nuclear envelope breakdown to anaphase in MDA-MB-231 breast and SW480 colon cancer cells determined by live cell microscopy from single cells between 10 and 24 hours of treatment with 50 µM $_{EE2}$H6. Box show the median, 25th, 75th percentiles, whiskers the 5-95% percentiles. Significance according to two-tailed Mann-Whitney test, n=2. Note, unlike the cancer cell lines presented in Figure 8, these two cell lines show no response to $_{EE2}$H6 treatment.

Table S1. Binding free energies of RING proteins in complex with naturally occurring inhibitors and UBE2C. Energies were obtained with MM-GBSA from three independent MD simulations. Values indicated are the mean and standard deviation.

Complex	ΔG (kcal/mol)
APC11/Emi1	-35.2 (12.5)
APC11/UBE2C	-36.1 (4.7)
RBX1/GLMN	-68.6 (10.2)

Table S2. Per-residue binding energy contribution calculated with MM-GBSA with data extracted from three independent MD simulations of APC11 in complex with the *de novo* designed BH, HT and HTH molecules. Values indicated are the mean and standard deviation.

APC11 residues	*Per residue* binding energy contribution (kcal/mol)							
	BH1	BH2	BH3	BH4	HT1	HT2	HTH1	HTH2
I25	-1.009 (0.773)	-1.088 (0.833)	-1.575 (0.204)	-1.044 (0.270)	-1.286 (0.963)	-1.739 (0.639)	-2.135 (0.560)	-2.317 (1.097)
C26	-1.661 (1.078)	-1.398 (0.905)	-1.625 (1.067)	-1.484 (0.486)	-0.571 (0.553)	-1.257 (0.348)	-1.717 (0.919)	-1.562 (0.842)
R27	-1.789 (1.571)	-3.887 (0.929)	-1.411 (1.727)	-3.025 (1.351)	-1.126 (1.557)	-0.979 (0.101)	-1.930 (0.893)	-4.500 (1.771)
M28	-1.733 (1.262)	-0.915 (0.580)	-0.660 (0.626)	-1.681 (0.620)	-0.212 (0.251)	-0.739 (0.292)	-0.251 (0.080)	-0.658 (0.550)
P35	-1.683 (1.040)	-0.511 (0.625)	-1.013 (1.032)	-1.141 (0.563)	-0.511 (0.625)	-1,729 (1.104)	-1.135 (0.588)	-1.533 (1.416)
D36	-0.398 (0.457)	0.023 (0.082)	-0.362 (0.375)	0.019 (0.029)	0.129 (0.302)	0.446 (0.271)	-2.407 (1.926)	-1.349 (1.696)
W63	-1.162 (1.280)	-1.382 (0.306)	-2.158 (0.645)	-1.180 (0.454)	-2.669 (1.057)	-2.026 (0.271)	-2.994 (0.443)	-3.051 (1.345)
A66	-0.138 (0.238)	-0.015 (0.024)	-1.321 (1.442)	-0.018 (0.015)	-1.202 (0.760)	-1.174 (0.605)	-0.888 (0.146)	-0.839 (0.746)
Q67	-0.160 (0.306)	-0.104 (0.210)	-0.641 (0.503)	-0.204 (0.431)	-1.094 (1.195)	-0.198 (0.201)	-0.126 (0.091)	-0.268 (0.494)
P74	-0.258 (0.263)	-0.594 (0.149)	-0.246 (0.171)	-0.471 (0.122)	-0.276 (0.217)	-0.169 (0.092)	-0.608 (0.273)	-0.691 (0.843)
R77	-0.863 (1.592)	-1.952 (2.508)	0.292 (0.559)	-2.343 (2.334)	-0.854 (1.836)	-0.210 (0.581)	-1.291 (1.017)	-1.348 (1.972)

Table S3. MM-GBSA binding free energies obtained with data from three independent MD simulations of RBX1 in complex with the *de novo* designed BH, HT and HTH molecules. Values indicated are the mean and standard deviation.

E2 mimetic molecules	ΔG (kcal/mol)
BH1	-20.9 (2.3)
BH2	-20.4 (4.6)
BH3	-26.1 (1.8)
BH4	-23.3 (2.3)
HT1	-23.6 (1.6)
HT2	-22.8 (2.5)
HTH1	-25.3 (4.3)
HTH2	-22.3 (3.2)

Table S4. Per-residue binding energy contribution calculated with MM-GBSA from three independent MD simulations of APC11 in complex with the *de novo* designed H molecules. Values indicated are the mean and standard deviation.

APC11 residues	*Per residue* binding energy contribution (kcal/mol)					
	H1	H2	H3	H4	H5	H6
I25	-2.172 (0.654)	-2.147 (0.260)	-2.236 (0.243)	-2.708 (1.125)	-2.649 (1.163)	-2.589 (0.573)
C26	-3.004 (0.347)	-2.125 (0.396)	-1.713 (1.366)	-2.901 (1.005)	-2.216 (0.847)	-1.915 (0.483)
R27	-0.981 (0.742)	-1.211 (0.244)	-1.225 (0.583)	-0.326 (0.771)	-1.379 (2.415)	-3.642 (1.682)
M28	-1.682 (0.544)	-1.747 (0.325)	-1.275 (1.165)	-1.378 (0.395)	-0.524 (0.347)	-0.502 (0.278)
P35	-2.911 (1.128)	-2.008 (0.621)	-2.081 (1.689)	-2.358 (0.227)	-2.524 (1.325)	-1.363 (0.540)
D36	-2.375 (1.846)	-1.901 (1.710)	-0.142 (0.647)	0.091 (0.374)	-1.618 (1.163)	-0.636 (0.574)
W63	-4.512 (0.404)	-2.705 (0.676)	-3.655 (0.938)	-3.460 (0.677)	-2.815 (0.483)	-2.385 (0.475)
A66	-1.509 (0.427)	-1.751 (0.635)	-1.737 (0.625)	-1.140 (0.083)	-0.796 (0.399)	-1.292 (0.413)
Q67	-2.046 (0.318)	-2.073 (1.214)	-1.762 (0.869)	-1.214 (1.088)	-0.210 (0.561)	-0.165 (0.143)
P74	-0.767 (0.705)	-0.255 (0.160)	-0.836 (0.845)	-1.201 (0.604)	-1.517 (0.349)	-0.349 (0.186)
R77	0.702 (0.106)	-3.720 (2.855)	-0.277 (1.364)	-1.551 (1.536)	-0.429 (1.102)	0.280 (0.145)

Table S5. MM-GBSA binding free energies obtained with data from three independent MD simulations of RBX1 in complex with the *de novo* designed H molecules. Values indicated are the mean and standard deviation.

H molecules	ΔG (kcal/mol)
H1	-20.8 (2.7)
H2	-26.5 (2.2)
H3	-17.7 (7.8)
H4	-19.7 (5.1)
H5	-14.8 (6.6)
H6	-23.5 (5.0)

Table S6. rpHPLC retention times (R_t) and molecular weights of ^{i}APC11 provided by Genscript Biotech (Leiden, Netherlands) B. V. determined by analytical rpHPLC and electronspray mass spectrometry, respectively.

Compound	Sequence	R_t (min)	$[M + XH]^{x+}$				Calculated mass
			x = 1	x = 2	x = 3	x = 4	
BH1	Ac-RCHWGPETFWCR-NH_2	18.3[a]	1617.5	809.3	539.9	---	1616.85
BH2	Ac-RCSWGPETFWCR-NH_2	19.6[a]	1567.4	784.3	---		1566.79
BH3	Ac-RCHWGPRTFWCR-NH_2	17.3[a]	---	822.8	548.9	412.0	1643.92
Pra-BH3	Ac-PraRCHWGPRTFWCR-NH_2	13.2[a]	---	870.3	580.6	435.6	1739.01
BH4	GRCHWGPETFWCRT	16.3[a]	1734.7	867.9	578.9	434.5	1733.95
HT1	Ac-CEAGPSQWHCTWRY-NH_2	17.5[a]	1763.5	882.3	---	--	1762.95
HT2	Ac-CEAGPSQWHCEWRY-NH_2	15.1[a]	1791.5	896.3	---	---	1790.96
HTH1	Ac-THCDDPETWRWNCQRV-NH_2	15.2[a]	---	1043.4	696.0	---	2085.27
HTH2	GDTCDDPETWRWNCQRH	15.8[a]	---	1059.5	706.7	---	2117.22
H1	Ac-RWPQXKWDXQVRRW-NH_2	11.7[b]	---	1017.0	678.3	509.0	2032.20
Pra-H1	Ac-RWPPraXKWDXQVRRW-NH_2	17.3[a]	---	1000.4	667.4	500.8	1998.99
H2	Ac-REPQXIKDXQVRRW-NH_2	15.4[a]	---	951.9	635.0	476.5	1902.27
H3	Ac-RWPQXIKDXQVRWW-NH_2	17.7[a]	---	995.5	664.0	---	1989.39
H4	Ac-EWWZKLEIQRXRRA-NH_2	13.8[b]	---	1003.2	669.4	502.2	2004.37
H5	Ac-EWWZKLELQRXRRA-NH_2	19.2[a]	---	1003.1	669.0	502.0	2004.37
H6	Ac-EWWZIKEIQRXRRA-NH_2	21.7[a]	---	1003.0	669.0	502.0	2004.37
Pra-H6	Ac-EWPraZIKEIQRXRRA-NH_2	13.9[a]	---	---	638.6	479.2	1913.31
Pra-$_{EE1}$H6	Ac-GFWFG-$(PEG)_6$-EWPraZIKEIQRXRRA-NH_2	12.8[a]	---	---	948.8	711.8	2843.30
$_{EE2}$H6	Ac-GFWFG-$NH(CH_2)_2OCH_2CO$-EWWZIKEIQRXRRA-NH_2	19.3[a]	---	1350.8	900.8	675.8	2700.14
Ng	Ac-RRKZAQERARXVRY-NH_2	16.1[a]	---	961.9	641.6	481.5	1922.27
Pra-Ng	Ac-RRPraZAQERARXVRY-NH_2	18.0[a]	---	945.6	630.6	473.3	1889.23

[a]Linear gradient from 5% to 65% solvent B over 25 min. [b]Linear gradient from 15% to 75% solvent B over 25 min. Solvent A is 0.065% TFA in 100% water (v/v) and solvent B is 0.05% TFA in 100% acetonitrile (v/v). In peptide sequences, underlined C indicate disulfide bond, X refers to (*S*)-2-(4'-pentenyl)Ala, Z refers to (*R*)-2-(7'-octenyl)Ala and underlined Z and X denote a hydrocarbon-staple.

Purities and chromatograms of ^{i}APC11

BH1

Pump A : 0.065% trifluoroacetic in 100% water (v/v)
Pump B : 0.05% trifluoroacetic in 100% acetonitrile (v/v)
Total Flow:1 ml/min
Wavelength:220 nm

Time	Unit	Command	Value	Comment
0.01	Pumps	Pump A B.Conc	5	
25.00	Pumps	Pump A B.Conc	65	
25.01	Pumps	Pump A B.Conc	95	
31.00	Pumps	Pump A B.Conc	95	
31.01	Pumps	Pump A B.Conc	5	
40.00	Pumps	Pump A B.Conc	5	
40.01	Controller	Stop		

<<Column Performance>>
<Detector A>
Column : AlltimaTM C18 4.6 x 250 mm

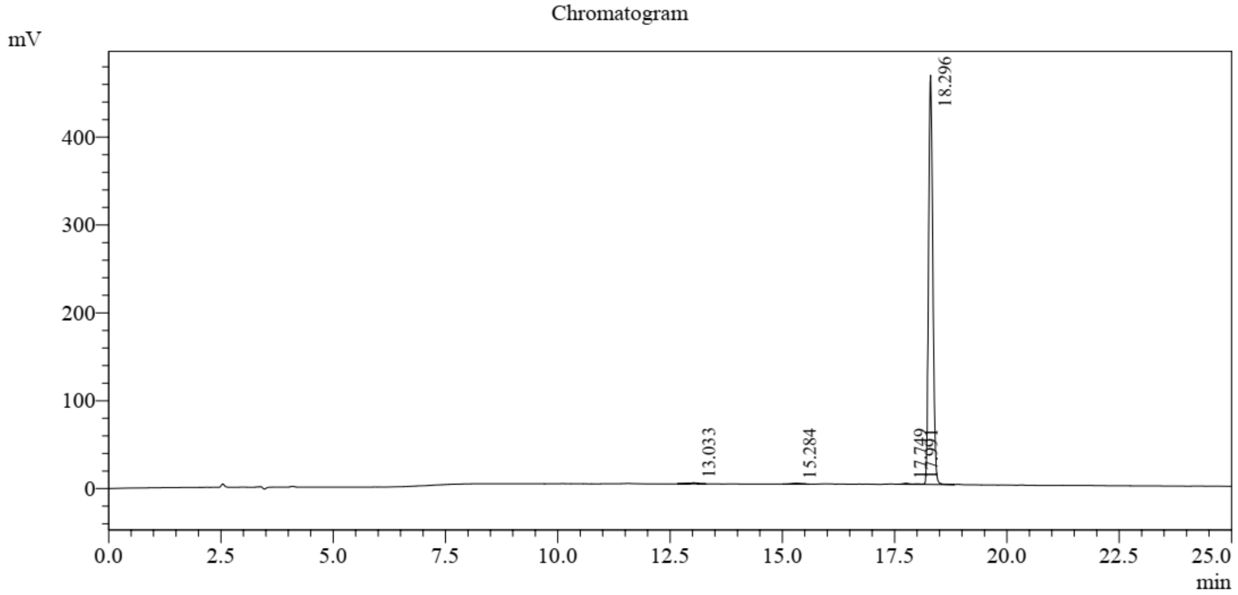

1 Det.A Ch1 / 220nm

Peak Table

Detector A Ch1 220nm

Peak#	Ret. Time	Area	Height	Area %
1	13.033	13182	1184	0.424
2	15.284	14790	951	0.475
3	17.749	9839	1021	0.316
4	17.991	5409	518	0.174
5	18.296	3068900	465587	98.611
Total		3112120	469261	100.000

BH2

Pump A : 0.065% trifluoroacetic in 100% water (v/v)
Pump B : 0.05% trifluoroacetic in 100% acetonitrile (v/v)
Total Flow:1 ml/min
Wavelength:220 nm

Time	Unit	Command	Value	Comment
0.01	Pumps	Pump A B.Conc	5	
25.00	Pumps	Pump A B.Conc	65	
25.01	Pumps	Pump A B.Conc	95	
31.00	Pumps	Pump A B.Conc	95	
31.01	Pumps	Pump A B.Conc	5	
40.00	Pumps	Pump A B.Conc	5	
40.01	Controller	Stop		

<<Column Performance>>
<Detector A>
Column : AlltimaTM C18 4.6 x 250 mm

Chromatogram

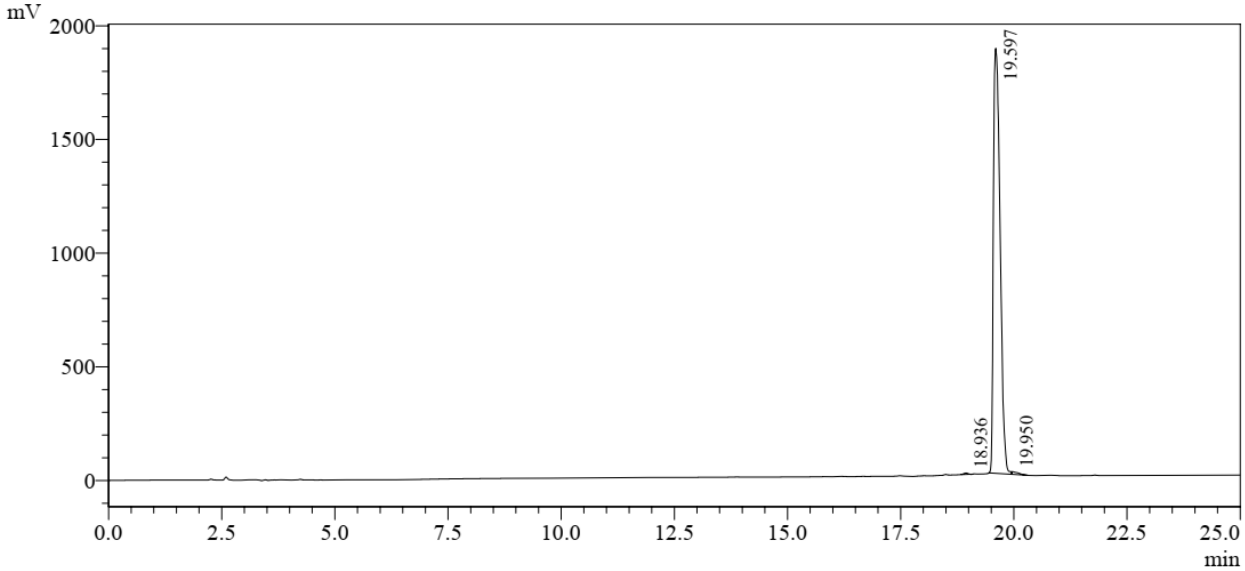

1 Det.A Ch1 / 220nm

Peak Table

Detector A Ch1 220nm

Peak#	Ret. Time	Area	Height	Area %
1	18.936	37990	6134	0.196
2	19.597	19206046	1869480	99.209
3	19.950	115133	11588	0.595
Total		19359169	1887202	100.000

BH3

Pump A : 0.065% trifluoroacetic in 100% water (v/v)
Pump B : 0.05% trifluoroacetic in 100% acetonitrile (v/v)
Total Flow:1 ml/min
Wavelength:220 nm

Time	Unit	Command	Value	Comment
0.01	Pumps	Pump A B.Conc	5	
25.00	Pumps	Pump A B.Conc	65	
25.01	Pumps	Pump A B.Conc	95	
31.00	Pumps	Pump A B.Conc	95	
31.01	Pumps	Pump A B.Conc	5	
40.00	Pumps	Pump A B.Conc	5	
40.01	Controller	Stop		

<<Column Performance>>
<Detector A>
Column : AlltimaTM C18 4.6 x 250 mm

Chromatogram

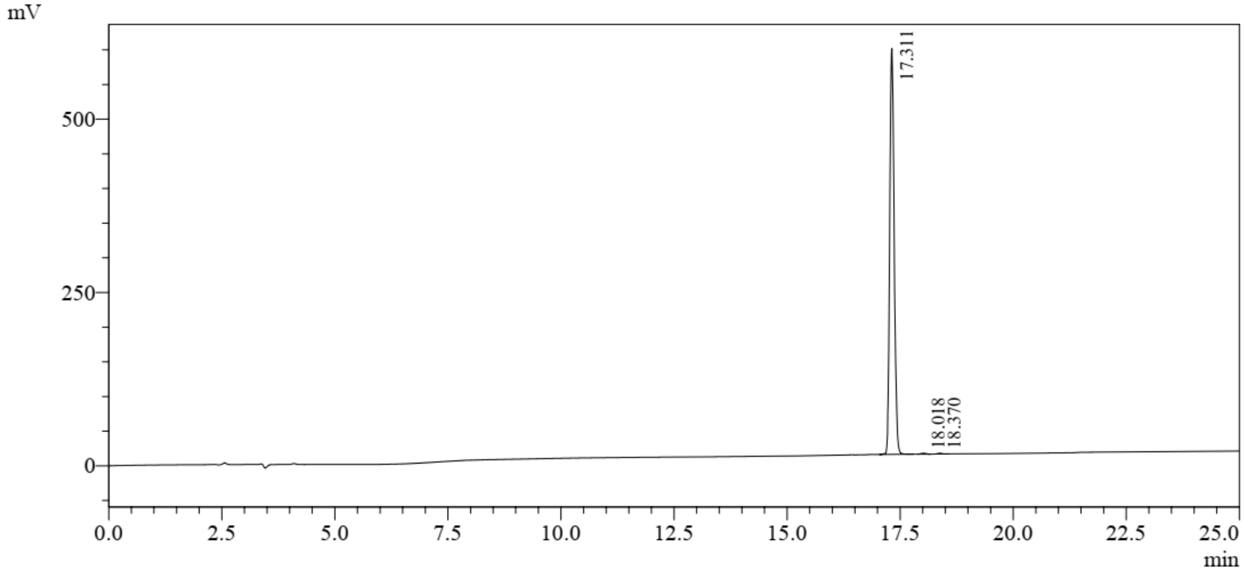

1 Det.A Ch1 / 220nm

Peak Table

Detector A Ch1 220nm

Peak#	Ret. Time	Area	Height	Area %
1	17.311	4017387	585291	99.593
2	18.018	9066	1233	0.225
3	18.370	7355	1140	0.182
Total		4033808	587665	100.000

Pra-BH3

Pump A : 0.065% trifluoroacetic in 100% water (v/v)
Pump B : 0.05% trifluoroacetic in 100% acetonitrile (v/v)
Total Flow:1 ml/min
Wavelength:220 nm

Time	Module	Command	Value
0.01	Pumps	Pump A B.Conc	5
25.00	Pumps	Pump A B.Conc	65
25.01	Pumps	Pump A B.Conc	95
27.00	Pumps	Pump A B.Conc	95
27.01	Pumps	Pump A B.Conc	5
35.00	Pumps	Pump A B.Conc	5
35.01	Controller	Stop	

<<Column Performance>>
<Detector A>
Column : Inertsil ODS-3 4.6 x 250 mm

Chromatogram

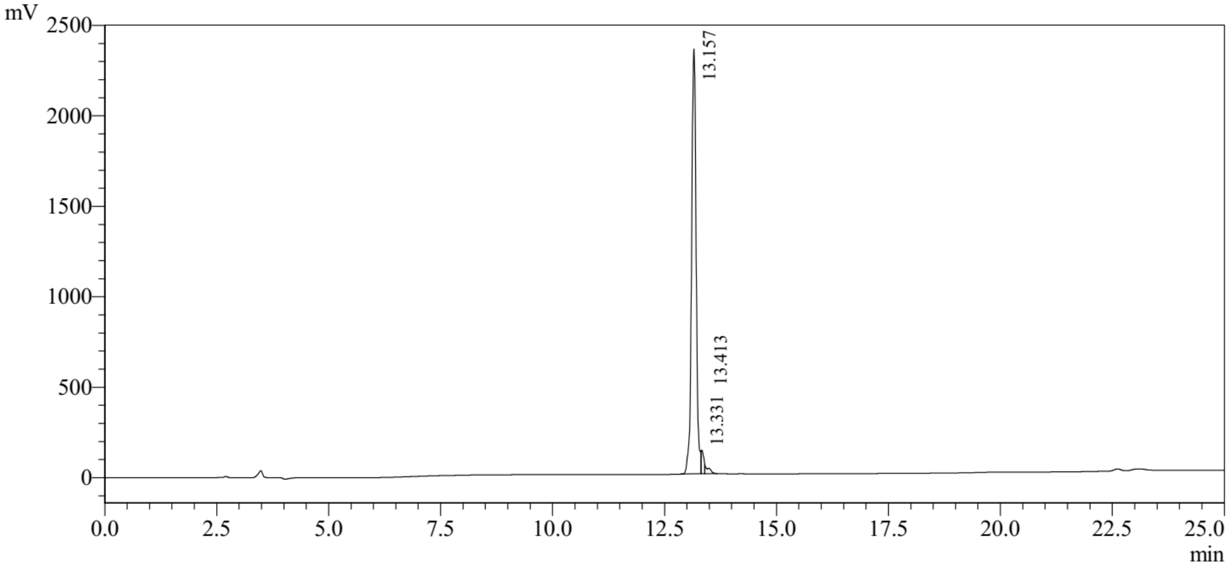

1 Detector A Channel 1 / 220nm

Peak Table

Detector A Channel 1 220nm

Peak#	Ret. Time	Area	Height	Area %
1	13.157	16762511	2348023	95.559
2	13.331	506129	130509	2.885
3	13.413	272968	44922	1.556
Total		17541609	2523454	100.000

BH4

Pump A : 0.065% trifluoroacetic in 100% water (v/v)
Pump B : 0.05% trifluoroacetic in 100% acetonitrile (v/v)
Total Flow:1 ml/min
Wavelength:220 nm

Time	Unit	Command	Value	Comment
0.01	Pumps	Pump A B.Conc	5	
25.00	Pumps	Pump A B.Conc	65	
25.01	Pumps	Pump A B.Conc	95	
31.00	Pumps	Pump A B.Conc	95	
31.01	Pumps	Pump A B.Conc	5	
40.00	Pumps	Pump A B.Conc	5	
40.01	Controller	Stop		

<<Column Performance>>
<Detector A>
Column : AlltimaTM C18 4.6 x 250 mm

Chromatogram

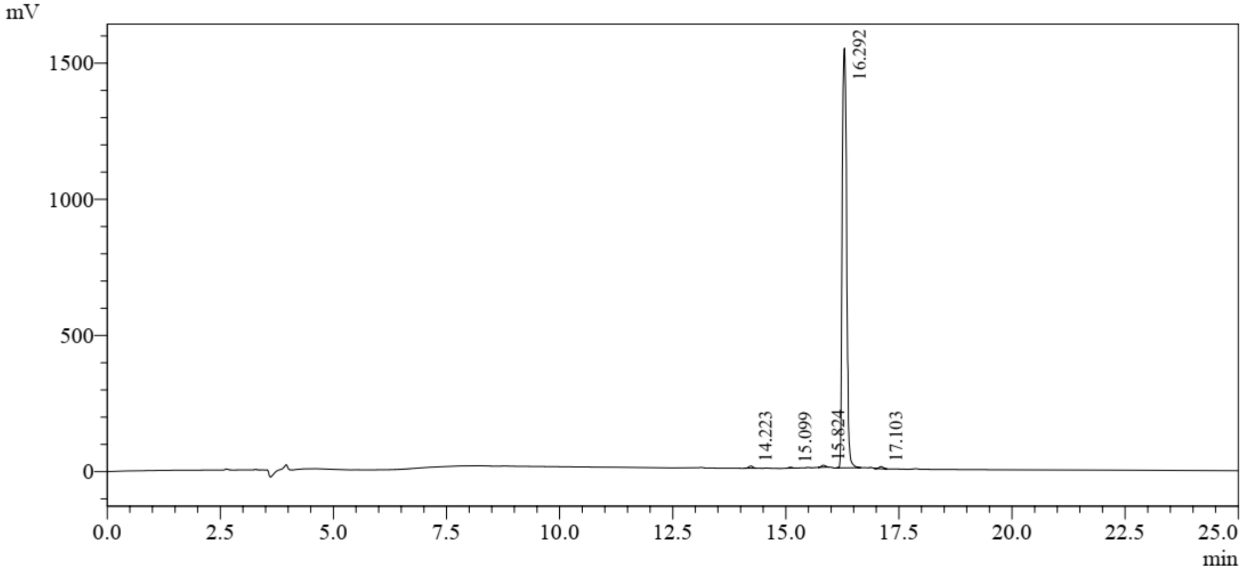

1 Det.A Ch1 / 220nm

Peak Table

Detector A Ch1 220nm

Peak#	Ret. Time	Area	Height	Area %
1	14.223	50040	8027	0.470
2	15.099	17482	3229	0.164
3	15.824	47715	7344	0.449
4	16.292	10475514	1540638	98.468
5	17.103	47792	7821	0.449
Total		10638542	1567059	100.000

HT1

Pump A : 0.065% trifluoroacetic in 100% water (v/v)
Pump B : 0.05% trifluoroacetic in 100% acetonitrile (v/v)
Total Flow:1 ml/min
Wavelength:220 nm

Time	Unit	Command	Value	Comment
0.01	Pumps	Pump A B.Conc	5	
25.00	Pumps	Pump A B.Conc	65	
25.01	Pumps	Pump A B.Conc	95	
31.00	Pumps	Pump A B.Conc	95	
31.01	Pumps	Pump A B.Conc	5	
40.00	Pumps	Pump A B.Conc	5	
40.01	Controller	Stop		

<<Column Performance>>
<Detector A>
Column : AlltimaTM C18 4.6 x 250 mm

Chromatogram

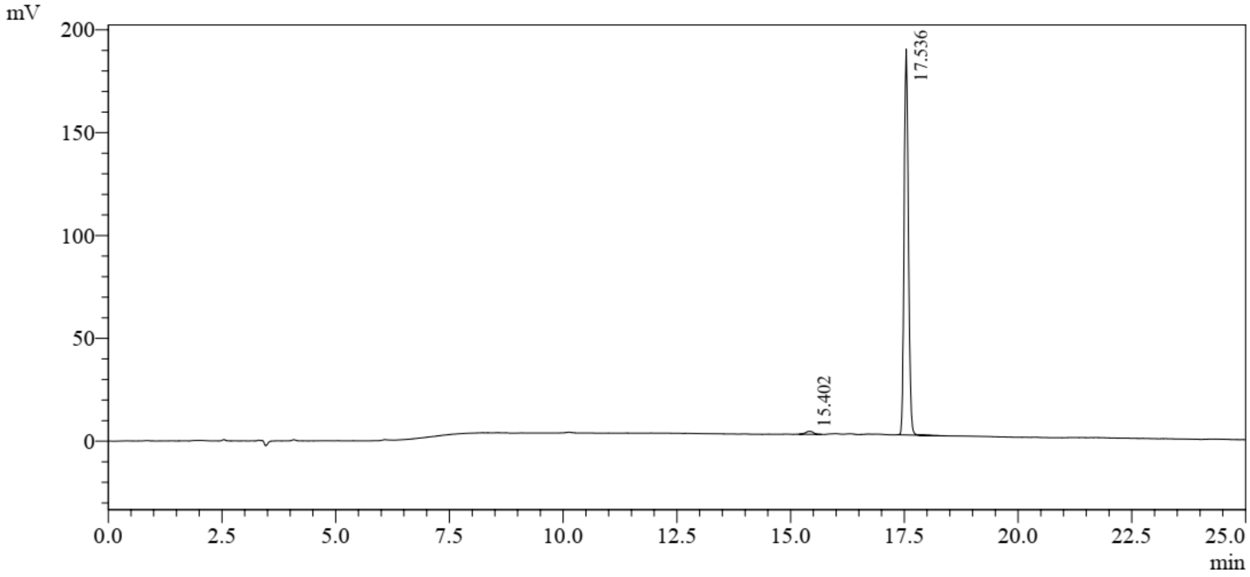

1 Det.A Ch1 / 220nm

Peak Table

Detector A Ch1 220nm

Peak#	Ret. Time	Area	Height	Area %
1	15.402	17232	1433	1.393
2	17.536	1220163	187492	98.607
Total		1237396	188926	100.000

HT2

Pump A : 0.065% trifluoroacetic in 100% water (v/v)
Pump B : 0.05% trifluoroacetic in 100% acetonitrile (v/v)
Total Flow:1 ml/min
Wavelength:220 nm

Time	Unit	Command	Value	Comment
0.01	Pumps	Pump A B.Conc	5	
25.00	Pumps	Pump A B.Conc	65	
25.01	Pumps	Pump A B.Conc	95	
31.00	Pumps	Pump A B.Conc	95	
31.01	Pumps	Pump A B.Conc	5	
40.00	Pumps	Pump A B.Conc	5	
40.01	Controller	Stop		

<<Column Performance>>
<Detector A>
Column : AlltimaTM C18 4.6 x 250 mm

Chromatogram

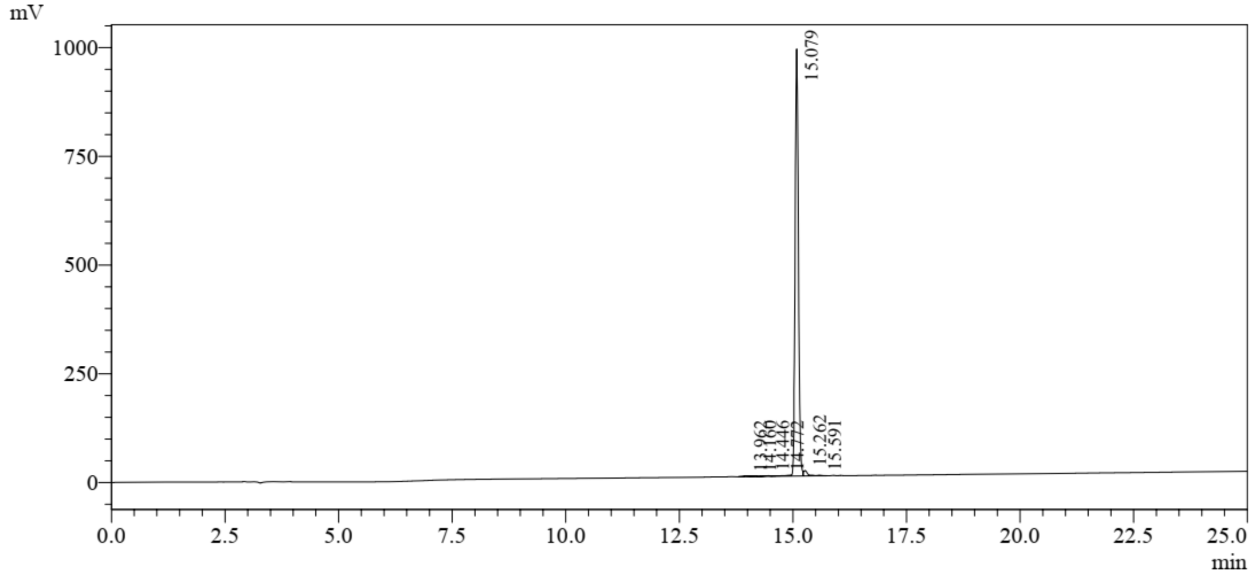

1 Det.A Ch1 / 220nm

Peak Table

Detector A Ch1 220nm

Peak#	Ret. Time	Area	Height	Area %
1	13.962	11306	1570	0.224
2	14.160	7589	797	0.150
3	14.446	14036	1731	0.278
4	14.772	17091	1345	0.338
5	15.079	4912110	981952	97.288
6	15.262	77612	12510	1.537
7	15.591	9296	1383	0.184
Total		5049040	1001288	100.000

HTH1

Pump A : 0.065% trifluoroacetic in 100% water (v/v)
Pump B : 0.05% trifluoroacetic in 100% acetonitrile (v/v)
Total Flow:1 ml/min
Wavelength:220 nm

Time	Unit	Command	Value	Comment
0.01	Pumps	Pump B Conc.	5	
25.00	Pumps	Pump B Conc.	65	
25.01	Pumps	Pump B Conc.	95	
31.00	Pumps	Pump B Conc.	95	
31.01	Pumps	Pump B Conc.	5	
40.00	Pumps	Pump B Conc.	5	
40.01	Controller	Stop		

<<Column Performance>>
<Detector A>
Column : AlltimaTM C18 4.6 x 250 mm

Chromatogram

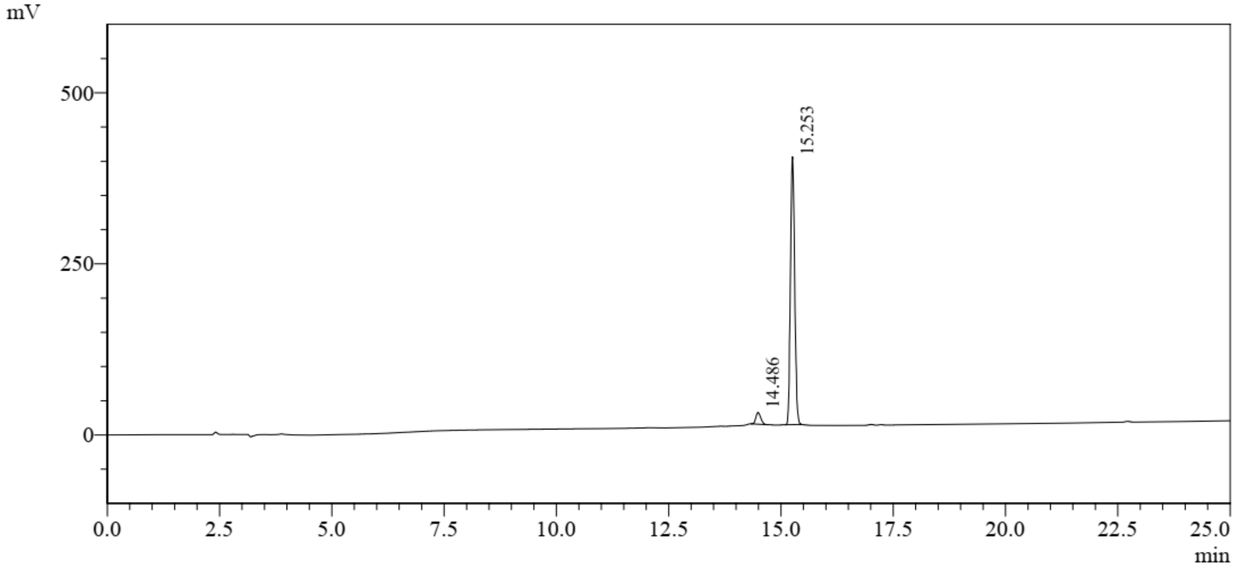

1 Det.A Ch1 / 220nm

Peak Table

Detector A Ch1 220nm

Peak#	Ret. Time	Area	Height	Area %
1	14.486	129685	17185	4.833
2	15.253	2553678	391357	95.167
Total		2683363	408542	100.000

HTH2

Pump A : 0.065% trifluoroacetic in 100% water (v/v)
Pump B : 0.05% trifluoroacetic in 100% acetonitrile (v/v)
Total Flow:1 ml/min
Wavelength:220 nm

Time	Unit	Command	Value	Comment
0.01	Pumps	Pump A B.Conc	5	
25.00	Pumps	Pump A B.Conc	65	
25.01	Pumps	Pump A B.Conc	95	
31.00	Pumps	Pump A B.Conc	95	
31.01	Pumps	Pump A B.Conc	5	
40.00	Pumps	Pump A B.Conc	5	
40.01	Controller	Stop		

<<Column Performance>>
<Detector A>
Column : AlltimaTM C18 4.6 x 250 mm

Chromatogram

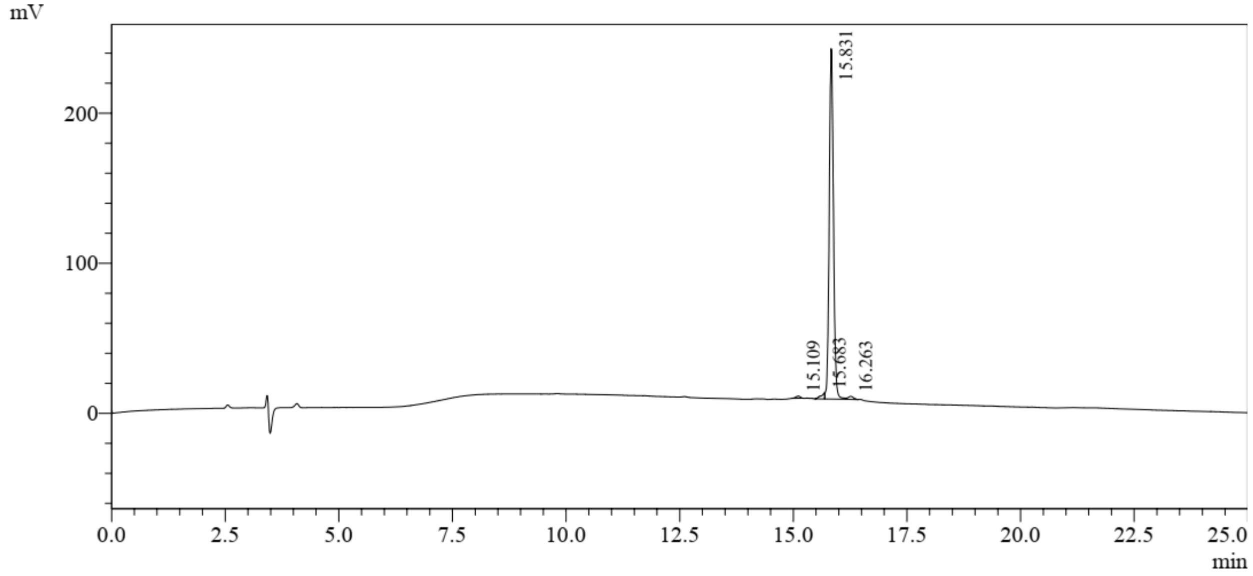

1 Det.A Ch1 / 220nm

Peak Table

Detector A Ch1 220nm

Peak#	Ret. Time	Area	Height	Area %
1	15.109	10168	1422	0.656
2	15.683	23502	4000	1.516
3	15.831	1499977	233466	96.750
4	16.263	16725	2032	1.079
Total		1550371	240919	100.000

H1

Pump A : 0.065% trifluoroacetic in 100% water (v/v)
Pump B : 0.05% trifluoroacetic in 100% acetonitrile (v/v)
Total Flow:1 ml/min
Wavelength:220 nm
<<LC Time Program>>

Time	Module	Command	Value
0.01	Pumps	Pump A B.Conc	15
25.00	Pumps	Pump A B.Conc	75
25.01	Pumps	Pump A B.Conc	95
31.00	Pumps	Pump A B.Conc	95
31.01	Pumps	Pump A B.Conc	15
40.00	Pumps	Pump A B.Conc	15
95.00	Controller	Stop	

<<Column Performance>>
<Detector A>
Column : AlltimaTM C18 4.6 x 250 mm
Equipment:ZJ19010324

<Chromatogram>

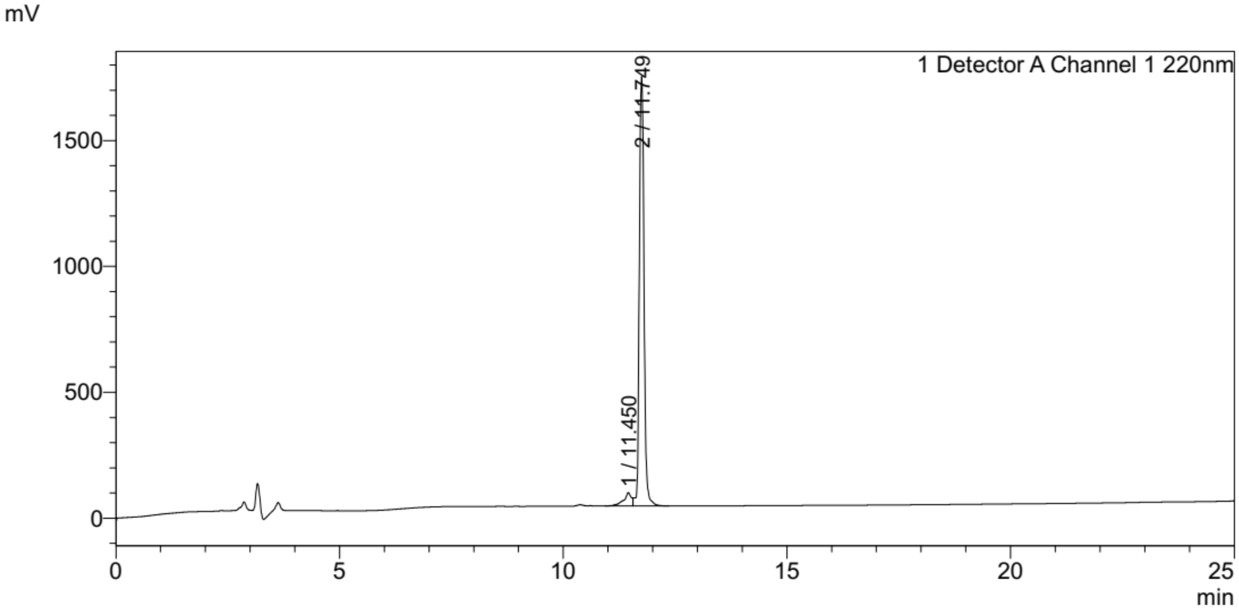

<Peak Table>

Detector A Channel 1 220nm

Peak#	Ret. Time	Area	Height	Area%
1	11.450	648557	53161	4.905
2	11.749	12573575	1706771	95.095
Total		13222133	1759932	100.000

Pra-H1

Pump A : 0.065% trifluoroacetic in 100% water (v/v)
Pump B : 0.05% trifluoroacetic in 100% acetonitrile (v/v)
Total Flow:1 ml/min
Wavelength:220 nm

Time	Unit	Command	Value	Comment
0.01	Pumps	Pump A B.Conc	5	
25.00	Pumps	Pump A B.Conc	65	
25.01	Pumps	Pump A B.Conc	95	
31.00	Pumps	Pump A B.Conc	95	
31.01	Pumps	Pump A B.Conc	5	
40.00	Pumps	Pump A B.Conc	5	
40.01	Controller	Stop		

<<Column Performance>>
<Detector A>
Column : Inertsil ODS-3 4.6 x 250 mm
Equipment: GR11010440

Chromatogram

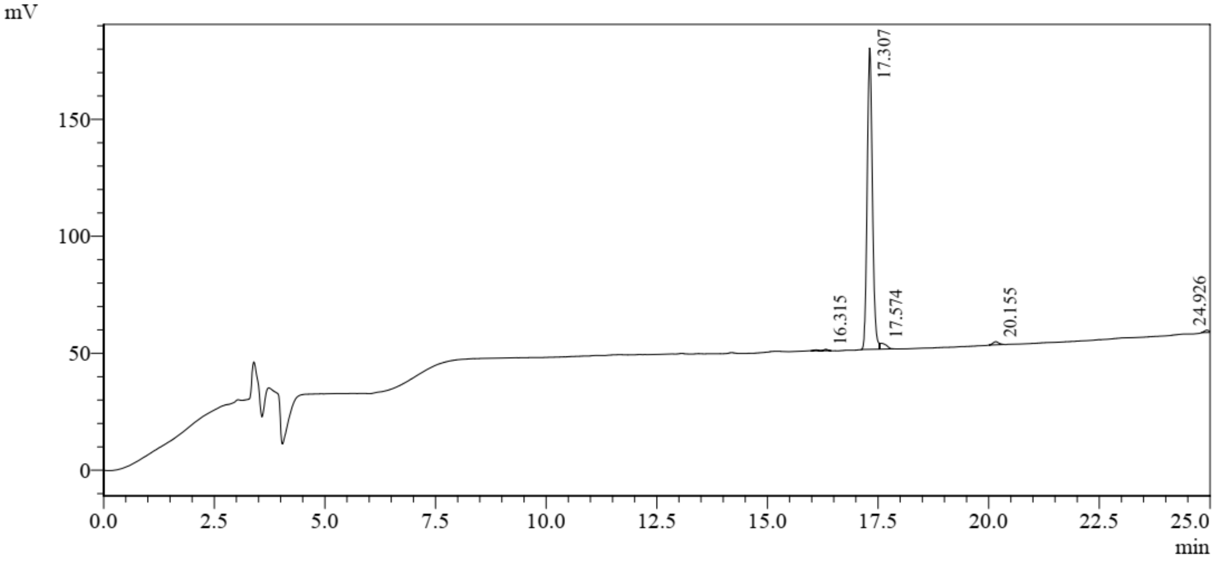

1 Det.A Ch1 / 220nm

Peak Table

Detector A Ch1 220nm

Peak#	Ret. Time	Area	Height	Area %
1	16.315	5995	591	0.544
2	17.307	1050712	128809	95.359
3	17.574	25210	2498	2.288
4	20.155	12388	1342	1.124
5	24.926	7543	968	0.685
Total		1101848	134208	100.000

H2

Pump A : 0.065% trifluoroacetic in 100% water (v/v)
Pump B : 0.05% trifluoroacetic in 100% acetonitrile (v/v)
Total Flow:1 ml/min
Wavelength:220 nm
<<LC Time Program>>

Time	Module	Command	Value
0.01	Pumps	Pump B Conc.	5
25.00	Pumps	Pump B Conc.	65
25.01	Pumps	Pump B Conc.	95
27.00	Pumps	Pump B Conc.	95
27.01	Pumps	Pump B Conc.	5
32.00	Pumps	Pump B Conc.	5
32.01	Controller	Stop	

<<Column Performance>>
<Detector A>
Column :Inertsil ODS-3 4.6 x 250 mm
Equipment:GK12010012

<Chromatogram>

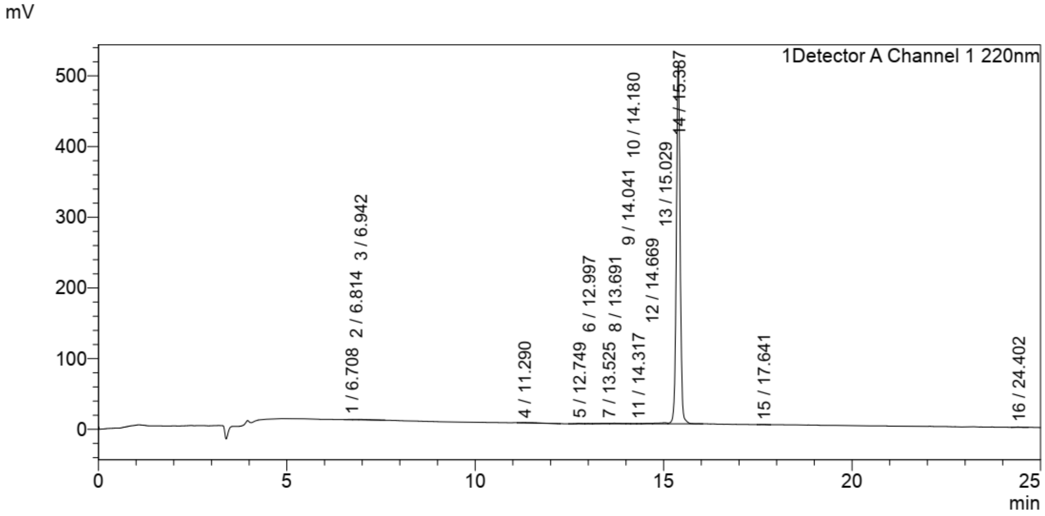

<Peak Table>

Detector A Channel 1 220nm

Peak#	Ret. Time	Area	Height	Area%
1	6.708	4254	450	0.115
2	6.814	5406	588	0.146
3	6.942	13701	664	0.370
4	11.290	23625	577	0.637
5	12.749	8904	713	0.240
6	12.997	5662	510	0.153
7	13.525	18433	823	0.497
8	13.691	19363	877	0.522
9	14.041	3774	525	0.102
10	14.180	4289	484	0.116
11	14.317	3159	418	0.085
12	14.669	12256	813	0.331
13	15.029	27502	1600	0.742
14	15.387	3549358	506826	95.736
15	17.641	3190	320	0.086

Peak#	Ret. Time	Area	Height	Area%
16	24.402	4570	458	0.123
Total		3707447	516647	100.000

H3

Pump A : 0.065% trifluoroacetic in 100% water (v/v)
Pump B : 0.05% trifluoroacetic in 100% acetonitrile (v/v)
Total Flow:1 ml/min
Wavelength:220 nm
<<LC Time Program>>

Time	Module	Command	Value
0.01	Pumps	Solvent B Conc.	5
25.00	Pumps	Solvent B Conc.	65
25.01	Pumps	Solvent B Conc.	95
27.00	Pumps	Solvent B Conc.	95
27.01	Pumps	Solvent B Conc.	5
33.00	Pumps	Solvent B Conc.	5
33.00	Controller	Stop	

<<Column Performance>>
<Detector A>
Column : AlltimaTM C18 4.6 x 250 mm
Equipment:ZJ19010325

<Chromatogram>

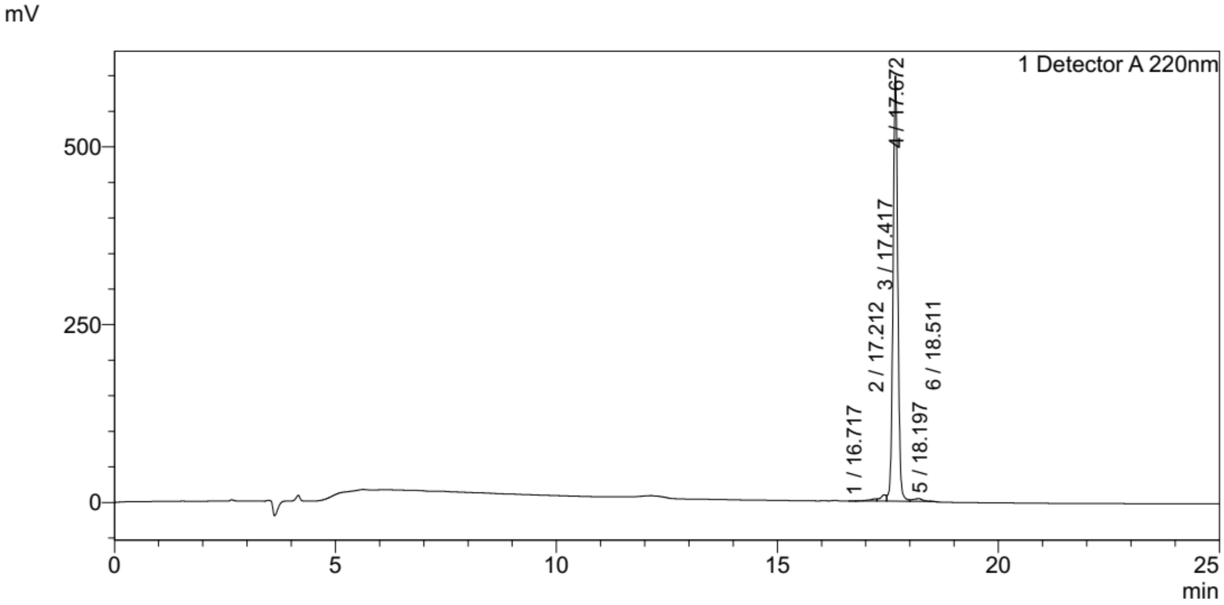

<Peak Table>

Detector A 220nm

Peak#	Ret. Time	Area	Height	Area%
1	16.717	1734	262	0.037
2	17.212	41007	3171	0.876
3	17.417	83806	8886	1.790
4	17.672	4496326	598221	96.061
5	18.197	55676	3968	1.189
6	18.511	2129	413	0.045
Total		4680679	614921	100.000

H4

Pump A : 0.065% trifluoroacetic in 100% water (v/v)
Pump B : 0.05% trifluoroacetic in 100% acetonitrile (v/v)
Total Flow:1 ml/min
Wavelength:220 nm

Time	Unit	Command	Value	Comment
0.01	Pumps	Pump A B.Conc	15	
25.00	Pumps	Pump A B.Conc	75	
25.01	Pumps	Pump A B.Conc	95	
32.00	Pumps	Pump A B.Conc	95	
32.01	Pumps	Pump A B.Conc	15	
40.00	Pumps	Pump A B.Conc	15	
40.00	Controller	Stop		

<<Column Performance>>
<Detector A>
Column : Inertsil ODS-3 4.6 x 250 mm
Equipment: GK1101009

Chromatogram

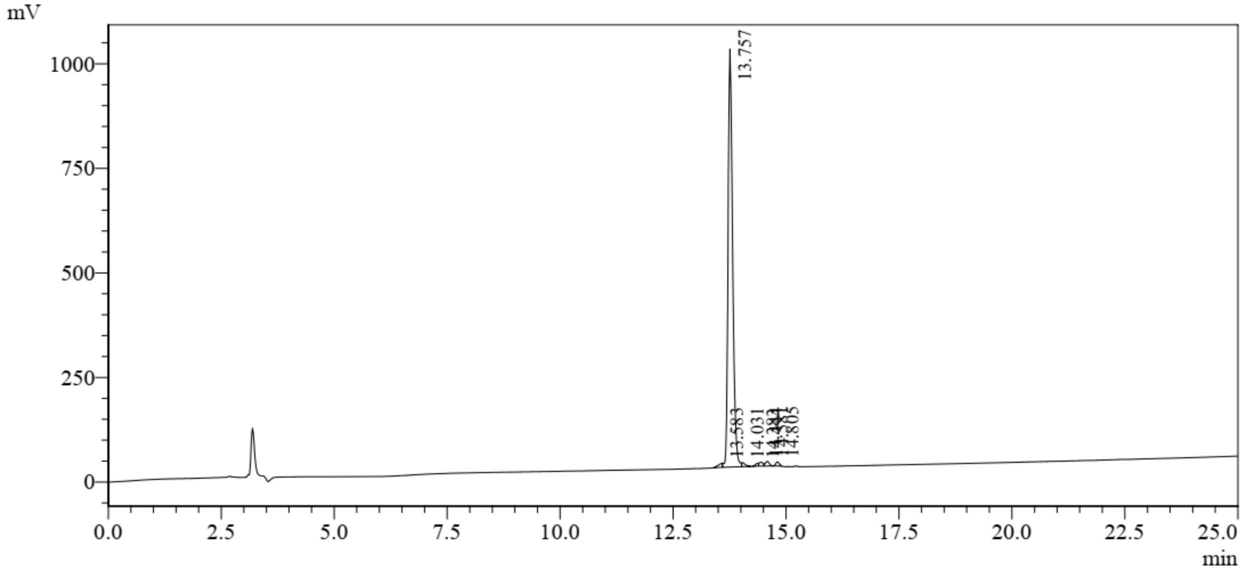

1 Det.A Ch1 / 220nm

Peak Table

Detector A Ch1 220nm

Peak#	Ret. Time	Area	Height	Area %
1	13.583	60620	9863	0.826
2	13.757	7019018	999615	95.677
3	14.031	49583	9531	0.676
4	14.383	31278	7006	0.426
5	14.444	56991	9349	0.777
6	14.581	65946	10886	0.899
7	14.805	52720	9628	0.719
Total		7336157	1055878	100.000

H5

Pump A : 0.065% trifluoroacetic in 100% water (v/v)
Pump B : 0.05% trifluoroacetic in 100% acetonitrile (v/v)
Total Flow:1 ml/min
Wavelength:220 nm
<<LC Time Program>>

Time	Module	Command	Value
0.01	Pumps	Pump B Conc.	5
25.00	Pumps	Pump B Conc.	65
25.01	Pumps	Pump B Conc.	95
27.00	Pumps	Pump B Conc.	95
27.01	Pumps	Pump B Conc.	5
32.00	Pumps	Pump B Conc.	5
32.01	Controller	Stop	

<<Column Performance>>
<Detector A>
Column :Inertsil ODS-3 4.6 x 250 mm
Equipment:GK12010012

<Chromatogram>

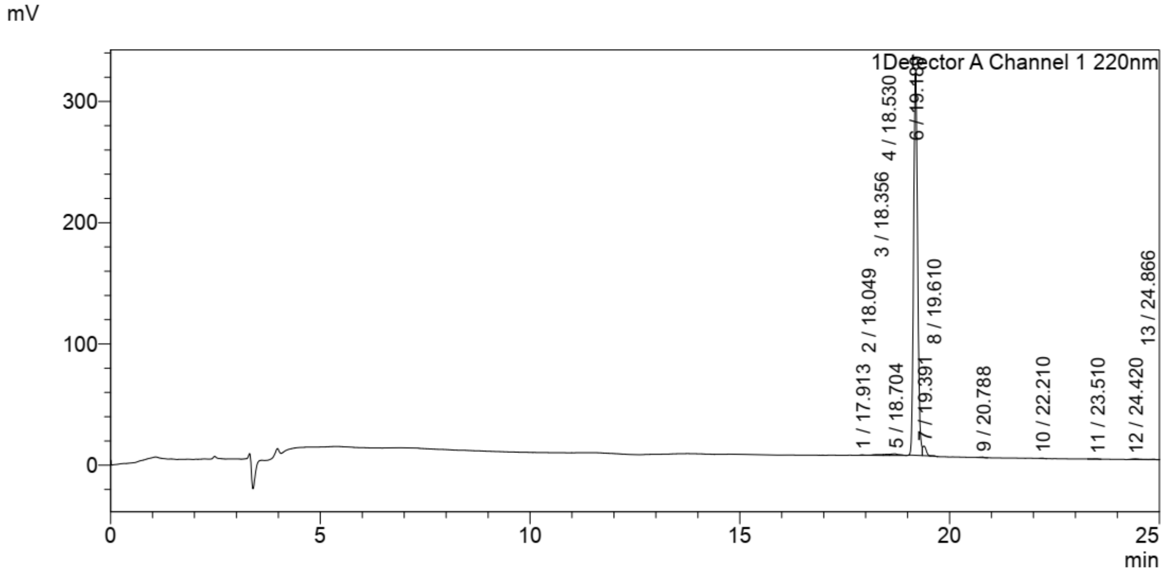

<Peak Table>

Detector A Channel 1 220nm

Peak#	Ret. Time	Area	Height	Area%
1	17.913	2447	396	0.111
2	18.049	1299	214	0.059
3	18.356	10243	671	0.465
4	18.530	7669	959	0.348
5	18.704	17681	1332	0.803
6	19.189	2097048	315203	95.274
7	19.391	44895	7868	2.040
8	19.610	1843	385	0.084
9	20.788	2963	429	0.135
10	22.210	1469	281	0.067
11	23.510	2918	346	0.133
12	24.420	7940	632	0.361
13	24.866	2658	349	0.121
Total		2201072	329065	100.000

H6

Pump A : 0.065% trifluoroacetic in 100% water (v/v)
Pump B : 0.05% trifluoroacetic in 100% acetonitrile (v/v)
Total Flow:1 ml/min
Wavelength:220 nm

Time	Unit	Command	Value	Comment
0.01	Pumps	Pump A B.Conc	5	
25.00	Pumps	Pump A B.Conc	65	
25.01	Pumps	Pump A B.Conc	95	
27.00	Pumps	Pump A B.Conc	95	
27.01	Pumps	Pump A B.Conc	5	
35.00	Pumps	Pump A B.Conc	5	
35.01	Controller	Stop		

<<Column Performance>>
<Detector A>
Column : Inertsil ODS-3 4.6 x 250 mm
Equipment: GR11010440

Chromatogram

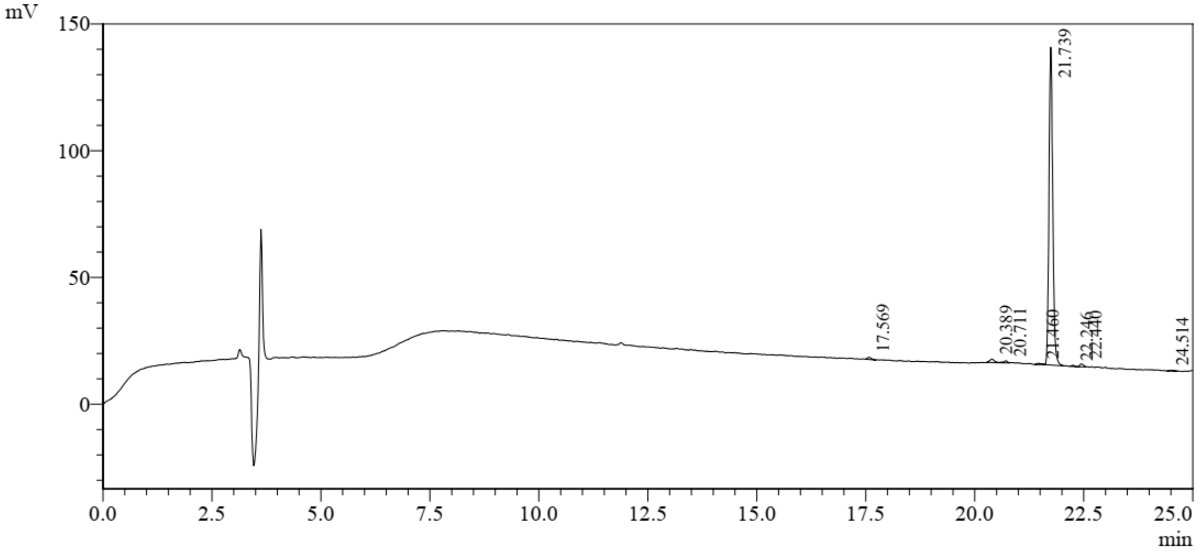

1 Det.A Ch1 / 220nm

Peak Table

Detector A Ch1 220nm

Peak#	Ret. Time	Area	Height	Area %
1	17.569	5578	893	0.698
2	20.389	10402	1396	1.301
3	20.711	5663	813	0.708
4	21.460	3955	490	0.495
5	21.739	762708	125356	95.406
6	22.246	2253	460	0.282
7	22.440	6598	1170	0.825
8	24.514	2274	308	0.284
Total		799432	130886	100.000

Pra-H6

Pump A : 0.065% trifluoroacetic in 100% water (v/v)
Pump B : 0.05% trifluoroacetic in 100% acetonitrile (v/v)
Total Flow:1 ml/min
Wavelength:220 nm
<<LC Time Program>>

Time	Module	Command	Value
0.01	Pumps	Pump A B.Conc	15
25.00	Pumps	Pump A B.Conc	75
25.01	Pumps	Pump A B.Conc	95
32.00	Pumps	Pump A B.Conc	95
32.01	Pumps	Pump A B.Conc	15
40.00	Pumps	Pump A B.Conc	15
40.00	Controller	Stop	

<<Column Performance>>
<Detector A>
Column : AlltimaTM C18 4.6 x 250 mm
Equipment:ZJ19010324

<Chromatogram>

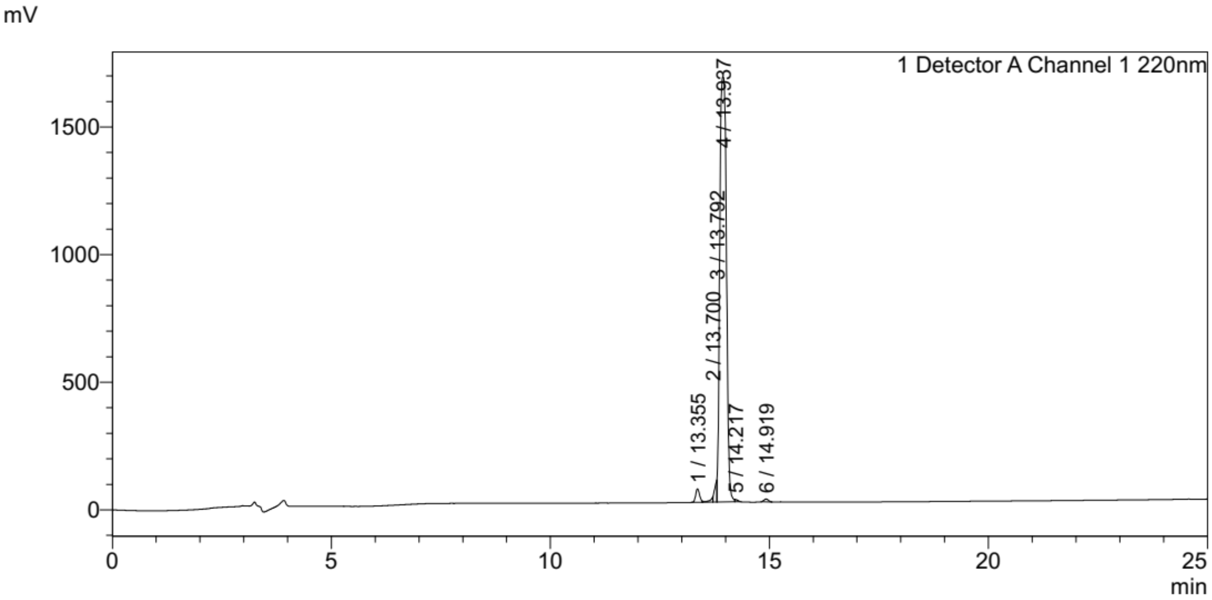

<Peak Table>

Detector A Channel 1 220nm

Peak#	Ret. Time	Area	Height	Area%
1	13.355	328303	53283	1.787
2	13.700	93376	18062	0.508
3	13.792	310643	79901	1.691
4	13.937	17527898	1667251	95.404
5	14.217	36066	8716	0.196
6	14.919	76090	10732	0.414
Total		18372377	1837946	100.000

Pra-$_{EE1}$H6

Pump A : 0.065% trifluoroacetic in 100% water (v/v)
Pump B : 0.05% trifluoroacetic in 100% acetonitrile (v/v)
Total Flow:1 ml/min
Wavelength:220 nm
<<LC Time Program>>

Time	Module	Command	Value
0.01	Pumps	Pump A B.Conc	5
25.00	Pumps	Pump A B.Conc	65
25.01	Pumps	Pump A B.Conc	95
31.00	Pumps	Pump A B.Conc	95
31.01	Pumps	Pump A B.Conc	5
40.00	Pumps	Pump A B.Conc	5
45.00	Controller	Stop	

<<Column Performance>>
<Detector A>
Column : AlltimaTM C18 4.6 x 250 mm
Equipment:ZJ19010324

<Chromatogram>

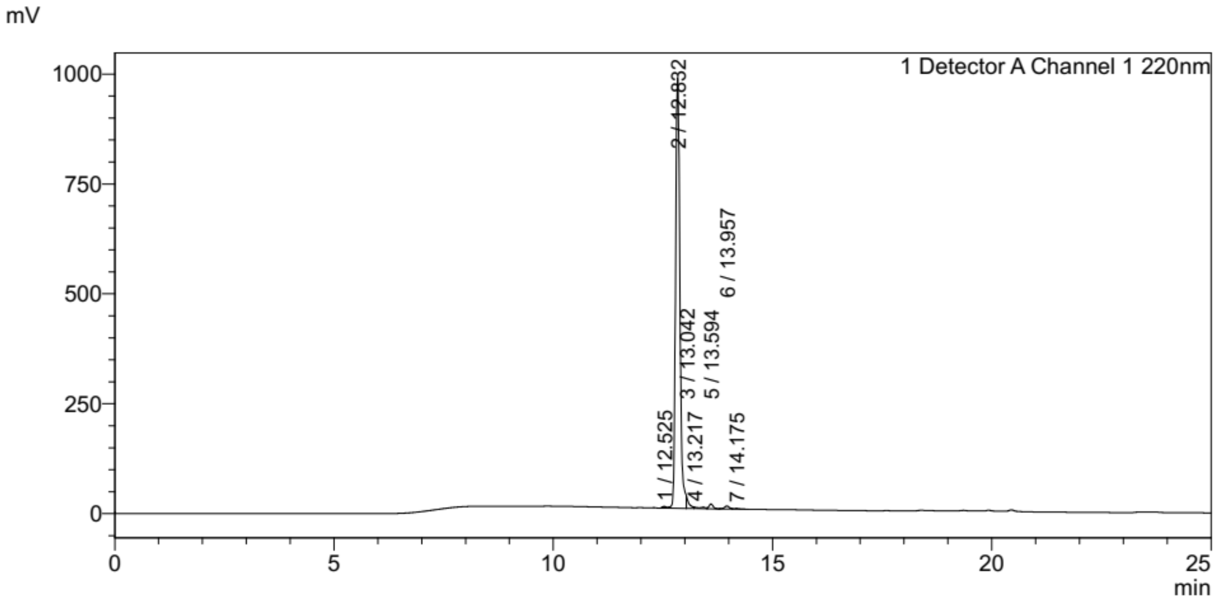

<Peak Table>

Detector A Channel 1 220nm

Peak#	Ret. Time	Area	Height	Area%
1	12.525	27300	3991	0.383
2	12.832	6819266	980259	95.590
3	13.042	106555	26088	1.494
4	13.217	42568	3159	0.597
5	13.594	72355	10922	1.014
6	13.957	54538	6899	0.764
7	14.175	11315	1284	0.159
Total		7133897	1032601	100.000

$_{EE2}$**H6**

Pump A : 0.065% trifluoroacetic in 100% water (v/v)
Pump B : 0.05% trifluoroacetic in 100% acetonitrile (v/v)
Total Flow:1 ml/min
Wavelength:220 nm
<<LC Time Program>>

Time	Module	Command	Value
0.01	Pumps	Pump A B.Conc	5
25.00	Pumps	Pump A B.Conc	65
25.01	Pumps	Pump A B.Conc	95
31.00	Pumps	Pump A B.Conc	95
31.01	Pumps	Pump A B.Conc	5
40.00	Pumps	Pump A B.Conc	5
40.01	Controller	Stop	

<<Column Performance>>
<Detector A>
Column : Inertsil ODS-3 4.6 x 250 mm
Equipment:ZJ19010324

<Chromatogram>

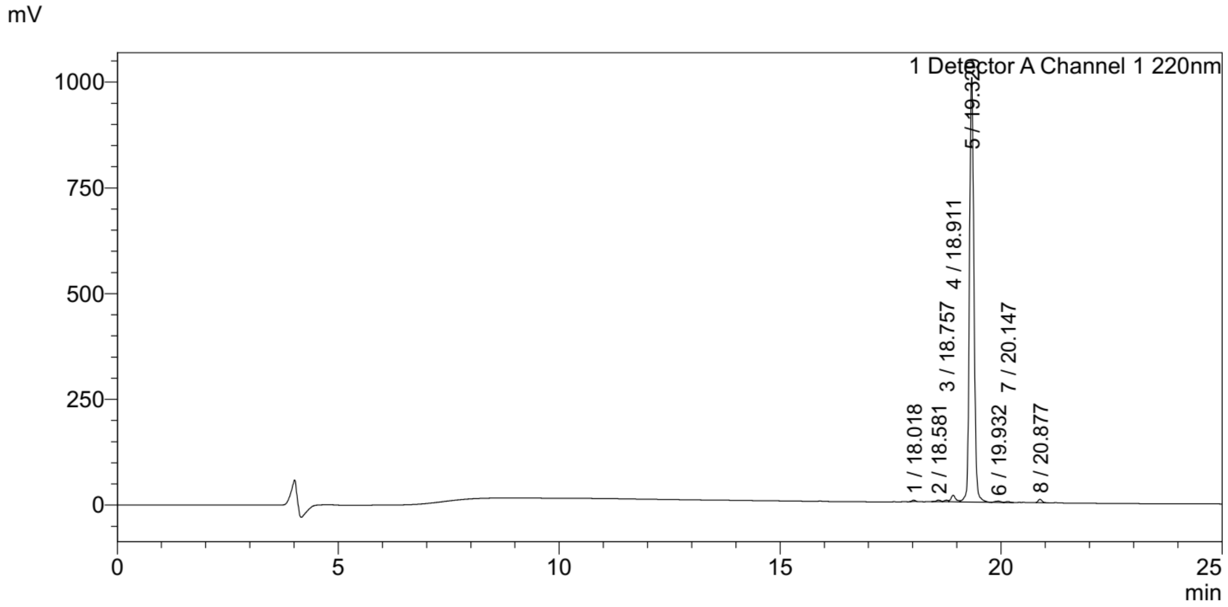

<Peak Table>

Detector A Channel 1 220nm

Peak#	Ret. Time	Area	Height	Area%
1	18.018	14524	3064	0.199
2	18.581	22560	3680	0.309
3	18.757	22184	3748	0.304
4	18.911	116432	15710	1.596
5	19.329	7032143	1004107	96.400
6	19.932	26199	2888	0.359
7	20.147	14186	2254	0.194
8	20.877	46544	7865	0.638
Total		7294772	1043315	100.000

Ng

Pump A : 0.065% trifluoroacetic in 100% water (v/v)
Pump B : 0.05% trifluoroacetic in 100% acetonitrile (v/v)
Total Flow:1 ml/min
Wavelength:220 nm
<<LC Time Program>>

Time	Module	Command	Value
0.01	Pumps	Solvent B Conc.	5
25.00	Pumps	Solvent B Conc.	65
25.01	Pumps	Solvent B Conc.	95
31.00	Pumps	Solvent B Conc.	95
31.01	Pumps	Solvent B Conc.	5
40.00	Pumps	Solvent B Conc.	5
95.00	Controller	Stop	

<<Column Performance>>
<Detector A>
Column : Inertsil ODS-3 4.6 x 250 mm
Equipment:ZJ19010324

<Chromatogram>

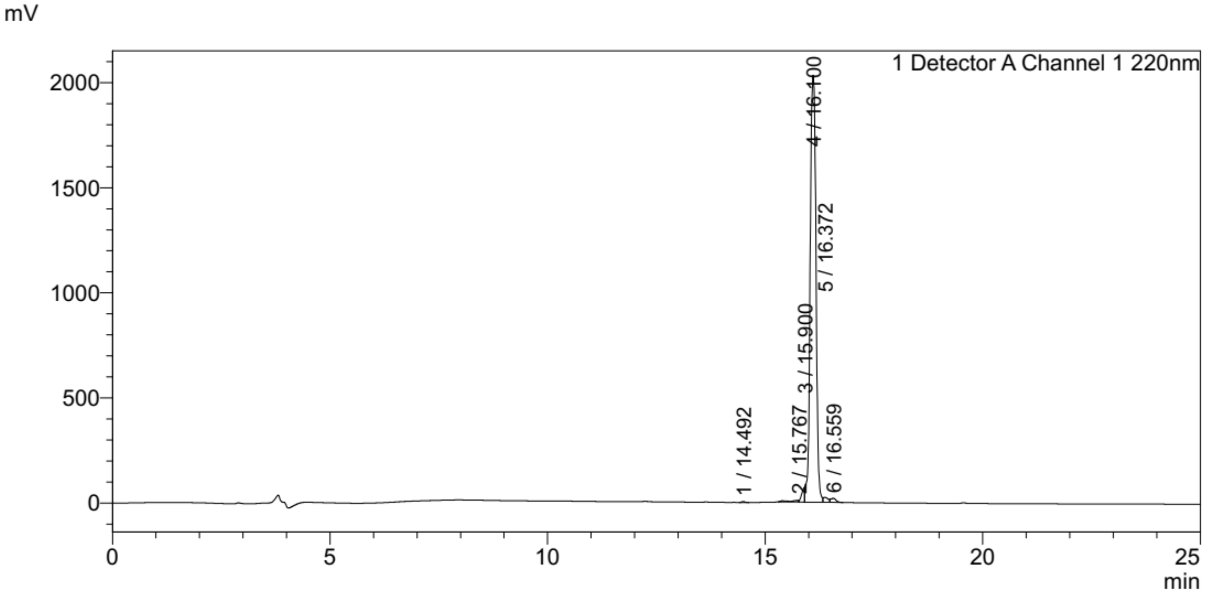

<Peak Table>

Detector A Channel 1 220nm

Peak#	Ret. Time	Area	Height	Area%
1	14.492	27992	4829	0.144
2	15.767	120026	7114	0.617
3	15.900	325088	68830	1.670
4	16.100	18642013	2031009	95.783
5	16.372	184021	23239	0.946
6	16.559	163594	19057	0.841
Total		19462734	2154078	100.000

Pra-Ng

Pump A : 0.065% trifluoroacetic in 100% water (v/v)
Pump B : 0.05% trifluoroacetic in 100% acetonitrile (v/v)
Total Flow:1 ml/min
Wavelength:220 nm
<<LC Time Program>>

Time	Module	Command	Value
0.01	Pumps	Pump A B.Conc	5
25.00	Pumps	Pump A B.Conc	65
25.01	Pumps	Pump A B.Conc	95
32.00	Pumps	Pump A B.Conc	95
32.01	Pumps	Pump A B.Conc	5
40.00	Pumps	Pump A B.Conc	5
40.10	Controller	Stop	

<<Column Performance>>
<Detector A>
Column : Inertsil ODS-3 4.6 x 250 mm
Equipment:ZJ17010507

<Chromatogram>

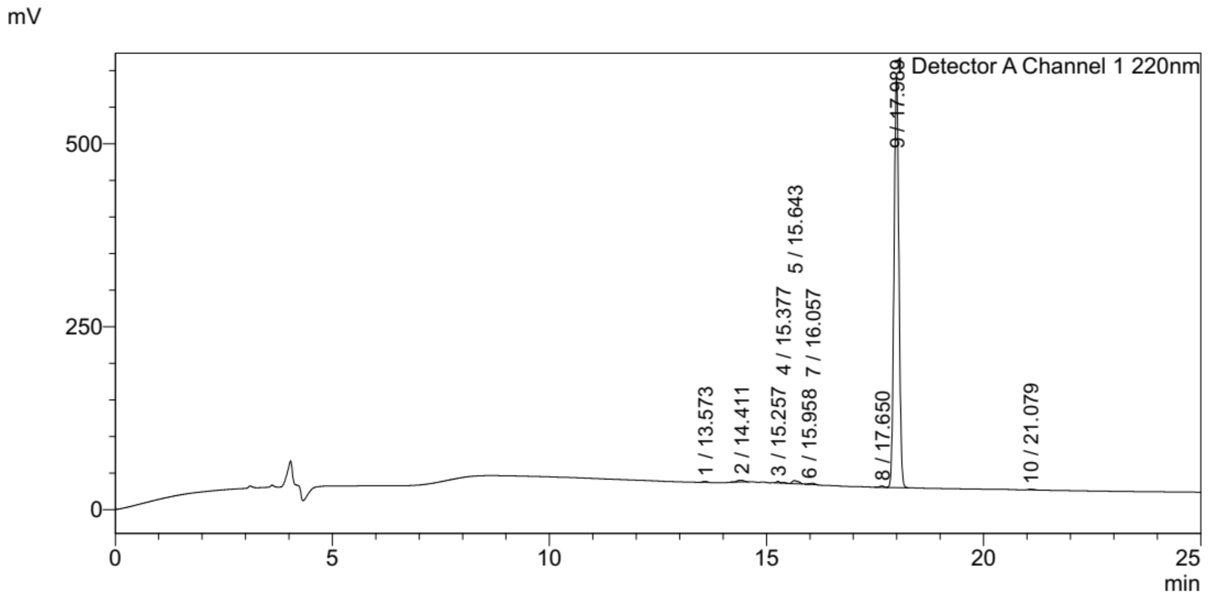

<Peak Table>

Detector A Channel 1 220nm

Peak#	Ret. Time	Area	Height	Area%
1	13.573	5239	1198	0.117
2	14.411	23900	2275	0.532
3	15.257	9739	1991	0.217
4	15.377	6122	1195	0.136
5	15.643	41794	4308	0.930
6	15.958	683	319	0.015
7	16.057	12447	1675	0.277
8	17.650	11990	1836	0.267
9	17.989	4378020	560543	97.463
10	21.079	2048	327	0.046
Total		4491982	575667	100.000

References

1. Zhang, Z.; Yang, J.; Kong, E. H.; Chao, W. C.; Morris, E. P.; da Fonseca, P. C.; Barford, D., Recombinant expression, reconstitution and structure of human anaphase-promoting complex (APC/C). *Biochem. J.* **2013,** *449* (2), 365-71.
2. Simpson, R. J., Disruption of cultured cells by nitrogen cavitation. *Cold Spring Harb. Protoc.* **2010,** *2010* (11), pdb prot5513.
3. Izawa, D.; Pines, J., Mad2 and the APC/C compete for the same site on Cdc20 to ensure proper chromosome segregation. *J. Cell Biol.* **2012,** *199* (1), 27-37.
4. Bakos, G.; Yu, L.; Gak, I. A.; Roumeliotis, T. I.; Liakopoulos, D.; Choudhary, J. S.; Mansfeld, J., An E2-ubiquitin thioester-driven approach to identify substrates modified with ubiquitin and ubiquitin-like molecules. *Nat. Commun.* **2018,** *9* (1), 4776.
5. Brown, N. G.; Watson, E. R.; Weissmann, F.; Jarvis, M. A.; VanderLinden, R.; Grace, C. R. R.; Frye, J. J.; Qiao, R.; Dube, P.; Petzold, G.; Cho, S. E.; Alsharif, O.; Bao, J.; Davidson, I. F.; Zheng, J. J.; Nourse, A.; Kurinov, I.; Peters, J. M.; Stark, H.; Schulman, B. A., Mechanism of polyubiquitination by human anaphase-promoting complex: RING repurposing for ubiquitin chain assembly. *Mol. Cell* **2014,** *56* (2), 246-260.
